# A high-fidelity CRISPR-Cas13 system improves abnormalities associated with C9ORF72-linked ALS/FTD

**DOI:** 10.1038/s41467-024-55548-5

**Published:** 2025-01-08

**Authors:** Tristan X. McCallister, Colin K. W. Lim, Mayuri Singh, Sijia Zhang, Najah S. Ahsan, William M. Terpstra, Alisha Y. Xiong, M. Alejandra Zeballos C, Jackson E. Powell, Jenny Drnevich, Yifei Kang, Thomas Gaj

**Affiliations:** 1 Department of Bioengineering, The Grainger College of Engineering, University of Illinois Urbana-Champaign, Urbana, IL USA; 2 High-Performance Biological Computing, Roy J. Carver Biotechnology Center, University of Illinois Urbana-Champaign, Urbana, IL USA; 3 Carl R. Woese Institute for Genomic Biology, University of Illinois Urbana-Champaign, Urbana, IL USA

**Keywords:** Genetic engineering, CRISPR-Cas9 genome editing, Amyotrophic lateral sclerosis

## Abstract

An abnormal expansion of a GGGGCC (G_4_C_2_) hexanucleotide repeat in the *C9ORF72* gene is the most common genetic cause of amyotrophic lateral sclerosis (ALS) and frontotemporal dementia (FTD), two debilitating neurodegenerative disorders driven in part by gain-of-function mechanisms involving transcribed forms of the repeat expansion. By utilizing a Cas13 variant with reduced collateral effects, we develop here a high-fidelity RNA-targeting CRISPR-based system for C9ORF72-linked ALS/FTD. When delivered to the brain of a transgenic rodent model, this Cas13-based platform curbed the expression of the G_4_C_2_ repeat-containing RNA without affecting normal C9ORF72 levels, which in turn decreased the formation of RNA foci, reduced the production of a dipeptide repeat protein, and reversed transcriptional deficits. This high-fidelity system possessed improved transcriptome-wide specificity compared to its native form and mediated targeting in motor neuron-like cells derived from a patient with ALS. These results lay the foundation for the implementation of RNA-targeting CRISPR technologies for C9ORF72-linked ALS/FTD.

## Abstract (web summary)

An expansion of a hexanucleotide repeat in the C9ORF72 gene can cause ALS. Here, the authors develop a high-fidelity RNA-targeting CRISPR system to suppress the repeat RNA expressed from the C9ORF72 gene, which improved abnormalities in a mouse model.

## Introduction

Amyotrophic lateral sclerosis (ALS) is a rapidly progressive, paralytic, and invariably fatal disorder characterized by the loss of motor neurons in the brain and spinal cord^1^. It also exists on a continuum of conditions that includes frontotemporal dementia (FTD)^2–4^, a syndrome defined by the progressive impairment of cognitive functions due to the degeneration of the frontal and temporal lobes of the brain^5,6^.

An abnormal expansion of a GGGGCC (G_4_C_2_) hexanucleotide repeat in the first intron of the chromosome 9 open-reading frame 72 (*C9ORF72*) gene is the most common genetic cause of both ALS and FTD^7,8^. To date, three non-exclusive mechanisms have been proposed to explain the pathogenicity of this repeat expansion^9–24^. These include a loss-of-function of the C9ORF72 protein from impaired transcription of the mutant allele^9–11^ and/or an acquired gain-of-function from the bidirectional transcription of sense^12^ and antisense^13^ transcripts carrying the repeat expansion, which can accumulate in cells as foci^14^, potentially with critical RNA-binding proteins^15–18^. These repeat-containing transcripts can further serve as templates for the synthesis of one of five dipeptide repeat (DPR) proteins^19–22^, which are non-canonically translated^19^ and believed to exert toxic effects on cells^23,24^.

Given the evidence in support of a role for the hexanucleotide repeat-containing RNAs in C9ORF72-linked ALS and FTD – hereafter referred to as C9-ALS/FTD – strategies for silencing their production hold potential for the disorder^25–30^, as they can inhibit the formation of the abnormal RNA foci^25,27^ and DPR proteins^31,32^. One emerging technology with the capabilities to enable this is Cas13^33–35^, a class 2 type VI CRISPR effector protein that, when complexed with a CRISPR RNA (crRNA) guide molecule carrying complementarity to a target transcript, can cleave it via its intrinsic ribonuclease (RNase) activity. To date, four distinct Cas13 subtypes have been identified and used to perturb gene expression in eukaryotic cells^33–36^. Among these is the Cas13d nuclease from *Ruminococcus flavefaciens* XPD3002^33^, known as RfxCas13d or CasRx, a CRISPR effector protein that is compact enough to fit within a single adeno-associated virus (AAV) vector to facilitate its delivery to the central nervous system^37–40^ and whose modification with nuclear localization signal (NLS) sequences can enable it to target transcripts in the nucleus^33^. Given these properties, we hypothesized that RfxCas13d could be used to suppress the hexanucleotide repeat-containing RNA and influence pathological hallmarks of C9-ALS/FTD.

 Here, we establish a CRISPR-Cas13-based platform for C9-ALS/FTD. Using a dual-luciferase reporter screen designed to measure target engagement and collateral effects, we identified crRNAs for RfxCas13d that facilitated the efficient targeting of the G_4_C_2_ repeat-containing RNA. When delivered to C9-BACexp mice, which harbor the human *C9ORF72* gene with a disease-associated repeat expansion, these RfxCas13d-based platforms curbed the expression of the G_4_C_2_ repeat RNA without affecting normal C9ORF72 mRNA levels, an outcome that led to a reduction in RNA foci composed of a transcribed form of the repeat expansion. Further, we demonstrate that a high-fidelity variant of RfxCas13d with improved transcriptome-wide specificity can be used to target the G_4_C_2_ repeat RNA in motor neuron-like cells derived from a patient with ALS and C9-BACexp mice, where it inhibited the formation of RNA foci, decreased the production of a DPR protein, and reversed transcriptional deficits following its in vivo delivery.

Altogether, our findings illustrate the potential of CRISPR-Cas13 technology for C9-ALS/FTD.

## Results

### Programming RfxCas13d to target C9ORF72

**Fig. 1:**
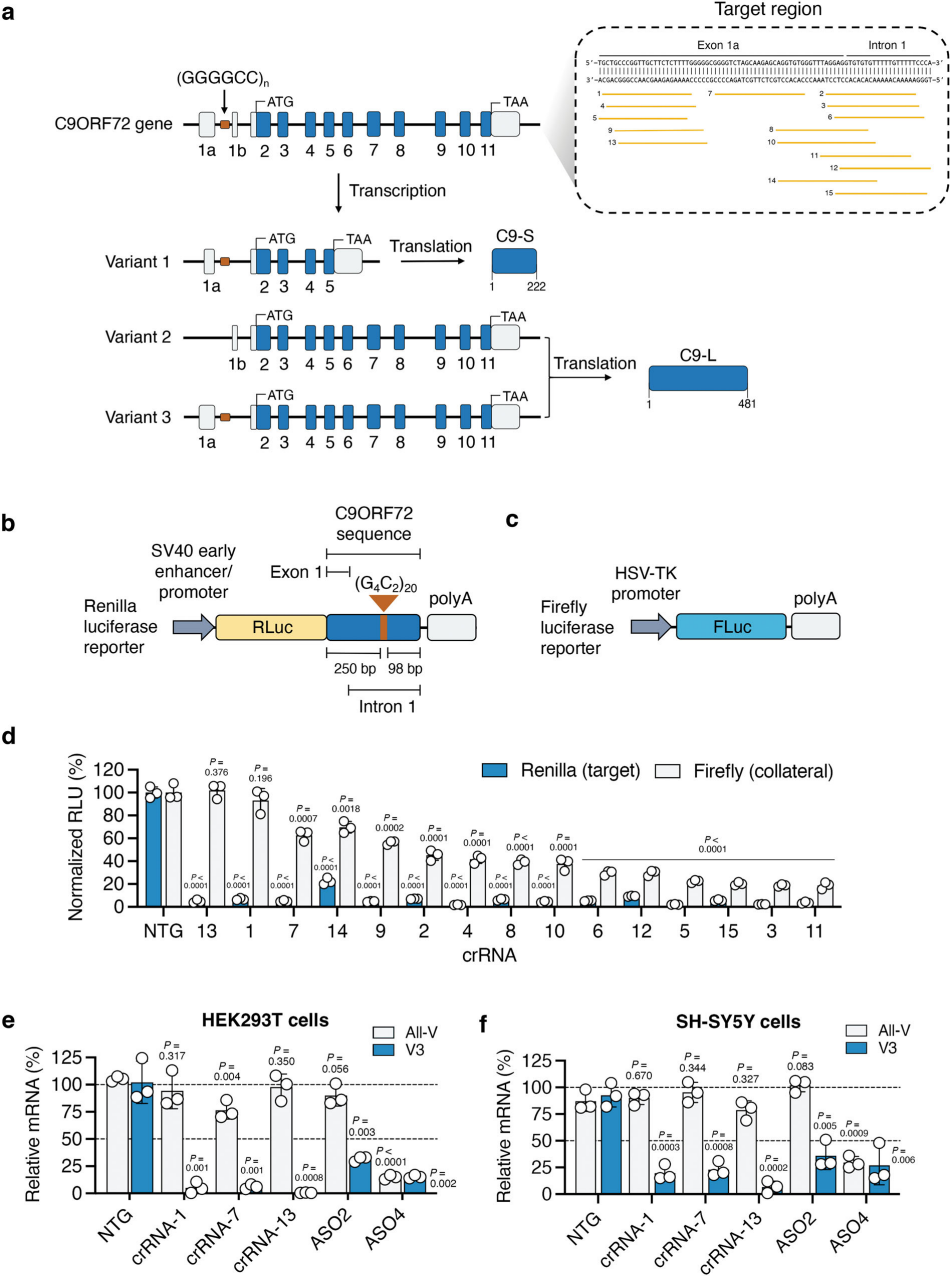
RfxCas13d can be programmed to target C9ORF72. **a** Schematic of (top) the *C9ORF72* gene and (bottom) the three main transcript variants expressed from it. V1 produces the short protein isoform (C9-S), while V2 and V3 produce the long protein isoform (C9-L). The inset shows the locations of the crRNA binding sites for RfxCas13d in exon 1a and intron 1a of the C9ORF72 transcript. **b**, **c** Schematic of the dual-reporter system used to evaluate crRNAs. The platform consists of a **b** Renilla luciferase-encoding plasmid, pSV40-RLuc, whose 3’ untranslated region (UTR) carries a fragment of C9ORF72 with 20 copies of the G_4_C_2_ repeat and 250- and 98-base pairs (bps) of the flanking upstream and downstream sequences, respectively, and **c** a firefly luciferase-encoding plasmid, pHSV-TK-FLuc, that was used as a proxy for collateral cleavage. **d** Relative Renilla and firefly luciferase luminescence in HEK293T cells co-transfected with pSV40-RLuc, pHSV-TK-FLuc, and an expression vector encoding RfxCas13d and one of the 15 candidate crRNAs. All values normalized to cells transfected with pSV40-RLuc, pHSV-TK-FLuc, and an expression vector encoding RfxCas13d with a non-targeted (NTG) crRNA (*n* = 3). Relative all-V and V3 mRNA in **e** HEK293T and **f** SH-SY5Y cells transfected with RfxCas13d and crRNAs 13, 7, and 1 or a NTG crRNA or one of two ASOs (*n* = 3). All-V and V3 for each crRNA and ASO normalized to untreated cells. Values indicate means and error bars indicate SD. **d** Renilla and firefly luciferase luminesence for each crRNA compared to NTG using a one-tailed unpaired t-test, with exact P values shown. **e**, **f** All-V and V3 for each cRNA and ASO compared to NTG using a two-tailed unpaired t-test, with exact P values shown. All data points are biologically independent samples. Source data are provided in the Source Data file.

The human *C9ORF72* gene consists of 11 exons that are transcribed to three major transcript variants: V1, V2 and V3, with the repeat expansion located in intron 1 between exons 1a and 1b (Fig. 1a). These three transcript variants produce two protein isoforms: a 222-residue short isoform, known as C9-S, which is translated from V1, and a 481-residue long isoform, C9-L, translated from V2 and V3 (Fig. 1a)^10,41^.

Both V1 and V3 encode the repeat expansion, while transcription of V2, the predominant variant thought to account for ~85–95% of the C9ORF72 transcripts in the brain^25,42^, is initiated downstream of the repeat (Fig. 1a)^13^. Because the partial loss of the C9ORF72 protein has been hypothesized as a potential source of the pathogenicity of the repeat expansion^43,44^, we sought to use RfxCas13d to target either exon 1a or intron 1 to preferentially suppress V1 and V3 (Fig. 1a), a strategy we expected would spare V2, a major source of the C9-L protein.

To facilitate the design of crRNAs to target the repeat-containing RNA, we utilized the Cas13 Design Resource, an online tool that can predict active crRNAs for RfxCas13d^45,46^. Using this resource, we searched the exon 1a and the intron 1 sequences flanking the G_4_C_2_ repeat before selecting the 15 highest ranking crRNAs for detailed testing (Fig. 1a and Supplementary Fig. 1). We then created a dual-luciferase reporter system to determine the ability of the crRNAs to target their respective sequences. This platform consisted in part of a Renilla luciferase transgene whose 3′ untranslated region (UTR) was fused to 20 copies of the G_4_C_2_ repeat, the longest length that could be commercially synthesized, flanked by 250- and 98-base pairs (bps) of the 5′ and 3′ sequences of the *C9ORF72* gene, respectively (Fig. 1b). When complexed with a functional crRNA, RfxCas13d is thus expected to cleave the Renilla luciferase transcript, which in turn is anticipated to decrease its expression. Importantly, we observed no significant difference in the activity of the 3′ UTR-modified Renilla luciferase variant compared to its unmodified form (P > 0.05; Supplementary Fig. 2), indicating that: (i) modifying its 3′ UTR did not markedly affect the strength of its signal for this assay and (ii) this platform possessed sufficient activity to enable our screen. Notably, this strategy was adapted from a previous study, which fused a region of the C9ORF72 transcript to the 3′ UTR of Renilla to facilitate the identification of microRNAs against it^25^, thus providing a proof-of-concept validation for this approach. This reporter has since used by other studies to identify antisense oligonucleotides (ASOs) for C9ORF72^30,32^, providing further validation.

Recognition of a target RNA by RfxCas13d unlocks its intrinsic ability to indiscriminately *trans*-cleave non-specific bystander RNAs^47–51^, which in turn can lead to off-target effects. Given the potential for this, we incorporated into our screen a second reporter to monitor collateral effects (i.e., the degradation of non-target RNAs). As the extent of *trans*-cleavage can depend on the crRNA^52,53^, we reasoned that utilizing a dedicated reporter to assess *trans*-cleavage could facilitate the identification of crRNAs that minimize this effect and potentially induce fewer off-target effects across the transcriptome^50^. For this, we used firefly luciferase (Fig. 1c), which was not targeted by any crRNA but whose expression was nonetheless expected to decrease if collaterally *trans*-cleaved by RfxCas13d.

To test the crRNAs, we co-transfected human embryonic kidney (HEK) 293 T cells with each reporter plasmid and an expression vector encoding a RfxCas13d variant with two NLS sequences, one attached to the N-terminus and the other to the C-terminus, alongside one of the 15 repeat-targeting crRNAs. From this screen, we found that all 15 crRNAs decreased Renilla luciferase by >75% at 72 h post-transfection (P < 0.0001) and that 14 of the 15 crRNAs decreased it by at least 95% (P < 0.0001; Fig. 1d), indicating that each crRNA effectively targeted Renilla luciferase. However, we found that the majority of the crRNAs also triggered a reduction in firefly luciferase (Fig. 1d), suggesting they exerted collateral effects. This notwithstanding, we identified two crRNAs, crRNAs 13 and 1, that decreased Renilla luciferase by ~95% (P < 0.0001) and had no significant effect on firefly luciferase compared to the control (P > 0.05; Fig. 1d), thus indicating they engaged with their target and exerted no detectable bystander effects.

In addition to Cas13, Cas7-11, a subtype III-E effector formed from the fusion of a putative Cas11 domain with multiple Cas7 subunits, can mediate RNA knockdown in mammalian cells but with reportedly limited collateral effects^54^. Given these properties, we also determined its ability to target C9ORF72. For this purpose, we co-transfected HEK293T cells with the above-described dual-reporter system and an expression vector encoding the Cas7-11 protein from *Desulfonema ishimotonii* (DiCas7-11) alongside one of 15 crRNAs designed to target the equivalent positions in C9ORF72 as the crRNAs for RfxCas13d (Supplementary Fig. 1 and Supplementary Fig. 3a). In total, only eight crRNAs for DiCas7-11 significantly decreased Renilla expression (P < 0.05; Supplementary Fig. 3b), with the most effective of these, crRNA-2, found to decrease it by only ~45% (P < 0.001; Supplementary Fig. 3b). Surprisingly, eleven of the fifteen crRNAs also decreased firefly luciferase by at least 20% relative to the non-targeting control (P < 0.05 for all eleven; Supplementary Fig. 3b), implying non-specific activity. Thus, due to its improved ability to target C9ORF72 based on our dual-reporter screen, we utilized RfxCas13d for our studies.

Following our dual-reporter screen, we determined if RfxCas13d could target C9ORF72 mRNA in HEK293T cells. Specifically, we evaluated the targeting capabilities of three crRNAs: crRNAs 13, 7, and 1. Among the five crRNAs that reduced firefly luciferase by less than two-fold (Fig. 1d), crRNAs 13, 7 and 1 had the most favorable targeting scores, a number we defined as the ratio of Renilla (i.e., target) to firefly (i.e., non-target) luciferase (Supplementary Fig. 4). To quantify targeting, validated qPCR probes were used for: (i) V3^32^, which served as the proxy for the G_4_C_2_ repeat-containing RNA in this study, and (ii) the all-variant (all-V) pool, which consists predominately of V2^25,42,55^ and thus was not expected to be perturbed by our approach.

Compared to cells transfected with RfxCas13d and a non-targeted crRNA, we measured by qPCR that each of the three repeat-targeting crRNAs decreased the relative abundance of V3 mRNA by ~90–95% (P = 0.001 for crRNAs 1 and 7; P < 0.001 for crRNA 13; Fig. 1e). Importantly, relative to the same controls, we measured that these crRNAs either had no effect on all-V (crRNAs 13 and 1; P > 0.05) or a limited effect on it (crRNA-7; Fig. 1e), indicating the ability for RfxCas13d to preferentially target V3.

As an additional control, we tested in HEK293T cells two previously validated ASOs for C9ORF72: ASO-2, which binds intron 1 and selectively targets V1 and V3^27^, and ASO-4, which binds exon 2 and thus targets the three main transcript variants^27^. As expected, qPCR revealed that ASO-2 lowered only V3 (P < 0.01) and that ASO-4 decreased both V3 and all-V (P < 0.01 for both; Fig. 1e), reinforcing the validity of our measurement methods.

We next evaluated if RfxCas13d could target V3 in a second human cell line: SH-SY5Y neuroblastoma cells. Compared to cells transfected with RfxCas13d and a non-targeting crRNA, we found by qPCR that crRNAs 13, 7 and 1 decreased V3 mRNA by 80-95% (P < 0.001 for all; Fig. 1f) and that each of the three crRNAs had no significant effect on all-V (P > 0.05 for all; Fig. 1f). Consistent with our prior control measurements, ASO-2 was found to only lower V3 in SH-SY5Y cells (P < 0.01), while ASO-4 decreased both V3 and all-V (P < 0.01 for V3 and P < 0.001 for all-V; Fig. 1f).

In sum, we find that a dual-luciferase reporter screen designed to consider both target engagement and collateral effects can be used to identify crRNAs for C9ORF72, and that RfxCas13d can preferentially target V3, a C9ORF72 transcript variant that carries the hexanucleotide repeat.

### RfxCas13d can decrease the G4C2 repeat RNA and reduce RNA foci formation in a C9-ALS/FTD mouse model

We next asked whether RfxCas13d could target the G_4_C_2_ repeat RNA in a mouse model of C9-ALS/FTD, specifically C9-BACexp mice^56^, which harbor a bacterial artificial chromosome encoding the full-length human *C9ORF72* gene with ~100–1000 copies of the hexanucleotide repeat. Similar to other rodent models of C9-ALS/FTD^57^, C9-BACexp mice do not manifest an overt motor phenotype.

The motor cortex (MC) and hippocampus (HPC) are two structures in C9-BACexp mice that form RNA foci comprised of the G_4_C_2_ repeat RNA and produce measurable quantities of the DPR protein poly(GP)^56^. To target RfxCas13d to these areas, we chose to locally deliver it using the engineered AAV vector variant PHP.eB^58^, reasoning that its delivery via an intracranial injection would enable a straightforward analysis of its outcomes. Though most commonly administered intravenously, PHP.eB can transduce cells to a similar extent as AAV9 when injected locally to the brain^59^ but, in the experience of our laboratory, packages at a higher titer, which can decrease manufacturing burden.

**Fig. 2:**
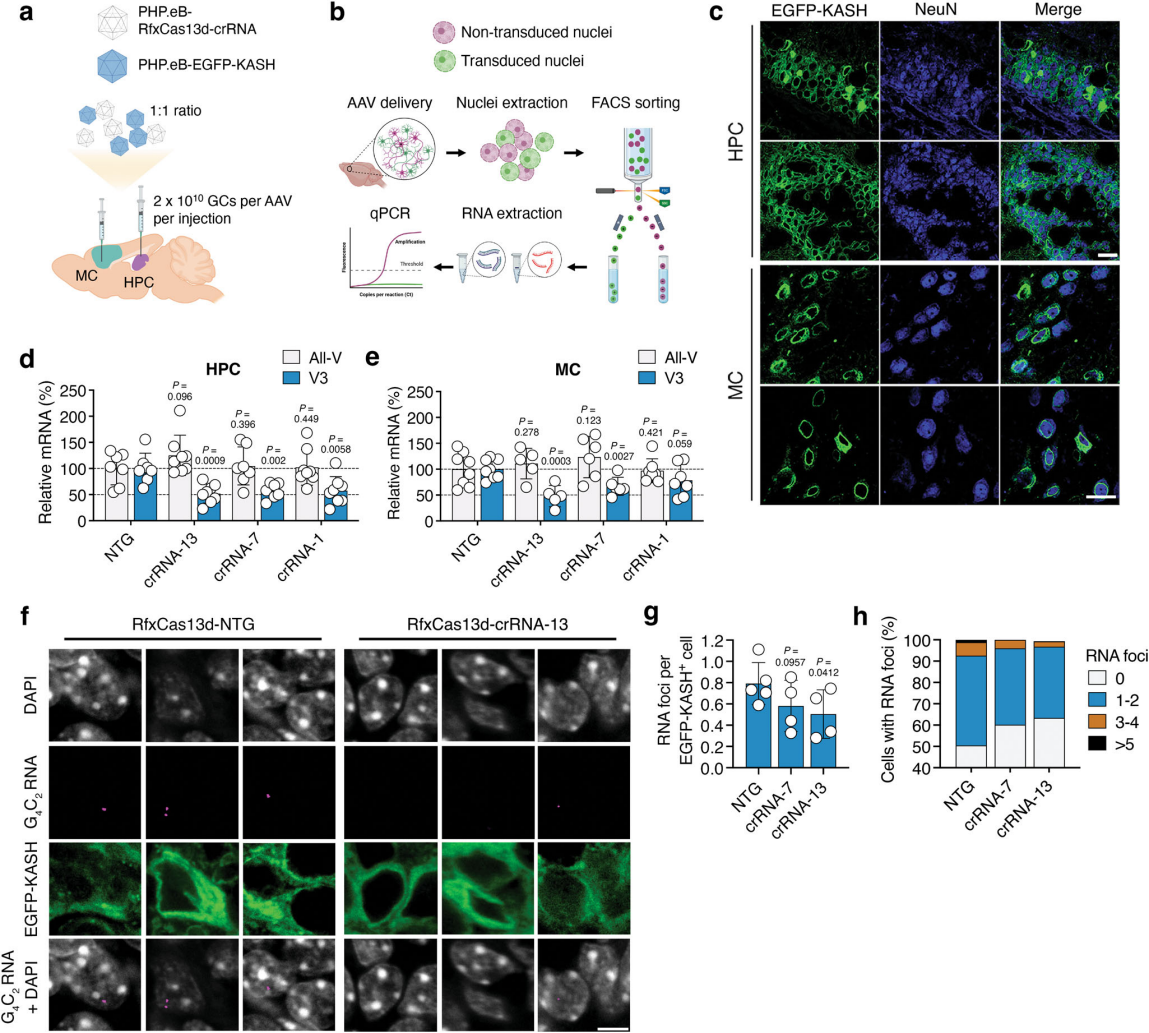
RfxCas13d can target the G_4_C_2_ repeat-containing RNA and reduce RNA foci in C9-BACexp mice. **a** Cartoon of the injection scheme. Created in BioRender. Gaj, T. (2024) https://BioRender.com/a93i238. **b** The experimental plan to analyze C9ORF72 mRNA in EGFP-KASH^+^ nuclei isolated by fluorescence-activated cell sorting (FACS). Created in BioRender. Gaj, T. (2024) https://BioRender.com/w54s355. **c** Representative immunofluorescent staining of the hippocampus (HPC) and motor cortex (MC) in C9-BACexp mice two-months after injection with 2 × 10^10^ GCs each of PHP.eB-RfxCas13d-crRNA and PHP.eB-EGFP-KASH. Scale bar, 20 µm. Relative all-V and V3 mRNA in **d** the HPC and **e** MC of EGFP-KASH^+^ nuclei from C9-BACexp mice injected with 2 × 10^10^ GCs each of PHP.eB-RfxCas13d-crRNA and PHP.eB-EGFP-KASH. All-V and V3 for each crRNA normalized to the non-targeted (NTG) control (*n* ≥ 7). **f** Representative RNA FISH for the G_4_C_2_ repeat RNA (magenta) from the HPC of C9-BACexp mice injected with 2 × 10^10^ GCs each of PHP.eB-RfxCas13d-crRNA and PHP.eB-EGFP-KASH. Scale bar, 5 µm. Quantification of **g** the number of RNA foci per EGFP-KASH^+^ cell in the HPC and **h** the percentage of EGFP-KASH^+^ cells with 0, 1–2, 3–4, or >5 foci (*n* ≥ 4). **g**, **h** The total number of cells counted per biological replicate is described in Supplementary Table 1. RNA foci were counted by a blinded investigator. Values indicate means and error bars indicate SD. **d**, **e** All-V and V3 for each crRNA compared to NTG using a one-tailed unpaired t-test, with exact P values shown. **g** crRNAs 13 and 7 compared to NTG using a one-tailed unpaired t-test, with exact P values shown. All data points are biologically independent samples. Source data are provided in the Source Data file.

The HPC and MC of two-month-old C9-BACexp mice were thus injected with 2 × 10^10^ genome copies (GCs) of a PHP.eB vector carrying a CAG-driven RfxCas13d variant alongside either crRNAs 13, 7 or 1 (i.e., PHP.eB-RfxCas13d-crRNA-13, -7 or -1) or a non-targeting crRNA (i.e., PHP.eB-RfxCas13d-NTG; Fig. 2a). To facilitate the isolation of transduced nuclei for a more detailed analysis of RfxCas13d-mediated outcomes, we also co-injected C9-BACexp mice with 2 × 10^10^ GCs of a second PHP.eB vector encoding a CAG-driven EGFP variant fused to KASH (Klarsicht/ANC-1/Syne-1 homology; PHP.eB-EGFP-KASH), a domain that promotes EGFP localization to the outer nuclear membrane^60^, which can enable the isolation of transduced nuclei by fluorescence-activated cell sorting (FACS)^61,62^ (Fig. 2a, b). As its presence in the RfxCas13d-encoding AAV vector would cause it to exceed its carrying capacity, EGFP-KASH was delivered using a second AAV. However, because a proportion of cells can be transduced by multiple AAV vector genomes^58,63^, dual-vector-based approaches such as this one can be used to enrich for nuclei transduced by the first vector^61,64,65^.

Based on an immunofluorescent analysis conducted at two-months post-injection, we observed strong EGFP-KASH expression at the sites of injection in C9-BACexp mice treated with each AAV formulation. Within the HPC and MC, we determined that ~91% and ~53% of the cells positive for the pan-neuronal marker NeuN, respectively, were positive for EGFP-KASH (Fig. 2c and Supplementary Fig. 5) and that ~72% and ~75% of the EGFP-KASH^+^ cells in the HPC and MC, respectively, were positive for RfxCas13d by its hemagglutinin (HA) epitope (Supplementary Fig. 6). Despite relying on the ubiquitous CAG promoter to drive its expression, EGFP-KASH was largely confined to NeuN^+^ cells, as we observed limited expression in GFAP^+^ astrocytes and Iba1^+^ microglia in both the HPC and MC (Supplementary Fig. 7).

We next used FACS to isolate EGFP-KASH^+^ nuclei from the HPC and MC of injected C9-BACexp mice to determine the abundance of V3 and all-V mRNA by qPCR (Fig. 2b). Relative to the EGFP-KASH^+^ cells enriched from mice injected with PHP.eB-RfxCas13d-NTG, each repeat-targeting crRNA was found to effectively decrease V3 (Fig. 2d, e), which served as our proxy for the G_4_C_2_ repeat RNA. The most potent and consistent crRNA, the exon 1a-targeting crRNA-13, was found to suppress V3 by ~48% in the HPC (P < 0.001; Fig. 2d) and ~52% in the MC (P < 0.001; Fig. 2e). Critically, all three repeat-targeting crRNAs were found to have no effect on all-V (P > 0.05 for the HPC and MC; Fig. 2d, e), indicating that RfxCas13d preferentially targeted V3 in vivo.

Using fluorescence in situ hybridization (FISH), we next determined whether targeting the repeat RNA with RfxCas13d affected RNA foci formation in C9-BACexp mice, specifically in the HPC, where they develop in 40–60% of cells^56^. Given their improved targeting capabilities, we quantified RNA foci in EGFP-KASH^+^ cells only for mice treated with RfxCas13d and crRNAs 13 and 7.

Compared to the non-targeted crRNA, mice injected with PHP.eB-RfxCas13d-crRNA-13 and PHP.eB-RfxCas13d-crRNA-7 had a ~37% and a ~31% decrease, respectively, in the number of foci positive for the G_4_C_2_ repeat RNA per EGFP-KASH^+^ cell (P < 0.05 for crRNA 13; P = 0.09 for crRNA 7; Fig. 2f, g and Supplementary Table 1). Further, we measured a shift in their distribution, with mice treated by each crRNA found to have an 11–13% increase in the number of EGFP-KASH^+^ cells with no detectable foci and a two-fold decrease in EGFP-KASH^+^ cells with 3–4 foci compared to the non-targeted crRNA (Fig. 2h). As an additional control, we quantified RNA foci in wild-type C57BL/6J mice injected with PHP.eB-EGFP-KASH, measuring only ~0.08 foci per EGFP-KASH^+^ cell in the HPC (Supplementary Fig. 8 and Supplementary Table 2), a ~10-fold difference compared to C9-BACexp mice, which is consistent with previous reports^56^.

Thus, we find that RfxCas13d can be delivered to C9-BACexp mice, where it can suppress the G_4_C_2_ repeat RNA and affect the formation of RNA foci in neurons.

### High-fidelity RfxCas13d can target C9ORF72 with improved transcriptome-wide specificity

**Fig. 3:**
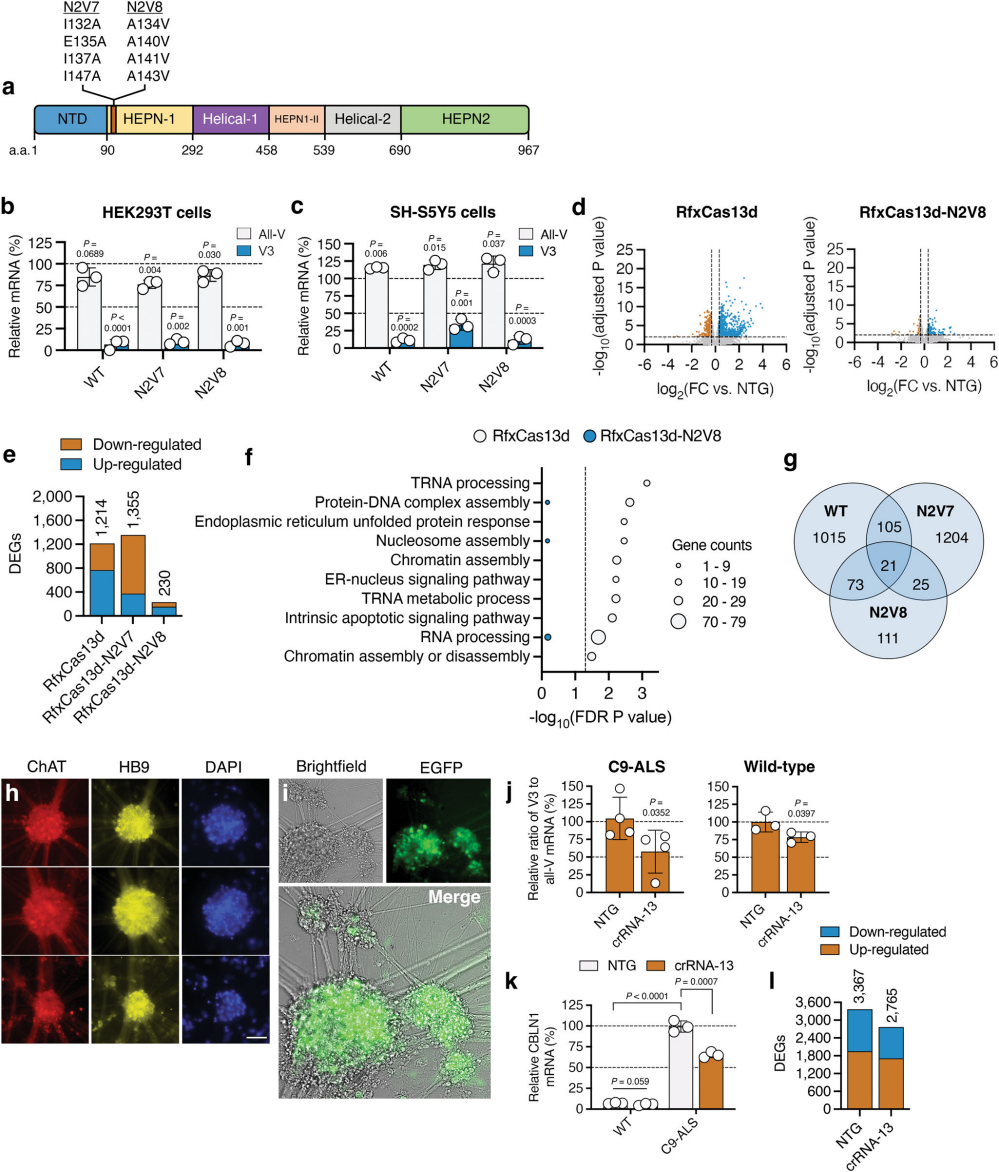
High-fidelity RfxCas13d has improved specificity and can mediate targeting in cells derived from an ALS patient. **a** RfxCas13d domain organization with RfxCas13d-N2V7 and RfxCas13d-N2V8 mutations shown. Relative all-V and V3 mRNA in **b** HEK293T and **c** SH-SY5Y cells transfected with RfxCas13d, RfxCas13d-N2V7 and RfxCas13d-N2V8 with crRNA-13. All values normalized to untreated cells (*n* = 3). **d** Volcano plot of the RNA-seq analysis comparing HEK293T cells transfected with (left) RfxCas13d or (right) RfxCas13d-N2V8 with crRNA-13 to each variant with a NTG crRNA (*n* = 3). Lines denote a > 1.25-fold change (FC) and an FDR-adjusted P < 0.01. **e** Number of differentially expressed genes (DEGs) [>1.25-FC, FDR-adjusted P < 0.01] from (**d**). **f** Gene ontology (GO) and biological process (BP) term analysis for the DEGs in (**d**). Line denotes FDR-adjusted P < 0.05. **g** Venn diagram of overlapping DEGs. **h** Immunostaining of C9-ALS neurospheres. Scale bar, 75 µm. **i** Brightfield and fluorescent images of C9-ALS neurospheres 14 days after treatment with PHP.eB-EGFP-KASH. **h**, **i** Immunostaining and visualization were conducted once. Relative **j** V3 to all-V mRNA ratio and **k** CBLN1 mRNA in C9-ALS or wild-type neurospheres treated with PHP.eB-RfxCas13d-N2V8-crRNA (*n* ≥ 3). **j** Values normalized to NTG crRNA. **k** Values normalized to NTG in C9-ALS cells. **l** Number of DEGs [>1.25-FC from wild-type cells, FDR-adjusted P < 0.01] in C9-ALS cells treated with PHP.eB-RfxCas13d-N2V8-crRNA. Values indicate means and error bars indicate SD. **b**, **c** All-V and V3 for each RfxCas13d variant compared to untreated cells using a two-tailed unpaired t-test, with exact P values shown. **d**–**f** FDR-adjusted P values were determined by a global FDR correction across pairwise comparisons. **j**, **k** crRNA-13 compared to NTG using a one-tailed unpaired t-test, with exact P values shown. **k** NTG for C9-ALS iMNs compared to NTG for WT iMNs using a one-tailed unpaired t-test, with the exact P value shown. **l** FDR-adjusted P values were determined by a global FDR correction across pairwise comparisons. All data points are biologically independent samples. Source data are provided in the Source Data file.

Because of its risk for inducing collateral effects^49,50^, high-fidelity forms of Cas13 that possess a reduced capacity to *trans*-cleave non-target RNAs have been developed^53^. To determine their potential for targeting C9ORF72, we transfected HEK293T cells with an expression vector encoding the native RfxCas13d protein or one of two high-fidelity variants^53^, RfxCas13d-N2V7 and RfxCas13d-N2V8 (Fig. 3a) with either crRNA 13, the top-performing repeat-targeting crRNA from our first study, or a non-targeted crRNA. Both RfxCas13d-N2V7 and RfxCas13d-N2V8 carry mutations in their HEPN1 domain that are thought to reduce non-specific RNA cleavage^53^.

Based on qPCR, both high-fidelity RfxCas13d variants decreased V3 mRNA in HEK293T cells (P < 0.01 for both; Fig. 3b), with RfxCas13d-N2V8 found to reduce its expression by >90% compared to untransfected cells, an effect on par with the native protein (Fig. 3b). Similar to the native enzyme, RfxCas13d-N2V8 was also found to have a minimal effect on all-V in HEK293T cells (Fig. 3b).

We further tested RfxCas13d-N2V8 and RfxCas13d-N2V7 in SH-SY5Y cells, where we observed by qPCR that both variants decreased V3 mRNA (P < 0.01 for both; Fig. 3c) without decreasing all-V (Fig. 3c), though RfxCas13d-N2V8 was found to target V3 more efficiently (~89% decrease) than RfxCas13d-N2V7 (~63% decrease).

We next determined if RfxCas13d or its high-fidelity counterparts induced toxicity. Using propidium iodide to measure viability, we observed a modest but nonetheless measurable increase in the number of non-viable HEK293T and SH-SY5Y cells following their transfection with the native RfxCas13d protein and crRNA-13 (P < 0.05 for both; Supplementary Fig. 9). However, no change in viability was observed in HEK293T cells or SH-SY5Y cells following transfection with RfxCas13d-N2V8 or RfxCas13d-N2V7 (P > 0.05 for all; Supplementary Fig. 9).

To further interrogate the effect of targeting V3 by RfxCas13d, we used western blot to measure C9-L, the full-length C9ORF72 protein isoform. Because our approach preferentially targets V3 and because C9-L is encoded by V2 (Fig. 1a), we expected that RfxCas13d and its high-fidelity counterparts would have no effect, or a minimal effect, on the relative abundance of C9-L. Consistent with this reasoning, no difference in the C9-L protein was measured in HEK293T cells transfected with any RfxCas13d variant with crRNA-13 relative to their respective control (P > 0.05 for all; Supplementary Fig. 10). C9-S, the short C9ORF72 protein isoform encoded by V1, could not be detected, and thus was not analyzed.

We next used RNA-seq to compare the transcriptome-wide specificities of RfxCas13d and its high-fidelity counterparts. When programmed with crRNA-13, the native RfxCas13d protein perturbed the expression of 1214 genes in HEK293T cells at 72 h post-transfection (>1.25-fold change [FC]; false discovery rate [FDR]-adjusted P < 0.05), while RfxCas13d-N2V8 affected only 230 genes at the same time-point, a ~5.8-fold decrease (Fig. 3d, e and Supplementary Data 1 and 2). Interestingly, we found that RfxCas13d-N2V7 perturbed a similar number of genes (1355) as the native protein (Fig. 3e and Supplementary Data 1 and 2), indicating it possessed decreased transcriptome-wide specificity relative to RfxCas13d-N2V8 when paired with crRNA-13.

Through an over-representation analysis of gene ontology (GO) and biological process (BP) terms, we next compared the themes enriched for the genes affected by the native RfxCas13d protein and RfxCas13d-N2V8. For RfxCas13d, we observed enrichment (FDR-adjusted P < 0.05) for ten biological functions (Fig. 3f and Supplementary Data 3), including RNA processing, protein-DNA complex assembly, intrinsic apoptotic signaling and chromatin assembly and disassembly, several of which have been linked to RfxCas13d and its collateral effects^53^. However, no enrichment (FDR-adjusted P > 0.05) was observed for these or any other themes in cells transfected with RfxCas13d-N2V8 (Fig. 3f and Supplementary Data 4).

As expected, given our approach spares V2, the predominant C9ORF72 transcript variant, we observed no enrichment for function(s) related to the C9ORF72 protein for any RfxCas13d variant. Interestingly, unlike for RfxCas13d-N2V7, the majority (68%) of the genes affected by RfxCas13d-N2V8 were up-regulated (Fig. 3e); however, no themes (FDR-adjusted P > 0.05) were identified for these differentially expressed genes (DEGs).

Last for this analysis, we analyzed the shared DEGs between the native RfxCas13d protein, RfxCas13d-N2V8 and RfxCas13d-N2V7. In total, only 21 DEGs were shared among all three variants, though 94 DEGs were shared between RfxCas13d and RfxCas13d-N2V8 (Fig. 3g and Supplementary Data 2), with a GO and BP term analysis found to reveal an enrichment in functions related to the positive regulation of cellular component organization and epithelial cell differentiation (Supplementary Data 5).

Given its improved transcriptome-wide specificity compared to its native form and RfxCas13d-N2V7, we next determined the ability of RfxCas13d-N2V8 to target V3 in a more physiologically relevant cell line: iPSC-derived motor neuron-like cells from a 64-year-old female ALS patient with >145 copies of the G_4_C_2_ repeat in the *C9ORF72* gene.

Starting with neural progenitor cells from this ALS patient, as well as a non-ALS female donor, we conducted a two-week differentiation protocol that produced HB9^+^ and ChAT^+^ neurospheres (Fig. 3h) that we then analyzed by RNA-seq. In total, 2,470 DEGs were identified in the C9-ALS neurospheres following a pairwise comparison to the non-ALS donor, hereafter referred to as wild-type (>1.25 FC; FDR P < 0.01; Supplementary Fig. 11a and Supplementary Data 6 and 7), with a GO and BP term analysis found to reveal enrichment for several functions previously found to be affected in induced motor neurons (iMNs) from a C9-ALS patient, including synapse organization and neurotransmitter transport^66^ (Supplementary Fig. 11b and Supplementary Data 8).

After 14 days in vitro, the C9-ALS and wild-type neurospheres were treated with PHP.eB vector encoding a CAG-driven RfxCas13d-N2V8 variant with crRNA-13 (PHP.eB-RfxCas13d-N2V8-crRNA-13) or a non-targeted crRNA (PHP.eB-RfxCas13d-N2V8-NTG) at a multiplicity of infection (MOI) of ~2 × 10^6^. To visualize transduction, neurospheres were also separately treated with a PHP.eB vector encoding a CAG-driven EGFP variant at the same MOI.

After determining qualitatively that EGFP was expressed in the neuron-like clusters at 14 days post-transduction (Fig. 3i), we used qPCR to determine the relative ratio of V3 to all-V for each sample, finding that C9-ALS neurospheres treated with PHP.eB-RfxCas13d-N2V8-crRNA-13 had a ~40% decrease in relative V3 mRNA compared to cells treated with the non-targeting crRNA (P < 0.05; Fig. 3j). This ratio was also decreased by ~22% in wild-type cells treated with PHP.eB-RfxCas13d-N2V8-crRNA-13 (P < 0.05; Fig. 3j). Because crRNA-13 is not selective for the hexanucleotide repeat expansion and can target the wild-type V3 transcript, these data demonstrate targeting in a second iPSC-based cell line, which helps support the reproducibility of this approach within this model.

In addition to the G_4_C_2_ repeat RNA, qPCR was also used to measure the antisense RNA, which revealed no change in its expression in either the C9-ALS neurospheres or the wild-type neurospheres following their treatment with PHP.eB-RfxCas13d-N2V8-crRNA-13 (P > 0.05 for both; Supplementary Fig. 12).

RNA-seq was next used to determine the transcriptome-wide specificity of RfxCas13d-N2V8 in the wild-type neurospheres at 14 days post-transduction. When paired with crRNA-13, RfxCas13d-N2V8 was found to perturb the expression of only two genes (BMP3 and COL6A5; >1.25-FC to non-targeted crRNA; FDR-adjusted P < 0.01; Supplementary Data 9 and 10), both of which were down-regulated. Interestingly, BMP3 and COL6A5 are both involved in cartilage signaling, a finding that could benefit from further study, though we note that such an analysis is outside the scope of the current work.

We next asked if RfxCas13d-N2V8 could reverse, or partially reverse, a disease-associated transcriptional alteration in the C9-ALS neurospheres. To answer this, we used qPCR to measure the expression of CBLN1, a member of the cerebellin family of proteins that normally contributes to the formation and function of synapses. CBLN1 has been found to be up-regulated in neuron-like cells derived from C9-ALS patients^67,68^, a finding we corroborated by qPCR, which showed a ~14-fold increase in its expression in the C9-ALS iMNs versus the wild-type cells (P < 0.0001; Fig. 3k). At 14 days post-transduction, however, we found that C9-ALS neurospheres treated with RfxCas13d-N2V8 had a ~35% decrease in CBLN1 mRNA compared to cells treated with the non-targeted crRNA (P < 0.001; Fig. 3k), with no change in its expression observed in the wild-type cells treated with PHP.eB-RfxCas13d-N2V8-crRNA-13 (P > 0.05; Fig. 3k).

Finally, RNA-seq was used to determine the transcriptome-wide effects of RfxCas13d-N2V8 in the C9-ALS neurospheres. At 14 days post-transduction, we found that C9-ALS cells treated with PHP.eB-RfxCas13d-N2V8-crRNA-13 had ~18% fewer DEGs than cells treated with the non-targeted crRNA (>1.25-FC to wild-type iMNs; FDR-adjusted P < 0.01; Fig. 3l and Supplementary Data 11). For the DEGs whose expression was normalized, a term analysis revealed enrichment for functions related to RNA metabolism, including RNA splicing and mRNA splicing, the regulation of RNA and mRNA splicing, and the regulation of mRNA processing (Supplementary Fig. 13 and Supplementary Data 12 and 13).

Altogether, our results demonstrate that a high-fidelity form of RfxCas13d can be used to target V3 and that the most effective of these variants, RfxCas13d-N2V8, possesses improved transcriptome-wide specificity compared to the native enzyme. We also find that this high-fidelity variant can reduce the relative abundance of the G_4_C_2_ repeat RNA in neuron-like cells derived from a C9-ALS patient.

### High-fidelity RfxCas13d can target the G4C2 repeat RNA and improve abnormalities in a C9-ALS/FTD mouse model

**Fig. 4:**
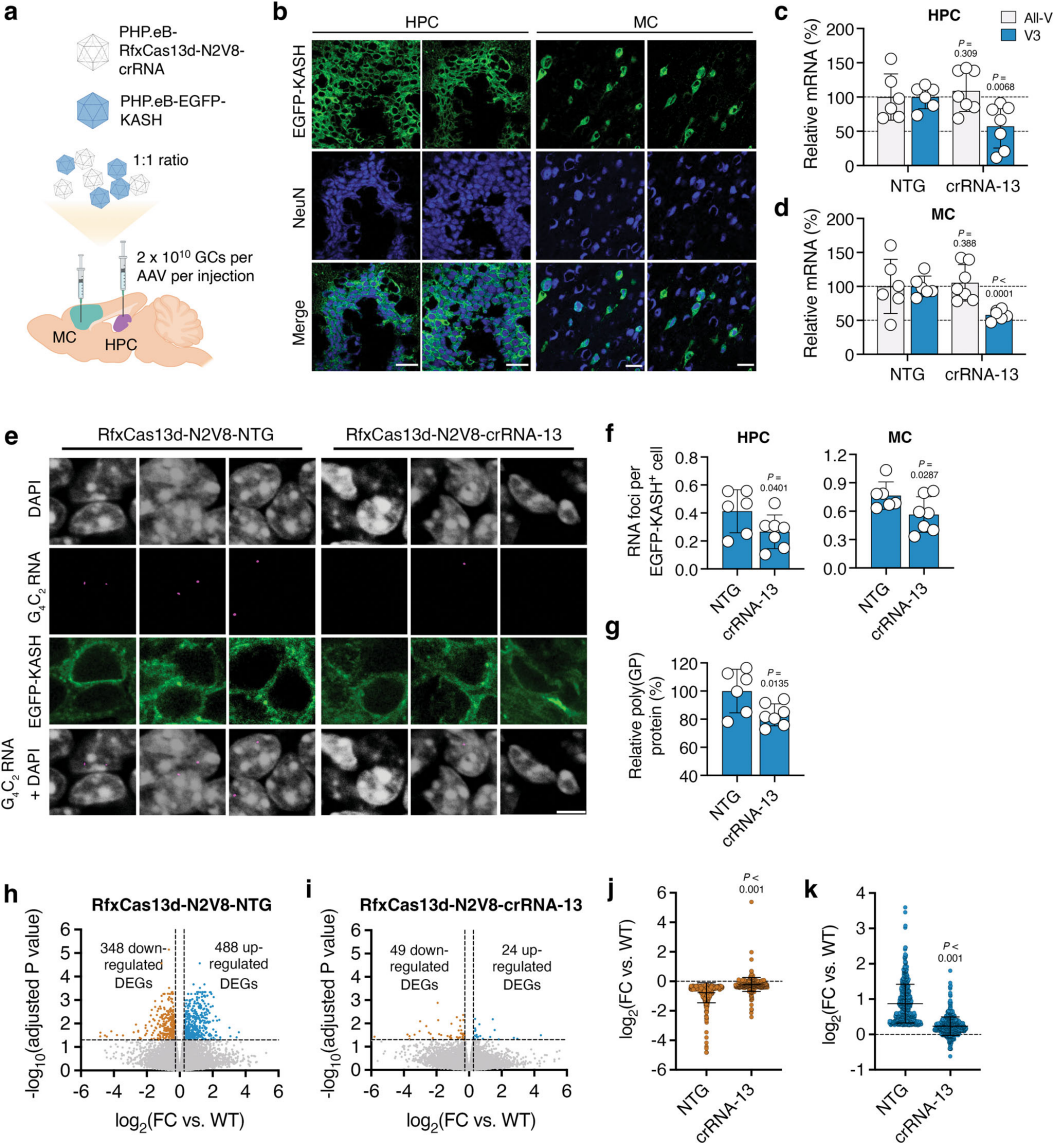
High-fidelity RfxCas13d can target the G_4_C_2_ repeat-containing RNA and improve deficits in C9-BACexp mice. **a** Cartoon of the injection scheme. Created in BioRender. Gaj, T. (2024) https://BioRender.com/a93i238. **b** Representative immunofluorescent staining of the hippocampus (HPC) and motor cortex (MC) in C9-BACexp mice two-months after injection with 2 × 10^10^ GCs each of PHP.eB-RfxCas13d-N2V8-crRNA and PHP.eB-EGFP-KASH. Scale bar, 30 µm. Relative all-V and V3 mRNA in EGFP-KASH^+^ nuclei from **c** the HPC and **d** MC of C9-BACexp mice injected with 2 × 10^10^ GCs each of PHP.eB-RfxCas13d-N2V8-crRNA with PHP.eB-EGFP-KASH. All-V and V3 for crRNA-13 normalized to the non-targeted (NTG) control (*n* ≥ 6). **e** Representative FISH for the G_4_C_2_ repeat RNA (magenta) in EGFP-KASH^+^ cells from the HPC of C9-BACexp mice injected with 2 × 10^10^ GCs each of PHP.eB-RfxCas13d-N2V8-crRNA and PHP.eB-EGFP-KASH. Scale bar, 5 µm. **f** Quantification of the number of RNA foci per EGFP-KASH^+^ cell in the (left) HPC and (right) MC of injected C9-BACexp mice (*n* ≥ 6). The total number of cells counted per biological replicate is described in Supplementary Table 3. **g** Soluble poly(GP) in the HPC of injected C9-BACexp mice (*n* ≥ 6). Volcano plot of the RNA-seq analysis from C9-BACexp mice injected with **h** PHP.eB-RfxCas13d-N2V8-NTG or **i** PHP.eB-RfxCas13d-N2V8-crRNA-13 and compared to wild-type littermates (*n* = 3–7). Lines denote a > 1.2-fold change (FC) and an FDR-adjusted P < 0.05. FC of the (**j**) down-regulated  or (**k**) up-regulated  DEGs from the analysis in (**h**, **i**). Values indicate means and error bars indicate SD. **c**, **d** All-V and V3 for crRNA-13 compared to NTG using a one-tailed unpaired t-test, with exact P values shown. **f**, **g** crRNA−13 compared to NTG using a one-tailed unpaired t-test, with exact P values shown. **h**, **i** FDR-adjusted P values were determined by a global FDR correction across pairwise comparisons. **j**, **k** crRNA-13 compared to NTG using a two-tailed unpaired t-test. All data points are biologically independent samples. Source data are provided in the Source Data file.

We next determined if RfxCas13d-N2V8 could target the G_4_C_2_ repeat RNA in vivo. Mirroring our earlier study, we injected the HPC and MC of two-month-old C9-BACexp mice with 2 × 10^10^ GCs of PHP.eB-RfxCas13d-N2V8-crRNA-13 or PHP.eB-RfxCas13d-N2V8-NTG. To facilitate the isolation of transduced nuclei for a higher-resolution analysis of RfxCas13d-N2V8-mediated outcomes, we also co-injected each site with 2 × 10^10^ GCs of PHP.eB-EGFP-KASH (Fig. 4a).

Consistent with our earlier study, we observed robust EGFP-KASH expression in C9-BACexp mice, finding at two-months post-injection that ~87% and ~49% of the cells positive for NeuN in the HPC and MC, respectively, were positive for EGFP-KASH (Fig. 4b and Supplementary Fig. 14) and that ~81% and ~78% of the EGFP-KASH^+^ cells in the HPC and MC, respectively, were positive for RfxCas13d-N2V8 by its HA epitope (Supplementary Fig. 15). As before, we observed limited delivery to glial cells, which included GFAP^+^ astrocytes and Iba1^+^ microglia (Supplementary Fig. 16).

Following their isolation by FACS, qPCR was used to measure V3 and all-V in EGFP-KASH^+^ nuclei from the HPC and MC of injected C9-BACexp mice. Relative to control animals, C9-BACexp mice injected with PHP.eB-RfxCas13d-N2V8-crRNA-13 were found to have a two-fold decrease in V3, both in the HPC and the MC (P < 0.01 for the HPC and P < 0.0001 for the MC; Fig. 4c, d), and showed no difference in all-V in either region (P > 0.1 for both the HPC and MC; Fig. 4c, d). Via qPCR, we also measured the antisense RNA in the EGFP-KASH^+^ cell populations, observing no change in its expression in mice treated with PHP.eB-RfxCas13d-N2V8-crRNA-13 (P > 0.05 for the HPC and MC; Supplementary Fig. 17), indicating that suppressing the G_4_C_2_ repeat RNA with crRNA-13 did not affect the antisense transcript in vivo.

Using FISH, we next determined if RfxCas13d-N2V8 affected RNA foci formation in C9-BACexp mice. Within the HPC, we measured a ~37% decrease in foci positive for the G_4_C_2_ repeat RNA in animals treated by RfxCas13d-N2V8 (P < 0.05; Fig. 4e, f and Supplementary Table 3), which coincided with a shift in their distribution (Supplementary Fig. 18a). We also quantified RNA foci formation in the MC, measuring a ~27% decrease in the number of foci per cell (P < 0.05; Fig. 4f), alongside a similar shift in their distribution (Supplementary Fig. 18b).

We next evaluated if RfxCas13d-N2V8 decreased poly(GP), a DPR protein translated from the G_4_C_2_ repeat RNA and which is produced in C9-BACexp mice^56^. Using a Meso Scale Discovery immunoassay platform^14,32,69,70^, we measured an ~18% decrease in soluble poly(GP) in bulk hippocampal tissue from mice injected with PHP.eB-RfxCas13d-N2V8-crRNA-13 (P < 0.05; Fig. 4g). An ~8% decrease in soluble poly(GP) was also measured in bulk cortical tissue (Supplementary Fig. 19), though this result did not meet the threshold for statistical significance for our study (P > 0.05). We attribute this weaker effect to the more limited transduction observed in the MC, where only ~49% of the NeuN^+^ cells in the injected area were found to be positive for EGFP-KASH versus the HPC, where ~87% of the NeuN^+^ cells in the injected area were EGFP-KASH^+^ (Supplementary Fig. 14), a nearly two-fold difference. This is particularly meaningful given that the bulk tissue used for these analyses consists of a mixture of transduced and non-transduced cells, with the presence of the latter expected to dampen the measured effect. This is in contrast to: (i) our analysis of V3 mRNA, which was conducted on RNA isolated from EGFP-KASH^+^ cell populations enriched by FACS, and (ii) our quantification of RNA foci, which was limited to EGFP-KASH^+^ cells.

To determine whether RfxCas13d-N2V8 targeting induced adverse effects, we measured body weight, motor coordination, and hindlimb and forelimb grip strength in C9-BACexp mice injected with PHP.eB-RfxCas13d-N2V8-crRNA-13 or PHP.eB-RfxCas13d-N2V8-NTG. Over a two-month period, no discernable deficits were observed in mice treated with the repeat-targeting crRNA versus the non-targeted control (P > 0.05 for all measurements; Supplementary Fig. 20).

Following these behavioral assessments, we determined the distribution of the RfxCas13d-N2V8-encoding AAV vector. Based on qPCR, we measured ~114 and ~152 vector genomes per diploid genome (vg/dg) in the HPC of C9-BACexp mice injected with PHP.eB-RfxCas13d-N2V8-crRNA-13 and PHP.eB-RfxCas13d-N2V8-NTG, respectively, and ~25 and ~22 vg/dg in the MC (Supplementary Fig. 21). On average, between ~1.1 and ~2.6 vg/dg were measured in the heart, lungs, liver, kidney, and spleen of these C9-BACexp mice (Supplementary Fig. 21), with qPCR found to reveal no change in V3, all-V or the antisense RNA in any of these peripheral tissues (P > 0.05 for all; Supplementary Fig. 22).

Finally, we evaluated if RfxCas13d-N2V8 could reverse the transcriptional abnormalities that manifest in C9-BACexp mice^34^. To determine this, we injected the MC of six-week-old C9-BACexp mice and their age-matched C57BL/6 J littermates with 2 × 10^10^ GCs of PHP.eB-RfxCas13d-N2V8-crRNA-13 or PHP.eB-RfxCas13d-N2V8-NTG alongside 2 × 10^10^ GCs of PHP.eB-EGFP-KASH, which we used to facilitate the isolation of transduced nuclei by FACS at two-months post-injection. We then conducted RNA-seq on the EGFP-KASH^+^ nuclei isolated from each animal.

To uncover the gene alterations that could be attributed to the repeat expansion, a pairwise DEG analysis was first conducted between C9-BACexp mice and their C57BL/6J littermates, which were injected with the same vector formulations as described above. In total, we identified 836 genes whose expression was altered in the enriched nuclei from C9-BACexp mice (>1.2-FC; FDR-adjusted P < 0.05; Fig. 4h and Supplementary Data 14 and 15), 348 of which were down-regulated  and 488  of which were up-regulated (Fig. 4h), with an average FC from wild-type of 1.71 (Fig. 4j) and 1.83 (Fig. 4k), respectively. Among the DEGs, enrichment (P < 0.05) was observed for terms related to autophagy, RNA splicing and RNA regulation (Supplementary Data 16).

To determine whether RfxCas13d-N2V8 had an effect on these DEGs, we conducted a pairwise analysis between C9-BACexp and C57BL/6J mice co-injected with PHP.eB-RfxCas13d-N2V8-crRNA-13 and PHP.eB-EGFP-KASH. Of the 836 genes whose expression was altered in C9-BACexp mice injected with the non-targeted crRNA, we found that only 73 of them (~9%) were affected (that is, deviated significantly from wild-type; >1.2-FC; FDR-adjusted P < 0.05) in C9-BACexp mice treated with crRNA-13 (Fig. 4i and Supplementary Data 14 and 15), with the 488 and 348 DEGs originally found to be up- and down-regulated found to deviate from wild-type by a FC of only 1.18 and 1.16, respectively, in C9-BACexp mice treated with crRNA-13 (P < 0.0001 for both compared to NTG; Fig. 4j, k). Notably, of these 488 and 348 up- and down-regulated DEGs, we found that 401 (82%) and 253 (73%) of them, respectively, reverted back to wild-type by a FC difference of at least 50%, and that 247 (51%) and 144 (41%) of them, respectively, reverted back to wild-type by a FC difference of at least 75% (Fig. 4j, k and Supplementary Data 14 and 15). These results thus indicate that RfxCas13d can at least partially reverse a percentage of the transcriptional deficits that manifest in C9-BACexp mice.

Last for this analysis, we determined the transcriptome-wide specificity of RfxCas13d-N2V8 in vivo. Based on a pairwise comparison between C57BL/6J mice injected with PHP.eB-RfxCas13d-N2V8-crRNA-13 or PHP.eB-RfxCas13d-N2V8-NTG, we identified 84 genes that were differentially expressed by crRNA-13 (>1.25-FC; FDR-adjusted P < 0.01; Supplementary Data 17), 64 of which were up-regulated and 20 of which were down-regulated (Supplementary Data 18). Interestingly, the DEGs were found to associate with immune response-related terms, including cellular responses to interferons and host defense responses (P < 0.01; Supplementary Data 19).

In conclusion, we show that RfxCas13d and a high-fidelity version of it can be used to curb the production of the G_4_C_2_ repeat-containing RNA without affecting normal C9ORF72 mRNA levels. We further demonstrate that RfxCas13d can improve abnormalities associated with the repeat expansion in C9-BACexp mice. These results illustrate the potential of CRISPR-Cas13 technology for C9ORF72-linked ALS/FTD.

## Discussion

An abnormal expansion of a G_4_C_2_ repeat in the first intron of the *C9ORF72* gene is the most common genetic cause of ALS^7,8^, accounting for up to 40% of familial forms of the disease and 5–10% of all sporadic cases of the disorder in the United States, Europe and Australia^71^. To date, three non-exclusive mechanisms have been proposed to explain the pathogenicity of the repeat expansion^9–24^. These include haploinsufficiency or loss of C9ORF72 protein functon^9^ and/or acquired toxicity from the effects of bidirectionally transcribed repeat-containing RNAs^13,15–18^ and/or their non-canonical translation to one of five DPR proteins^19–22^.

Given the role that the repeat RNAs may play in C9-ALS/FTD, gene silencing has emerged as a promising strategy for the disorder. Accordingly, both ASOs^27,31,32,67,72^ and miRNAs^25,26^ have both been used to target the hexanucleotide repeat-containing RNA. These approaches, however, possess fundamental limitations that could limit their effectiveness for C9-ALS/FTD. ASOs, for example, have a transient lifecycle, which can require a lifetime of administrations to sustain a therapeutic effect^73,74^. This in turn can lead to periods of diminished activity and impose a physical burden on patients. Conversely, though miRNAs can be expressed for an extended period of time from a viral vector, they rely on endogenous RNA processing pathways that are predominately located in the cytoplasm^75–77^ which, in the case of C9-ALS/FTD, could limit their effectiveness, as the repeat RNAs and the RNA foci are located predominately in the nucleus^27,78,79^.

One emerging technology whose properties could overcome these limitations is CRISPR-Cas13, particularly RfxCas13d, a class II, type VI CRISPR effector protein that is: (i) capable of cleaving RNAs via its intrinsic RNase activity^33^, (ii) is compact enough to be encoded within a single AAV vector^33^ for a potential single-dose treatment and (iii) can be modified to access the nucleus^33,45^ to engage with the repeat-containing RNAs. Given these attributes, we hypothesized that RfxCas13d could be used to target the G_4_C_2_ repeat RNA to influence pathological hallmarks associated with C9-ALS/FTD.

In this proof-of-concept, we establish a CRISPR-Cas13-based system for C9-ALS/FTD. Using a dual-luciferase reporter screen to measure target engagement and non-specific collateral effects, we identify crRNAs for RfxCas13d that facilitated the targeting of the repeat RNA in HEK293T cells, SH-SY5Y cells and motor neuron-like cells derived from a patient with C9-ALS. We also find that RfxCas13d effectively curbed the expression of the G_4_C_2_ repeat RNA in C9-BACexp mice, which harbor-the full-length human *C9ORF72* gene with ~100–1000 copies of the G_4_C_2_ repeat.

Similar to other approaches for the G_4_C_2_ repeat RNA^25–27,29,30,32,39,80–82^, we chose to target the sequence(s) flanking the repeat expansion rather than the G_4_C_2_ motif itself, reasoning that: (i) the G_4_C_2_ sequence can exist within other transcripts which, if recognized by RfxCas13d, could perturb their expression and potentially lead to off-target effects and (ii) a crRNA targeting the G_4_C_2_ motif carries risk for forming secondary structure(s) that could impede its ability to target the repeat RNA. While not allele-specific, this approach nonetheless spares V2, the main C9ORF72 transcript, which is currently thought to account for ~85–95% of the C9ORF72 transcripts in the brain^25,42^ and whose transcription initiates downstream of the repeat. Thus, because V2 lacks the repeat expansion, it’s not expected to be targeted by this approach, a hypothesis we tested in HEK293T cells using western blot, which indicated that RfxCas13d did not affect the overall abundance of C9-L, the full-length C9ORF72 protein isoform encoded by V2. Thus, our data indicates this approach is not expected to exacerbate any potential haploinsufficiency.

After engaging with its target transcript, RfxCas13d undergoes a conformational change that is believed to unlock an intrinsic ability to collaterally *trans*-cleave non-target bystander RNAs^49,50,83^. As prior work has demonstrated that the degree of *trans*-cleavage can sometimes depend on the crRNA^52,53^, we hypothesized that employing a functional screen with a dedicated readout for collateral effects could aid in the identification of crRNAs that minimize this outcome. For this goal, we developed a dual-reporter system to evaluate RfxCas13d-mediated targeting. This platform consisted of: (i) a Renilla luciferase transgene whose 3’ UTR was modified to contain the target region of C9ORF72, a strategy which was previously used to identify microRNAs^25^ and ASOs^30,32^ for C9ORF72, and (ii) a constitutively expressed and unmodified firefly luciferase transgene, whose expression, if decreased, would indicate collateral *trans*-cleavage. As hypothesized, this platform enabled the discovery of crRNAs that could effectively target Renilla luciferase without affecting firefly luciferase, indicating the ability for this assay to identify crRNAs that exert relatively minimal or non-detectable collateral effects. This notwithstanding, as a region of the C9ORF72 transcript was fused to the 3′ UTR of Renilla luciferase, it’s theoretically possible that the proximity to its poly(A) signal sequence could influence its stability and/or the availability of certain crRNA binding sites, though we note that: (i) all 15 crRNAs decreased Renilla luciferase expression by >70% and (ii) 14 of the 15 crRNAs decreased it by >95%, which indicates that our screen was not confounded by such factors and that each crRNA effectively decreased the Renilla luciferase reporter. In the future, it may be practical to screen crRNAs against the endogenous C9ORF72 transcript variants in the presence of an expanded hexanucleotide repeat, particularly for approaches that aim for allele specificity, as such a screen would enable the heterozygosity of the disorder to be considered.

Given concerns for its potential to trigger collateral effects, we evaluated the ability of high-fidelity forms of RfxCas13d^53^ to target C9ORF72. Among the variants profiled, RfxCas13d-N2V8 perturbed the fewest number of non-target transcripts when programmed for C9ORF72 (230 DEGs for RfxCas13d-N2V8 versus 1214 DEGs for the native RfxCas13d protein). Among the DEGs affected by RfxCas13d-N2V8, no significant enrichment was observed for any biological functions (FDR-adjusted P > 0.05), whereas ten terms were enriched for RfxCas13d (FDR-adjusted P < 0.05). Thus, we find that coupling a dual-reporter system with high-fidelity forms of RfxCas13d can enable the identification of targeting platforms with improved specificity.

Because of its ability to mediate RNA knockdown with limited collateral effects, we also evaluated whether DiCas7-11^54^ could target C9ORF72. To our surprise, DiCas7-11 was not only less efficient than RfxCas13d at decreasing Renilla luciferase expression, but the majority of its crRNAs also reduced the expression of firefly luciferase – our reporter for collateral effects – implying non-specific activity. Given the lack of computational tools for predicting functional crRNAs for DiCas7-11, its crRNAs were designed to target the equivalent sites in C9ORF72 as the crRNAs for RfxCas13d. However, as our results indicate that these crRNAs are likely sub-optimal, a more comprehensive screen involving the tiling of C9ORF72 with crRNAs for DiCas7-11 would likely be more instructive in determining its applicability for C9-ALS/FTD.

Though C9-BACexp mice do not manifest an overt motor phenotype, we nonetheless found that RfxCas13d improved several of the deficits that do develop in this model. For example, both RfxCas13d and RfxCas13d-N2V8 reduced the accumulation of RNA foci, while RfxCas13d-N2V8 decreased the poly(GP) DPR protein. Further, we found by RNA-seq that RfxCas13d-N2V8 reversed a percentage of the transcriptional abnormalities that manifest in C9-BACexp mice, many of which were related to pathways previously found to be dysregulated in this model^56^, including those related to the extracellular matrix, cell junctions, and the immune system. Similarly, iMNs derived from a C9-ALS patient and treated with RfxCas13d-N2V8 and the repeat-targeting crRNA were found to have ~18% fewer DEGs compared to cells treated with the non-targeted crRNA, with a term analysis shown to reveal that the normalized DEGs were enriched for functions associated with RNA metabolism. Notably, however, this normalization was lower than the one observed in C9-BACexp mice, which we attribute in part to the experimental design for each study. For example, FACS was used to isolate transduced nuclei from C9-BACexp mice, whereas the iMNs were analyzed in the bulk and likely contained a greater proportion of non-transduced cells, whose RNA contents contributed to the analysis. Additionally, it’s important to note the limitations of our iPSC-based study. First, only a single iPSC line from a C9-ALS patient was used. Second, we did not determine if RfxCas13d affected the formation of RNA foci, a DPR protein, or any toxic phenotype in the iMNs. Thus, a more detailed study involving: (i) multiple iPSC lines from independent C9-ALS carriers and (ii) expanded measurements that consider additional phenotypes are needed to determine the ability of RfxCas13d to improve functional deficits in iMNs.

ASOs for the G_4_C_2_ repeat RNA developed by Wave Life Sciences (ClinicalTrials.gov NCT04931862) and Ionis Pharmaceuticals and Biogen (ClinicalTrials.gov: NCT04288856) failed to show a benefit in human trials. Though the exact reason for this remains unknown, these ASOs targeted only the G_4_C_2_ (sense) repeat RNA and were thus presumed to have no effect on the G_2_C_4_ (antisense) transcript, which is hypothesized to contribute to disease pathogenesis^15–18^. Similar to these ASOs, the approach developed here targeted only the G_4_C_2_ repeat RNA. However, RfxCas13d possesses certain advantages to ASOs that could expand opportunities for C9-ALS/FTD. For instance, RfxCas13d is genetically encodable. It thus has the theoretical ability to be continuously expressed in non-dividing cells to sustain an effect. In the case of C9-ALS/FTD, this could provide a means to provide durable neuroprotection from the presumed toxicity of the G_4_C_2_ repeat RNA in pre-symptomatic carriers or individuals at the earliest stage(s) of disease. To this point, an ASO targeting the G_4_C_2_ repeat RNA was found to prevent the emergence of certain toxic signatures in iPSC-derived neurons from C9-ALS patients^67^. Additionally, RfxCas13d is a multiplexable enzyme^33^. Thus, it has the capacity to simultaneously target both the G_4_C_2_ (sense) and G_2_C_4_ (antisense) repeat RNAs from a single vector. Though not explored here, our proof-of-concept sets the stage for the development such dual-targeting systems, which could offer benefit to post-symptomatic individuals.

To ensure its expression in the MC and HPC, two structures in C9-BACexp mice that accumulate RNA foci and produce the DPR protein poly(GP), we chose to locally deliver RfxCas13d, which we reasoned would enable a straightforward analysis of its outcome(s) for our proof-of-concept, particularly given the degree of expression that can achieved locally in mice by an intracranial injection using a relatively minimal vector dose^84^. This delivery strategy, however, is likely not clinically translatable. Thus, as a next step, it will be critical to identify the most effective AAV capsid and administration route for delivering RfxCas13d to the most affected cell populations in C9-ALS/FTD in a non-invasive manner. Two such potential administration routes are an intracerebroventricular injection and an intravenous injection, with the latter potentially offering a means to target C9ORF72 in non-nervous system tissue should that prove necessary, though a comparison of these administration routes is outside the scope of this study. Further, while neurons are principally affected in C9-ALS/FTD, other cell types, including astrocytes^85^ and microglia^86^, have also been implicated in the pathogenesis of the disorder and thus may also need to be considered in a future optimization.

Last, RfxCas13d, like other CRISPR effector proteins, can be recognized as foreign by the immune system and elicit an immunological response, which could pose a challenge for its clinical implementation, particularly for applications that require its continuous expression, such as this one. Notably, among the DEGs induced by RfxCas13d-N2V8 in C57BL6/J mice, we observed enrichment for terms related to immune function and/or cellular defense, underscoring its potential immunogenicity and the importance for implementing strategies for controlling and/or preventing immunogenic effects. This notwithstanding, we observed no measurable deficits in C9-BACexp mice injected with RfxCas13d-N2V8 and crRNA-13. However, a more comprehensive and longer-term study in mice carrying a normal *C9ORF72* allele will be needed to more definitively determine the tolerability of this approach.

In conclusion, we establish a high-fidelity CRISPR-Cas13 system to curb the expression of the G_4_C_2_ repeat RNA for C9-ALS/FTD. Our results illustrate the potential of RNA-targeting CRISPR technologies for C9-ALS/FTD and highlight the potential of CRISPR-based approaches for ALS^37,40,81,87–90^.

## Methods

### Ethical statement

All animal procedures were approved by the Institutional Animal Care and Use Committee (IACUC) at the University of Illinois Urbana-Champaign and conducted in accordance with the National Institutes of Health Guide for the Care and Use of Laboratory Animals. The protocol number for this study was 22105. This study was approved by the University of Illinois Urbana-Champaign.

### Plasmid construction

The construction of the plasmids pAAV-CAG-RfxCas13d-U6-crRNA^37^ and pAAV-CAG-DiCas7-11-U6-crRNA^40^ was previously described.

To construct pSV40-RLuc, a 27-nucleotide stuffer sequence was first inserted between the BbsI and ClaI restriction sites of psiCHECK-2 (Promega) to remove the firefly luciferase gene sequence and its promoter. A fragment of the *C9ORF72* gene encoding 20 copies of the G_4_C_2_ hexanucleotide repeat flanked by 250- and 98-bps of the upstream and downstream gene sequence, respectively, was synthesized (Genscript) and subsequently inserted between the XhoI and NotI restriction sites of the modified psiCHECK-2 plasmid.

To construct pHSV-TK-FLuc, a 32-nucleotide spacer sequence was inserted between the BglII and BbsI restriction sites of psiCHECK-2 to remove Renilla luciferase and its promoter.

To construct pAAV-CAG-RfxCas13d-N2V7-U6-cRNA and pAAV-CAG-RfxCas13d-N2V8-U6-crRNA, the native RfxCas13d gene sequence was PCR amplified from pAAV-CAG-RfxCas13d-U6-crRNA as two fragments using the primers: (1) Fusion-NcoI-Fwd-v2 with Fusion-N2V7-Rev or Fusion-N2V8-Rev; and (2) Fusion-N2V7-Fwd or Fusion-N2V8-Fwd with Fusion-SacI-Reverse (Supplementary Table 1). The resulting fusion PCR products were then ligated into the NcoI and SacI restriction sites of pAAV-CAG-RfxCas13d-U6-crRNA.

crRNAs were cloned as previously described^37^. Briefly, oligonucleotides encoding the crRNA targeting sequences were synthesized (Integrated DNA Technologies) and incubated with T4 polynucleotide kinase (NEB) for 30 min at 37°, incubated at 95 °C for 5 min, and then cooled to 4 °C at a rate of −0.1 °C/s. The duplexed and phosphorylated oligonucleotides were then ligated into the BbsI restriction sites of pAAV-CAG-RfxCas13d-U6-cRNA, pAAV-CAG-RfxCas13d-N2V7-U6-cRNA, pAAV-CAG-RfxCas13d-N2V8-U6-cRNA, and pAAV-CAG-DiCas7-11-U6-crRNA.

Sanger sequencing (ACGT) was used to confirm the identity of all plasmids. All primer sequences are provided in Supplementary Table 4.

### Cell culture, transfections, and luciferase measurements

HEK293T cells (ATCC, CRL-3216) and SH-SY5Y cells (ATCC, CRL-2266) were cultured in Dulbecco’s modified Eagle’s medium (DMEM; Corning) supplemented with 10% (v/v) fetal bovine serum (FBS; Gibco) and 1% (v/v) antibiotic-antimycotic (Gibco) in a humidified 5% CO_2_ incubator at 37 °C. Cell lines were not authenticated in-house.

For the dual-luciferase screen, HEK293T cells were seeded onto a 96 well plate at a density of 2 × 10^4^ cells per well and transfected the following day with 100 ng of either pAAV-CAG-RfxCas13d-U6-crRNA or pAAV-CAG-DiCas7-11-U6-crRNA, 1 ng of pSV40-RLuc and 10 ng of pHSV-TK-FLuc using polyethylenimine (1 mg/mL; PEI). At 72 h post-transfection, HEK293T cells were lysed with Passive Lysis Buffer (Promega). Renilla and firefly luciferase luminescence was then measured using the Dual-Glo Luciferase Assay System (Promega) using a Synergy HTX Plate Reader (BioTek). Renilla and firefly luminescence values for each sample were normalized to values from cells transfected with RfxCas13d or DiCas7-11 with a non-targeted crRNA.

For experiments involving the endogenous C9ORF72 mRNA, HEK293T cells and SH-SY5Y cells were seeded onto a 24-well plate at a density of 2 × 10^5^ cells per well and transfected the following day with 1 μg of pAAV-CAG-RfxCas13d-U6-crRNA, pAAV-CAG-RfxCas13d-N2V8-U6-crRNA, or pAAV-CAG-RfxCas13d-N2V7-U6-crRNA using Lipofectamine 3000 (Thermo Fisher Scientific) according to the manufacturer’s instructions.

### qPCR

RNA from cells and tissues was purified using the RNeasy Plus Mini Kit (Qiagen) and immediately converted to complementary DNA (cDNA) using an iScript cDNA Synthesis Kit (BioRad). qPCRs for all-V and V3 were conducted on a 96-well plate using 30 ng of cDNA using TaqMan Fast Advanced Master Mix (Thermo Fisher Scientific) with the following TaqMan probes: C9ORF72-all-V: Hs00376619_m1 (Thermo Fisher Scientific); C9ORF72-V3: Hs00948764_m1 (Thermo Fisher Scientific); HPRT1: Hs02800695_m1 (Thermo Fisher Scientific); and mouse HPRT1: MM01318743_M1 (Thermo Fisher Scientific). Reaction volumes were 20 µL and probe concentrations were: (1) 250 nM for all-V and V3, and (2) 150 nM for mouse and human HPRT1. Values were compared to HPRT1 for each respective sample and the average fold-change was calculated using the 2ΔΔCT method. All TaqMan qPCR reactions were conducted as recommended by the manufacturer’s instructions (Thermo Fisher Scientific).

CBLN1 and antisense C9ORF72 RNA measurements were performed using iTaq Universal SYBR Green Supermix (BioRad) and normalized to human GAPDH.

### AAV packaging

AAV vectors were packaged as described^91^. Briefly, 2 × 10^7^ HEK293T cells were seeded onto 15-cm plates in DMEM supplemented with 10% (v/v) FBS and 1% (v/v) antibiotic-antimycotic (Thermo Fisher Scientific). At 16 h after seeding, cells were transfected with 15 μg of pAAV-CAG-RfxCas13d-U6-crRNA-13, -7, -1 and -NTG, pAAV-CAG-RfxCas13d-N2V8-U6-crRNA-13 and -NTG, or pAAV-CAG-EGFP-KASH with 15 μg of pAAV-PHP.eB and 15 μg of pHelper using 135 μL of polyethylamine (1 μg/μL).

Cells were harvested by a cell scraper at five-days post-transfection and centrifuged at 4000 *g* for 5 min at RT. Cells were then resuspended in lysis buffer (50 mM Tris-HCl and 150 mM NaCl, pH 8.0) and subsequently freeze-thawed three consecutive times using liquid nitrogen and a 37 °C water bath. Afterward, cells were incubated with benzonase (10 units per 1 mL of suspension; Sigma-Aldrich) for 30 min at 37 °C. The suspension was then centrifuged for 30 min at 18,500 g at RT, with the ensuing supernatant layered onto an iodixanol density gradient, as described^91^. The iodixanol density gradient was centrifuged for 2 h at 140,000 *g* at 18 °C and virus was extracted, washed three times with 15 mL of PBS with 0.001% Tween-20 and concentrated to less than 250 μL using an Ultra-15 Centrifugal Filter Unit (Amicon). Viral titers were determined by qPCR using iTaq Universal SYBR Green Supermix (Bio-Rad).

### Stereotaxic injections

All procedures were approved by the Illinois Institutional Animal Care and Use Committee (IACUC) at the University of Illinois Urbana-Champaign and conducted in accordance with the National Institutes of Health (NIH) Guide for the Care and Use of Laboratory Animals. The protocol number for this study, which was approved by the University of Illinois Urbana-Champaign, was 22105. Mice were housed in humidity and temperature-controlled rooms with a 12:12 h light/dark cycle.

C9-BACexp mice [C57BL/6J-Tg(C9ORF72_i3)112Lutzy/J; Jackson Laboratory, Stock #023099] were injected at stereotaxic coordinates anterior-posterior (AP) = 1.2 mm; medial-lateral (ML) = ±1.4 mm; and dorsal-ventral (DV) = 2 mm and 1.7 mm for the MC and stereotaxic coordinates AP = −1.9 mm; ML = ±1.3 mm; and DV = 1.8 mm and 1.3 mm for the HPC. 1 × 10^10^ GCs of each vector was delivered per site in 2.4 μL of saline solution for a total of 2 × 10^10^ GCs per animal. Injections were performed using a drill and microinjection robot (NeuroStar). All experimental groups were sex and litter balanced.

### Nuclei isolation

Neuronal nuclei were isolated from dissected brain as described^64^. Briefly, tissue from the MC and HPC were dissected and homogenized in 2 mL of Nuclei EZ Lysis Buffer (Sigma-Aldrich) using a KIMBLE Dounce Tissue Grinder (Sigma-Aldrich). After the addition of 2 mL of Nuclei EZ Lysis Buffer, samples were incubated at RT for 5 min. Homogenized tissues were then centrifuged at 500 *g* for 5 min. After removing the supernatant, nuclei were resuspended in 4 mL of Nuclei Suspension Buffer (PBS with 100 µg/mL of BSA) and centrifuged at 500 *g* for an additional 5 min. The resulting pellet was then resuspended in 1 mL of Nuclei Suspension Buffer for FACS. Nuclei were strained through Round-Bottom Polystyrene Test Tubes with a 35 µm Cell Strainer Snap Cap (Falcon) and subjected to FACS using a BD FACSAria II Cell Sorter (Roy J. Carver Biotechnology Center Flow Cytometry Facility, University of Illinois Urbana-Champaign, Urbana, IL). Nuclei were collected in 350 µL of RNeasy Plus Kit Lysis Buffer (Qiagen). At least 15,000 nuclei were collected per sample.

### Immunofluorescent analysis

Immunofluorescent analyses were performed as described^87^. Briefly, extracted brains were fixed in 4% paraformaldehyde (PFA) overnight at 4 °C. Fixed tissues were then sliced to 40 µm sagittal sections on a CM3050 S cryostat (Leica) and stored in cryoprotectant solution at −20 °C. For staining, sections were washed three times in PBS for 15 min and incubated in blocking solution [PBS with 10% (v/v) donkey serum (Abcam) and 0.5% Triton X-100] for 2 h at RT. Sections were then stained with primary antibodies in blocking solution for 72 h at 4 °C. After incubation, sections were washed three times with PBS and incubated for 2 h with the secondary antibodies at RT. Sections were then washed three final times with PBS and mounted onto slides using VECTASHIELD HardSet Antifade Mounting Medium (Vector Laboratories).

Sections were imaged using a Leica TCS SP8 confocal microscope (Beckman Institute Imaging Technology Microscopy Suit, University of Illinois Urbana-Champaign, Urbana, IL). Images were analyzed using ImageJ by a blinded investigator.

Primary antibodies were rabbit anti-HA (1:500; Cell Signaling Technology, 3724S), mouse anti-NeuN (1:1000; Millipore Sigma, MAB377), rabbit anti-Iba1 (1:500; Wako Pure Chemicals Industries, 019-19741), and chicken anti-GFAP (1:1000; Abcam, ab4674).

Secondary antibodies were donkey anti-mouse Alexa Fluor 647 (1:150; Jackson ImmunoResearch, 715-605-151), donkey anti-rabbit Cy3 (1:150; Jackson ImmunoResearch, 711-165-152), donkey anti-rabbit Alexa Fluor 647 (1:150; Jackson ImmunoResearch, 711-605-152), donkey anti-chicken Alexa Fluor 647 (1:150; Jackson ImmunoResearch, 703-605-155).

### FISH

Tissue sections were incubated in blocking solution [DEPC PBS with 10% (v/v) donkey serum and 0.5% (v/v) Triton X-10] for 2 h at RT. Sections were then washed three times with DEPC PBS and incubated in hybridization solution [2x DEPC saline-sodium citrate (SSC) with 50% (v/v) formamide, 10% (w/v) dextran sulfate, 50 mM sodium phosphate and 0.5% (v/v) Triton X-100] with 40 nM of the FISH (Cy3) probe for 24 h in the dark at 37 °C and then 2 h in the dark at 66 °C. Tissue sections were then washed once in 2x DEPC SSC and subsequently washed twice with 0.1x DEPC SSC. Tissues were stained with DAPI, mounted onto slides with VECTASHIELD HardSet Antifade Mounting Medium (Vector Laboratories) and stored at 37 °C. Sections were then imaged at 40X magnification using a Leica TCS SP8 microscope (Beckman Institute Imaging Technology Microscopy Suit, University of Illinois Urbana-Champaign, Urbana, IL). To quantify foci, Cy3 RNA foci were merged with DAPI. The number of foci in all EGFP-KASH^+^ cells was then counted by a blinded investigator. Only nuclear RNA foci were counted for this analysis. For each animal, 4–10 random images were analyzed in the HPC and at least 10 random images were analyzed in the MC. All channels were despeckled to reduce background. The total number of cells analyzed per biological replicate is described in Supplementary Tables 1, 2 and 3. Images were analyzed using ImageJ.

The sequence of the FISH probe was: 5TYE563/CCCCGGCCCCGGCCCC/3TYE563. The probe was previously validated^27^ and custom-synthesized by Qiagen.

### Western blot

Cells were lysed by radioimmunoprecipitation assay (RIPA) buffer [0.2% IGEPAL CA-620, 0.02% SDS with Protease Inhibitor Cocktails (VWR Life Science, 97063-010)]. Protein concentration was then determined using the DC Protein Assay Kit (Bio-Rad). A total of 20 μg of protein per sample was electrophoresed by SDS-PAGE and electrophoretically transferred to a polyvinylidene fluoride (PVDF) membrane in transfer buffer [20 mM Tris-HCl, 150 mM glycine, and 20% (v/v) methanol] for 30 min at 100 V using a Criterion Blotter (Bio-Rad). Membranes were blocked with 5% (v/v) blotting-grade blocker (Bio-Rad) in TBS (10 mM Tris-HCl and 150 mM NaCl, pH 7.5) with 0.05% Tween-20 (TBS-T) for 1 h and then incubated with primary antibody in blocking solution at 4 °C overnight. The following primary antibodies were used: rabbit anti–β-actin (1:1000; Cell Signaling Technology, 4970S) and rabbit anti-C9ORF72 (1:1000; Proteintech, 22637-1-AP).

After incubation with primary antibody, membranes were washed three times with TBS-T and incubated with goat anti-rabbit horseradish peroxidase conjugate (1:4000; Thermo Fisher Scientific, 65-6120) in blocking solution for 1 h at room temperature (RT). Membranes were then washed three final times with TBS-T and treated with SuperSignal West Dura Extended Duration Substrate (Thermo Fisher Scientific). Chemiluminescence was detected using a ChemiDoc XRS+ (Bio-Rad). Band intensities were quantified using Image Lab Software (Bio-Rad) and normalized to the reference protein in each sample.

### RNA sequencing

Library construction was conducted by the Roy J. Carver Biotechnology Center (University of Illinois Urbana-Champaign, Urbana, IL) as previously described^37^. Briefly, RNA was purified using the RNeasy Plus Mini Kit (Qiagen) and subsequently treated with DNase. RNA was then converted into individually barcoded polyadenylated mRNA sequencing libraries using the Kapa Hyper Stranded mRNA library kit (Roche) and fused with unique dual indexes. Adaptor-ligated double-stranded cDNAs that were synthesized from the mRNA libraries were then PCR-amplified for eight cycles with KAPA HiFi DNA Polymerase (Roche), quantified by PCR and pooled in equimolar concentration. The libraries were then sequenced by a NovaSeq 6000 (Illumina) using 2 × 150 nt reads on an S1 lane. The FASTQ files that were generated from the sequencing were demultiplexed using the bcl2fastq v2.20 Conversion Software (Illumina). The quality of the demultiplexed FastQ files was then evaluated using FastQC (version 0.11.9).

Mouse RNA was purified using the RNeasy Plus Mini Kit (Qiagen) and subsequently treated with DNase. Libraries were then prepared using the Universal RNA-Seq Kit (Tecan) with probes to deplete mouse rRNAs, with the quality of each sample assessed using a 5200 Fragment analyzer (Agilent). The final barcoded RNA-seq libraries were pooled in equimolar concentration and sequenced by a NovaSeq 6000 (Illumina) using 1 × 100nt reads on one S2 lane for 101 cycles. The FASTQ files that were generated from the sequencing were demultiplexed using the bcl2fastq v2.20 Conversion Software (Illumina). The quality of the demultiplexed FastQ files was then evaluated using FastQC (version 0.11.9).

Data analysis was conducted by the High-Performance Biological Computing Core (Univ. Illinois Urbana-Champaign, Urbana, IL). Salmon (version 1.5.2 for the human study and version 1.10.0 for the mouse study) was used to quasi-map reads to the transcriptome and to quantify the abundance of each transcript. For the human study, spliced transcript sequences from Annotation Release 109.2020112 (NCBI) were used along with the GRCh38 reference genome as the decoy sequence for the Salmon index. Because reads from the mouse study contained a higher proportion retained introns, both spliced and un-spliced transcripts from Annotation Release 109 (NCBI) was used with the three main human C9ORF72 transcript variants and EGFP-KASH with the GRCm39 reference genome as the decoy sequence for the Salmon index.

Gene-level counts were estimated on transcript-level counts using the “lengthScaled TPM” method from the tximport package^92^ to provide accurate gene-level counts estimates and to keep multi-mapped reads in the analysis. DEG analysis was performed using the limma-trend method plus 1 (human) or 4 (mouse) extra factors estimated by the RUVseq package to correct for spurious technical variation^93^. An FDR adjustment was then conducted globally across the pairwise comparisons.

Overrepresentation analyses on DEGs were performed using NetworkAnalyst 3.0^94^ and Enrichr^95^ to identify the enriched biological terms associated with the differentially expressed genes using the GO:BP databases^96^.

### Cell viability

HEK293T cells and SH-SY5Y cells were seeded onto a 96-well plate at a density of 2 × 10^4^ cells per well and transfected with 100 ng of pAAV-CAG-RfxCas13d-U6-crRNA, pAAV-CAG-RfxCas13d-N2V7-U6-crRNA, or pAAV-CAG-RfxCas13d-N2V8-U6-crRNA as described. At 72 h after transfection, cells were trypsinized, washed twice with PBS and incubated with propidium iodide (10 µg/mL; Sigma Aldrich, P4170) for 15 min on ice with protection from light. Fluorescence intensity was then measured using a BD FACSymphony A1 with a High-Throughout Sampler (Roy J. Carver Biotechnology Center Flow Cytometry Facility, University of Illinois Urbana-Champaign). A total of 5,000 events were counted per well, and all data were analyzed using FlowJo v10 (FlowJo, LLC).

### Induced motor neuron differentiation

Neural progenitor cells (NPCs) derived from a 64-year-old symptomatic female ALS patient with >145 copies of the hexanucleotide repeat expansion in the *C9ORF72* gene (ax0074) and a healthy female donor (ax0016) were obtained from AXOL Bioscience. Upon receipt, NPCs were immediately resuspended at a concentration of ~2.6 × 10^5^ cells per mL in Motor Neuron Maintenance Medium (AXOL Bioscience, ax0072) with 0.2 µM Compound E (Abcam, ab142164), 0.1 µM retinoic acid (Sigma-Aldrich, R2625) and 10 µM ROCK Inhibitor (Focus Biomolecules, 10-2301). The cells were then seeded on a 48-well plate that was pre-coated with 0.5 mg/mL poly-D-Lysine (Sigma-Aldrich, P7405) and vitronectin (Thermo Fisher, A14700) at a density of ~1.3 × 10^5^ cells per well.

For the differentiation, as specified by the manufacturer’s instructions, a full volume medium exchange was conducted every other day using Complete Motor Neuron Maintenance Medium with 0.2 µM Compound E (Abcam, ab142164), 0.5 µM retinoic acid (Sigma-Aldrich, R2625), 10 ng/mL recombinant human ciliary-derived neurotrophic factor (CNTF) (ax139888), 5 ng/mL recombinant human brain-derived neurotrophic factor (BDNF) (ax139800), 10 ng/mL recombinant human glial cell line-derived neurotrophic factor (GDNF) (ax139855), and motor neuron maturation accelerator supplement (ax0179).

At 14 days in vitro, cells were treated with PHP.eB vector encoding: (i) RfxCas13d-N2V8 with either crRNA-13 or a non-targeted crRNA or (ii) EGFP, both in the presence of 1% (v/v) antibiotic-antimycotic (Gibco). Vector was added to cells at an MOI of ~2 × 10^6^.

### Immunocytochemistry

Neurospheres were fixed in 200 µL of 4% (v/v) PFA for 15 min at RT and subsequently incubated with PBS with 0.1% Triton X-100 for 10 min at RT, after which they were washed three times with PBS. Cells were then incubated in blocking solution [PBS with 10% (v/v) donkey serum (Abcam) and 0.1% Tween-20] for 30 min at RT. The cells were then incubated with primary antibodies in blocking solution overnight at 4 °C. After incubation, cells were washed three times with PBS and incubated with secondary antibodies in blocking solution for 1 h at RT, after which they were washed three times with PBS.

Primary antibodies were mouse anti-HB9 (1:200; Thermo Fisher Scientific, PA5-23407), and goat anti-ChAT (1:50; EMD Millipore, AB144P.

Secondary antibodies were donkey anti-goat Alexa Fluor 647 (1:150; Jackson ImmunoResearch, 705-605-147), and donkey anti-mouse Cy3 (1:150; Jackson ImmunoResearch, 715-165-150).

Cells were imaged using a Zeiss Observer Z1 microscope (Beckman Institute Imaging Technology Microscopy Suit, University of Illinois Urbana-Champaign, Urbana, IL). Images were processed using ImageJ imaging software.

### poly(GP) immunoassay

Meso Scale Discovery (MSD) Gold 96-well Small Spot SA SECTOR plates (L45SA; MSD) were coated with 2 µg/mL of anti-GP antibody (24494-1-AP; Proteintech) overnight at 4 °C. The following day, plates were incubated with 150 µL of 2.5% (v/v) MSD Blocker A (R93BA; MSD) in PBS for 1 h at RT with shaking (750 rpm) and then washed with PBS with 0.05% (v/v) Tween-20 (PBS-T). Plates were then incubated with 200 μg of bulk tissue lysate for 75 min at RT with shaking (750 rpm), at which point they were washed three times with 150 µL of PBS-T and incubated with 30 μL of anti-poly(GP) detection antibody (2 µg/mL; Proteintech, 24494-1-AP) for 1 h at RT with shaking (750 rpm). Plates were then washed three final times with 150 µL of PBS-T and incubated with 150 µL MSD Read Buffer B (R60AM; MSD). Signal was acquired using the MESO QuickPlex SQ 120MM plate reader (MSD).

### Behavior

All measurements were conducted by a blinded investigator. Starting at one-week post-injection, motor coordination was measured weekly using a Rotamex-5 rotarod (Columbus Instruments) by placing mice on an apparatus that accelerated from 4 to 40 rpm in 180 s, with the latency to fall recorded. Each session comprised five independent trials for each mouse. Beginning at one-week post-injection, hindlimb and forelimb grip strength were measured weekly using a Grip Strength Meter (Harvard Apparatus). Hindlimb grip strength was measured as described^87^. To measure forelimb strength, animals were held by their tail and encouraged to grasp the instrument bar with their front limbs before being pulled in the opposite direction of the device. The maximum force exerted was then recorded. Each session comprised five measurements for each animal. The weights of each mouse were recorded once per week using an electronic scale.

### AAV distribution

AAV vector genomes were quantified as described^97^. Briefly, genomic DNA from the HPC, MC, spinal cord, heart, lungs, liver, kidney, and spleen of four-month-old C9-BACexp mice was extracted using the DNeasy Blood and Tissue Kit (Qiagen). qPCR was then conducted with the PowerUp SYBR Green Master Mix (Thermo Fisher Scientific) on a 96-well plate using 20 ng of genomic DNA with the primers bGH-U6-Fwd and bGH-U6-Rev. As specified by the manufacturer, reaction volumes were 20 µL and primer concentrations were 500 nM. Vector genomes per diploid genome (vg/pg) were determined as described^97^.

### Statistical analysis and reproducibility

Statistical analysis was performed using Prism 9.1 (GraphPad Software). For in vitro and in vivo studies, groups were compared using unpaired t-tests. Statistical methods for the RNA-seq are described in the “RNA sequencing” section of the Methods. For in vitro experiments, a minimum of three independent biological replicates were used. No statistical method was used to determine sample size for in vitro experiments. For in vivo experiments, the expected effect and error were informed from published literature^32^. From this information, the target sample size for each experiment was determined by power calculations using α = 0.05 and *β* = 0.80. The sample size reflects the number of independent biological replicates, with all animals randomized into sex- and litter-balanced groups. No data were excluded from the behavior analyses. For all qPCR measurements, if no amplification or minimal amplification for housekeeping genes was observed, the sample was removed from the analysis. All RNA foci and behavior assessments were conducted by a blinded investigator.

### Reporting summary

Further information on research design is available in the Nature Portfolio Reporting Summary linked to this article.

## Supplementary information

Supplementary Information

Description of Additional Supplementary Files

Supplementary Data 1

Supplementary Data 2

Supplementary Data 3

Supplementary Data 4

Supplementary Data 5

Supplementary Data 6

Supplementary Data 7

Supplementary Data 8

Supplementary Data 9

Supplementary Data 10

Supplementary Data 11

Supplementary Data 12

Supplementary Data 13

Supplementary Data 14

Supplementary Data 15

Supplementary Data 16

Supplementary Data 17

Supplementary Data 18

Supplementary Data 19

Reporting Summary

Transparent Peer Review file

## Source data

Source data

## Data Availability

Source Data are provided as a Source Data file. The unprocessed fluorescence microscopy images for Figs. 2, 3 and 4 are available from Figshare at 10.6084/m9.figshare.27936657.v1, while the unprocessed fluorescence microscopy images for the Figures in the Supplementary Information are available at 10.6084/m9.figshare.27936666.v1. RNA-seq data have been deposited in the NCBI Gene Expression Omnibus and are accessible through GEO Series accession numbers GSE251822, GSE283702, and GSE250633. Plasmids from this study are available upon request from the corresponding author. Source data are provided with this paper.
