# Supplementary material for: A high-fidelity CRISPR-Cas13 system improves abnormalities associated with C9ORF72-linked ALS/FTD
Source: Nat Commun. 2025 Jan 8;16:460. doi: 10.1038/s41467-024-55548-5 (PMC11711314; doi:10.1038/s41467-024-55548-5)
Supplement: Supplementary file 1 — Supplementary Information [file 41467_2024_55548_MOESM1_ESM.pdf]

Supplementary Information

A high-fidelity CRISPR-Cas13 system improves abnormalities associated with C9ORF72-linked ALS/FTD

Tristan X. McCallister[1], Colin K. W. Lim[1], Mayuri Singh[1], Sijia Zhang[1], Najah S. Ahsan[1], William M. Terpstra[1], Alisha Y. Xiong[1], M. Alejandra Zeballos C[1], Jackson E. Powell[1], Jenny Drnevich[2], Yifei Kang[2], and Thomas Gaj[1,3,*]

[1] The Grainger College of Engineering, Department of Bioengineering, University of Illinois Urbana-Champaign, Urbana, IL 61801, USA

[2] High-Performance Biological Computing, Roy J. Carver Biotechnology Center, University of Illinois Urbana-Champaign, Urbana, IL, USA

[3] Carl R. Woese Institute for Genomic Biology, University of Illinois Urbana-Champaign, Urbana, IL 61801, USA

[*] Corresponding author. Email: gaj@illinois.edu

	RfxCas13d target sequence		DiCas7-11 target sequence
crRNA 1:	5'-CAAAAGAGAAGCAACCGGGCAGC-3'	crRNA 1:	5'-CAAAAGAGAAGCAACCGGGCAGCAGGGACG – 3’
crRNA 2:	5'-AAAAACAAAAACACACACCTCCT-3'	crRNA 2:	5'-AAAAACAAAAACACACACCTCCTAAACCCA – 3’
crRNA 3:	5'-GAAAAACAAAAACACACACCTCC-3'	crRNA 3:	5'-GAAAAACAAAAACACACACCTCCTAAACCC – 3’
crRNA 4:	5'-CCAAAAGAGAAGCAACCGGGCAG-3'	crRNA 4:	5'-CCAAAAGAGAAGCAACCGGGCAGCAGGGAC – 3’
crRNA 5:	5'-AAAAGAGAAGCAACCGGGCAGCA-3'	crRNA 5:	5'-AAAAGAGAAGCAACCGGGCAGCAGGGACGG – 3’
crRNA 6:	5'-GGAAAAACAAAAACACACACCTC-3'	crRNA 6:	5'-GGAAAAACAAAAACACACACCTCCTAAACC – 3’
crRNA 7:	5'-CACACCTGCTCTTGCTAGACCCC-3'	crRNA 7:	5'-CACACCTGCTCTTGCTAGACCCCGCCCCCA – 3’
crRNA 8:	5'-ACACACCTCCTAAACCCACACCT-3'	crRNA 8:	5'-ACACACCTCCTAAACCCACACCTGCTCTTG – 3’
crRNA 9:	5'-CCCCAAAAGAGAAGCAACCGGGC-3'	crRNA 9:	5'-CCCCAAAAGAGAAGCAACCGGGCAGCAGGG – 3’
crRNA 10:	5'-CACACCTCCTAAACCCACACCTG-3'	crRNA 10:	5'-CACACCTCCTAAACCCACACCTGCTCTTGC – 3’
crRNA 11:	5'-AAAACAAAAACACACACCTCCTA-3'	crRNA 11:	5'-AAAACAAAAACACACACCTCCTAAACCCAC – 3’
crRNA 12:	5'-TGGGAAAAACAAAAACACACACC-3'	crRNA 12:	5'-TGGGAAAAACAAAAACACACACCTCCTAAA – 3’
crRNA 13:	5'-CCCCCAAAAGAGAAGCAACCGGG-3'	crRNA 13:	5'-CCCCCAAAAGAGAAGCAACCGGGCAGCAGG – 3’
crRNA 14:	5'-ACACACACCTCCTAAACCCACAC-3'	crRNA 14:	5'-ACACACACCTCCTAAACCCACACCTGCTCT – 3’
crRNA 15:	5'-GGGAAAAACAAAAACACACACCT-3'	crRNA 15:	5'-GGGAAAAACAAAAACACACACCTCCTAAAC – 3’

Supplementary Fig. 1. Targeting C9ORF72 with RfxCas13d or DiCas7-11. crRNA sequences used in this study for (left) RfxCas13d and (right) DiCas7-11.

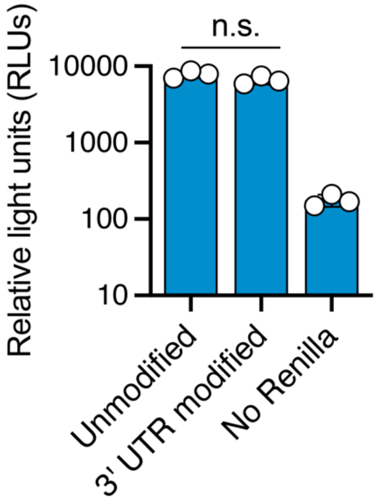

Supplementary Fig. 2. Modifying the 3' UTR of Renilla luciferase to include exon 1a and intron 1a of the C9ORF72 gene did not significantly affect its activity. Relative light units (RLUs) in HEK293T cells transfected with the unmodified form of pSV40-RLuc (unmodified), the 3 UTR-modified form of pSV40-RLuc (3' UTR modified) or no plasmid (no Renilla). Values indicate means and error bars indicate SD (n = 3). n.s., P > 0.05; two-tailed unpaired t-test. All data points are biologically independent samples. Source data and exact P values are provided in the Source Data file.

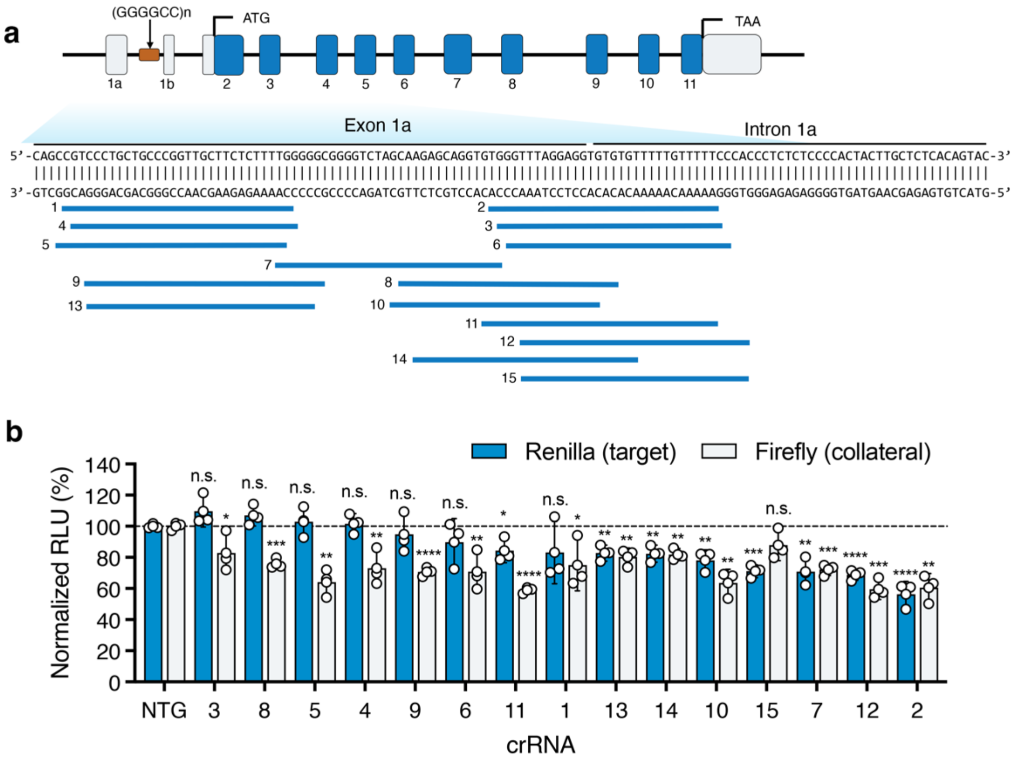

Supplementary Fig. 3. Targeting C9ORF72 with DiCas7-11. (a) Cartoon showing the locations of the crRNA binding sites for DiCas7-11 within exon 1a and intron 1a of the C9ORF72 gene. (**b**) Normalized Renilla and firefly luciferase expression in HEK293T cells transfected with pSV40-RLuc, pHSV-TK-FLuc, and an expression vector encoding DiCas7-11 and one of the 15 candidate crRNAs. All values were normalized to cells transfected with pSV40-RLuc, pHSV-TK-FLuc, and an expression vector encoding DiCas7-11 with a non-targeted (NTG) crRNA (n = 3). Values indicate means and error bars indicate SD. *P < 0.05, **P < 0.01, ***P < 0.001, ****P < 0.0001; one-tailed unpaired t-test comparing each crRNA to the NTG crRNA. All data points are biologically independent samples. Source data and exact P values are provided in the Source Data file.

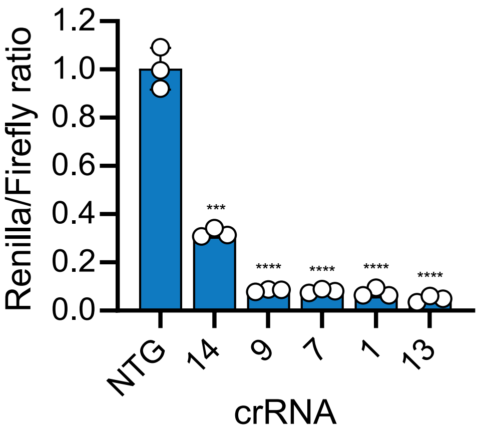

Supplementary Fig. 4. crRNA targeting scores. Targeting scores, defined as the ratio of Renilla (i.e., target) to firefly (i.e., collateral) luciferase expression, were determined for the five crRNAs that reduced firefly luciferase expression by less than two-fold. A lower targeting score indicates decreased collateral effects. Renilla and firefly luciferase expression were measured in HEK293T cells transfected with pSV40-RLuc, pHSV-TK-FLuc, and an expression vector encoding RfxCas13d and one of the 15 candidate crRNAs (n = 3). Renilla and firefly luciferase values for each crRNA were normalized to cells transfected with pSV40-RLuc, pHSV-TK-FLuc, and an expression vector encoding RfxCas13d with a non-targeted (NTG) crRNA. Bars indicate means and error bars indicate SD. ***$P < 0.001$, ****$P < 0.0001$; two-tailed unpaired t-test. All data points are biologically independent samples. Source data and exact P values are provided in the Source Data file.

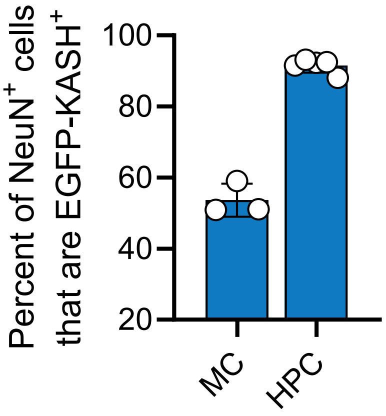

Supplementary Fig. 5. Quantification of EGFP-KASH expression in NeuN$^+$ cells in C9-BACexp mice. Percentage of NeuN$^+$ cells positive for EGFP-KASH in the motor cortex (MC) and hippocampus (HPC) of C9-BACexp mice injected with 2 x 10^{10} GCs each of PHP.eB-EGFP-KASH and PHP.eB-RfxCas13d-crRNA. 61-143 and 80-142 cells per animal were counted in the MC and HPC, respectively. 314 and 563 cells were counted in the MC and HPC, respectively ($n \geq 3$). Bars indicate means and error bars indicate SD. All data points are biologically independent samples. Source data and exact P values are provided in the Source Data file.

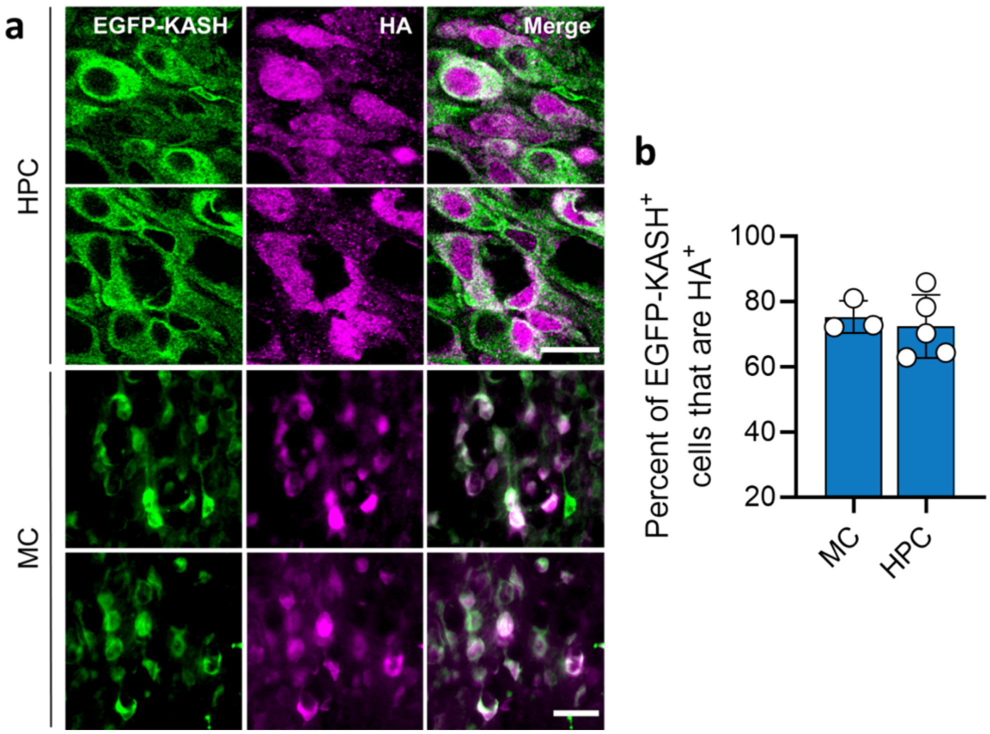

Supplementary Fig. 6. Quantification of the percentage of EGFP-KASH^{+} cells positive for RfxCas13d in C9-BACexp mice. (a) Representative immunofluorescent staining of (top) the hippocampus (HPC) and (bottom) the motor cortex (MC) of C9-BACexp mice injected with 2 x 10^{10} GCs each of PHP.eB-EGFP-KASH and PHP.eB-RfxCas13d-crRNA. Scale bar; 20 µm (HPC); 50 µm (MC). **(b)** Percentage of EGFP-KASH^{+} cells positive for RfxCas13d via its HA epitope within the injection sites in the MC and HPC of C9-BACexp mice. 79-132 and 84-212 cells were counted per animal in the MC and HPC, respectively, for a total of 316 and 715 cells counted from the MC and HPC, respectively ($n \geq 3$). Bars indicate means and error bars indicate SD. All data points are biologically independent samples. Source data are provided in the Source Data file.

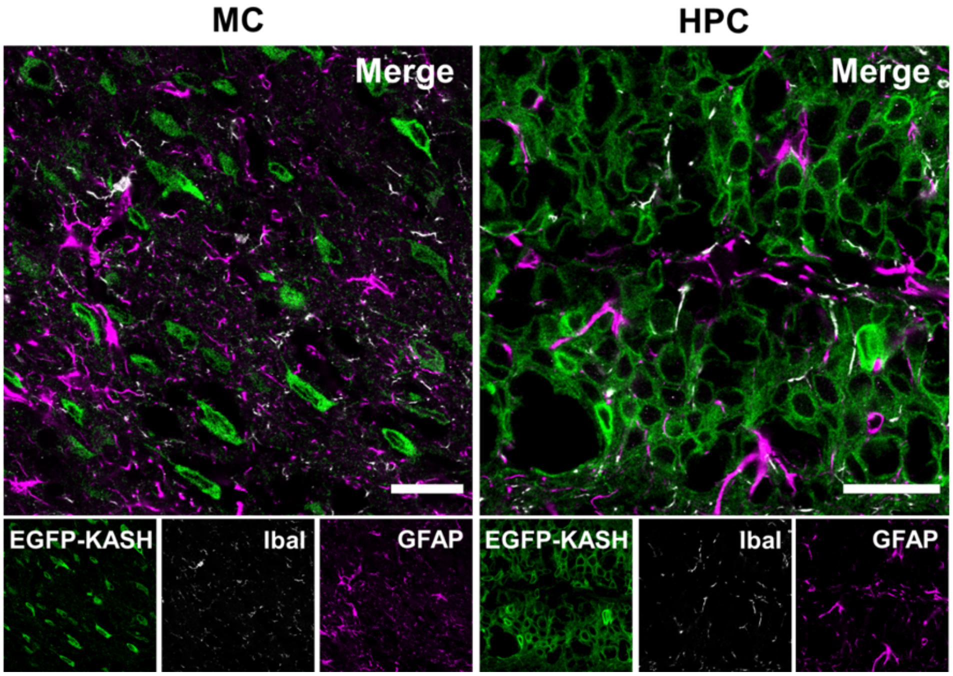

Supplementary Fig. 7. AAV-PHP.eB-EGFP-KASH injected with AAV-PHP.eB-RfxCas13d-crRNA minimally transduced microglia and astrocytes in C9-BACexp mice. Representative immunofluorescent staining of (left) the motor cortex (MC) and (right) the hippocampus (HPC) of C9-BACexp mice injected with 2 x 10^{10} GCs each of PHP.eB-EGFP-KASH and PHP.eB-RfxCas13d-crRNA. Scale bar; 30 μm. Microglia marker: Iba1, ionized calcium binding adaptor molecule 1. Astrocyte marker: GFAP, glial fibrillary acidic protein.

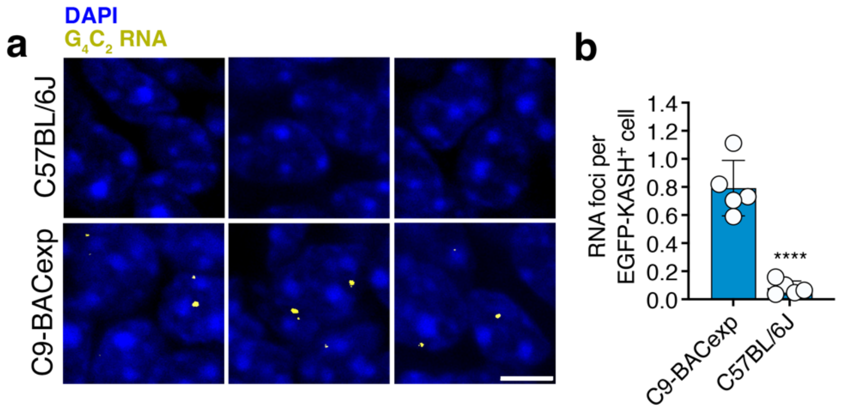

Supplementary Fig. 8. Limited RNA foci were observed in non-carrier C57BL/6J mice. (a) Representative RNA FISH for the G_4C_2 repeat RNA (yellow) in the HPC of non-carrier C57BL/6J mice or C9-BACexp mice injected with 2 x 10^{10} GCs of PHP.eB-EGFP-KASH. Arrowheads points to representative foci. Scale bar, 5 µm. (**b**) Quantification of the number of RNA foci per EGFP-KASH$^+$ cell in the HPC of non-carrier C57BL/6J mice or C9-BACexp mice (*n* = 5). The total number of cells counted per biological replicate is described in **Supplementary Tables 2 and 3.** Values indicate means and error bars indicate SD. ****P < 0.0001; one-tailed unpaired t-test. All data points are biologically independent samples. Source data and exact P values are provided in the Source Data file.

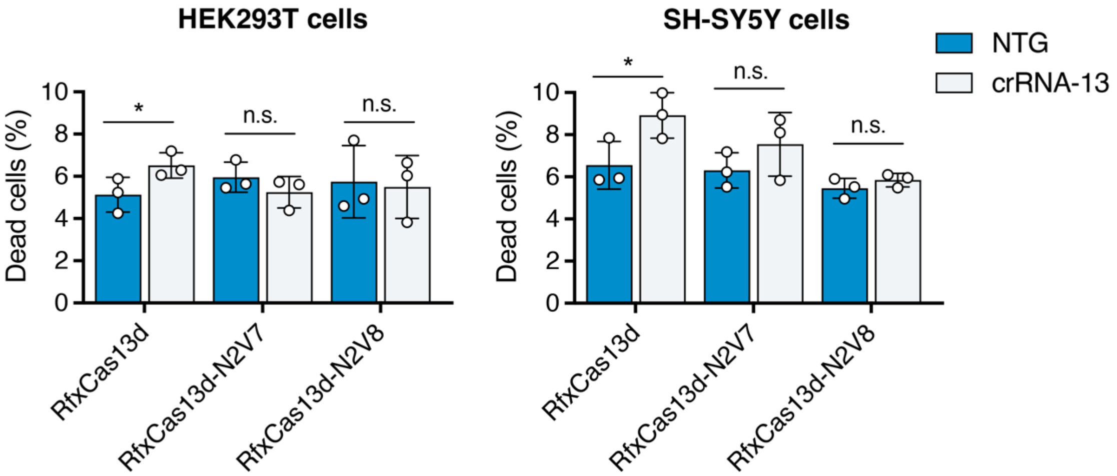

Supplementary Fig. 9. RfxCas13d-N2V8 and -N2V7 did not affect cell viability. Percentage of dead HEK293T cells and SH-SY5Y cells following their transfection with an expression vector encoding RfxCas13d, RfxCas13d-N2V7 and RfxCas13d-N2V8 with crRNA-13 or a non-targeted (NTG) crRNA, as determined by propidium iodide staining ($n = 3$). Values indicate means and error bars indicate SD. *$P < 0.05$; one-tailed unpaired t-test comparing each variant to their NTG. All data points are biologically independent samples. Source data and exact P values are provided in the Source Data file.

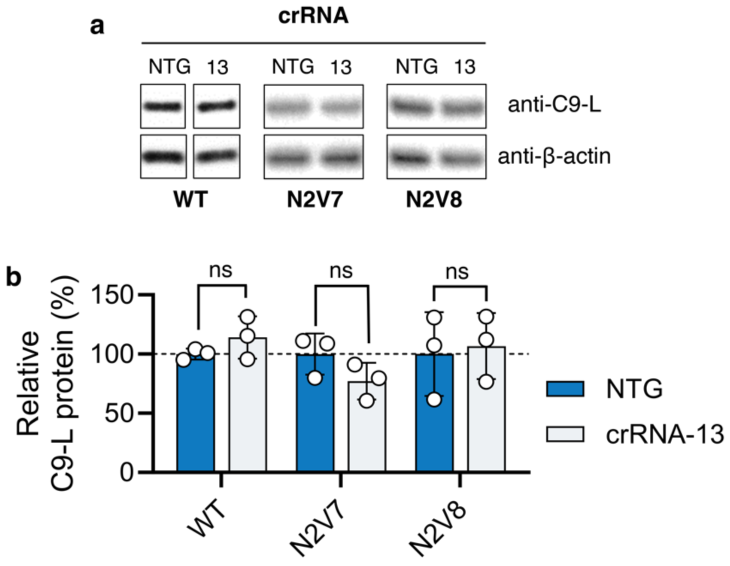

Supplementary Fig. 10. RfxCas13d targeting did not affect the abundance of the C9-L protein in HEK293T cells. (a) Representative western blot of the C9-L protein in cell lysate from HEK293T cells transfected with an expression vector encoding RfxCas13d, RfxCas13d-N2V7, or RfxCas13d-N2V8 with crRNA-13 or a non-targeted (NTG) crRNA. **(b)** Quantitation of the western blot. The C9-L protein in each lane was normalized to β-actin protein in the same lane. (n = 3). Bars represent means and error bars indicate SD. One-tailed unpaired t-test. All data points are biologically independent samples. Uncropped western blots, source data and exact P values are provided in the Source Data file.

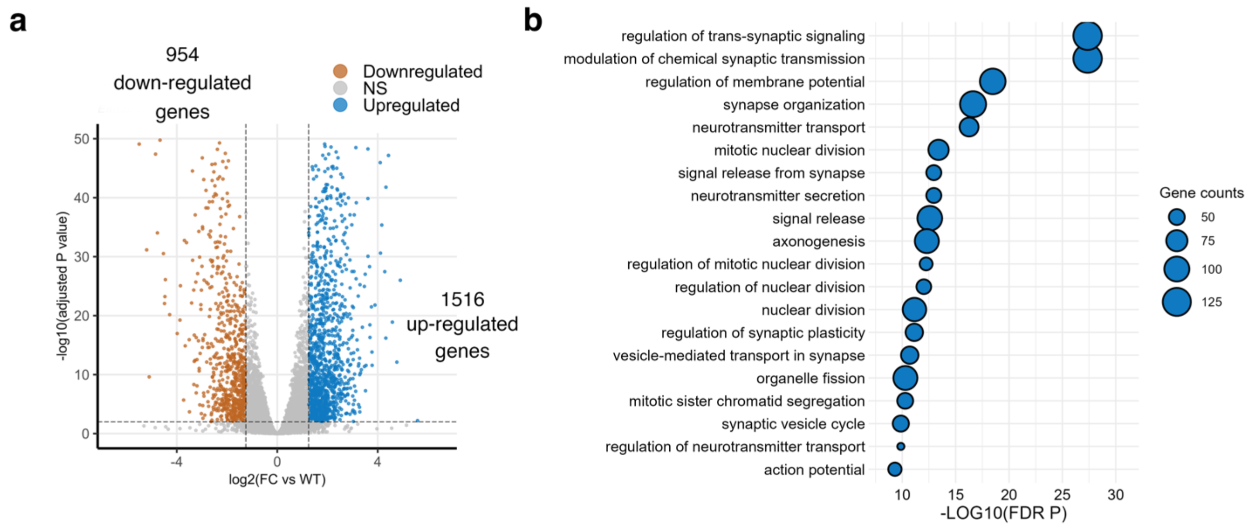

Supplementary Fig. 11. Motor neuron-like cells from a C9-ALS patient show transcriptional abnormalities. (a) Volcano plot of the RNA-seq analysis comparing iPSC-derived neurospheres from a 74-year-old female ALS patient with >145 copies of the G_4C_2 repeat in the C9ORF72 gene to a non-ALS female donor. Line denotes a >1.25-fold change (FC) and an FDR-adjusted P < 0.01 (*n* = 3). **(b)** Gene ontology (GO) and biological process (BP) term analysis for the DEGs in **(a)**.

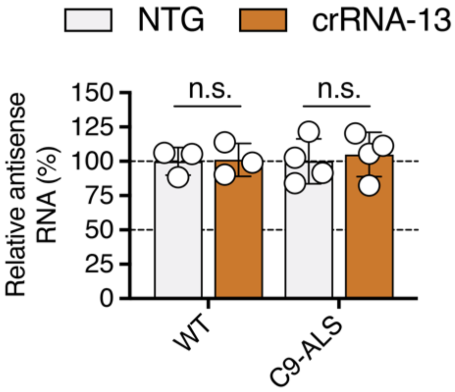

Supplementary Fig. 12. RfxCas13d-N2V8 did not affect the expression of the antisense repeat RNA in motor neuron-like cells. Relative antisense RNA in wild-type or C9-ALS neurospheres treated with PHP.eB-RfxCas13d-N2V8-crRNA-13 or -NTG ($n \geq 3$). Values indicate means and error bars indicate SD. One-tailed unpaired t-test. All data points are biologically independent samples. Source data and exact P values are provided in the Source Data file.

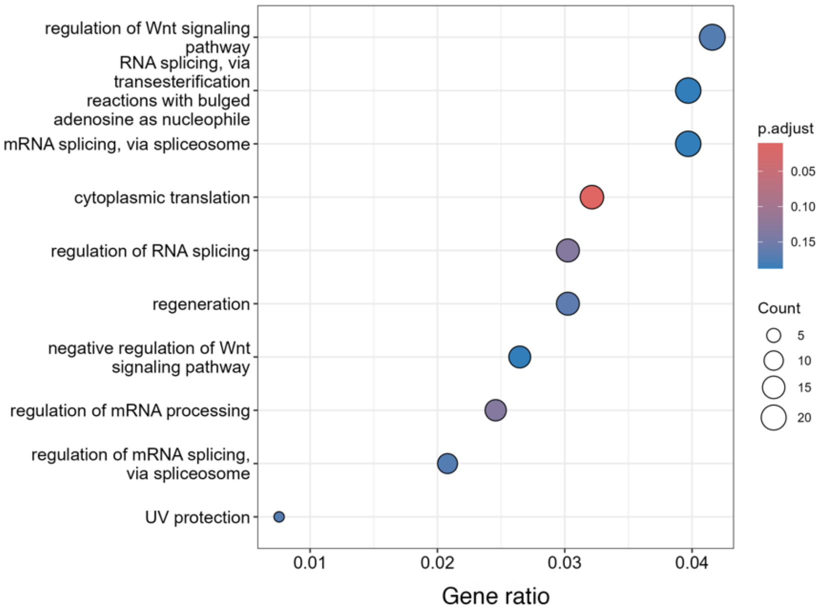

Supplementary Fig. 13. Term analysis for the normalized DEGs in RfxCas13d-N2V8-treated C9-ALS neurospheres. Gene ontology (GO) term analysis for the reverted DEGs in **Fig. 3l**.

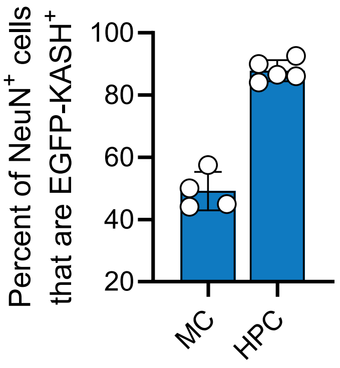

Supplementary Fig. 14. Quantification of EGFP-KASH expression in NeuN^{+} cells in C9-BACexp mice co-injected with AAV-PHP.eB-RfxCas13d-N2V8-crRNA-13. Percentage of NeuN^{+} cells positive for EGFP-KASH in the motor cortex (MC) and the hippocampus (HPC) of C9-BACexp mice injected with 2 x 10^{10} GCs each of PHP.eB-EGFP-KASH and PHP.eB-RfxCas13d-N2V8. 109-176 and 86-140 cells were counted per animal in the MC and HPC, respectively, for a total of 557 and 550 cells in the MC and HPC, respectively ($n \geq 4$). Bars indicate the means and error bars indicate SD. All data points are biologically independent samples. Source data and exact P values are provided in the Source Data file.

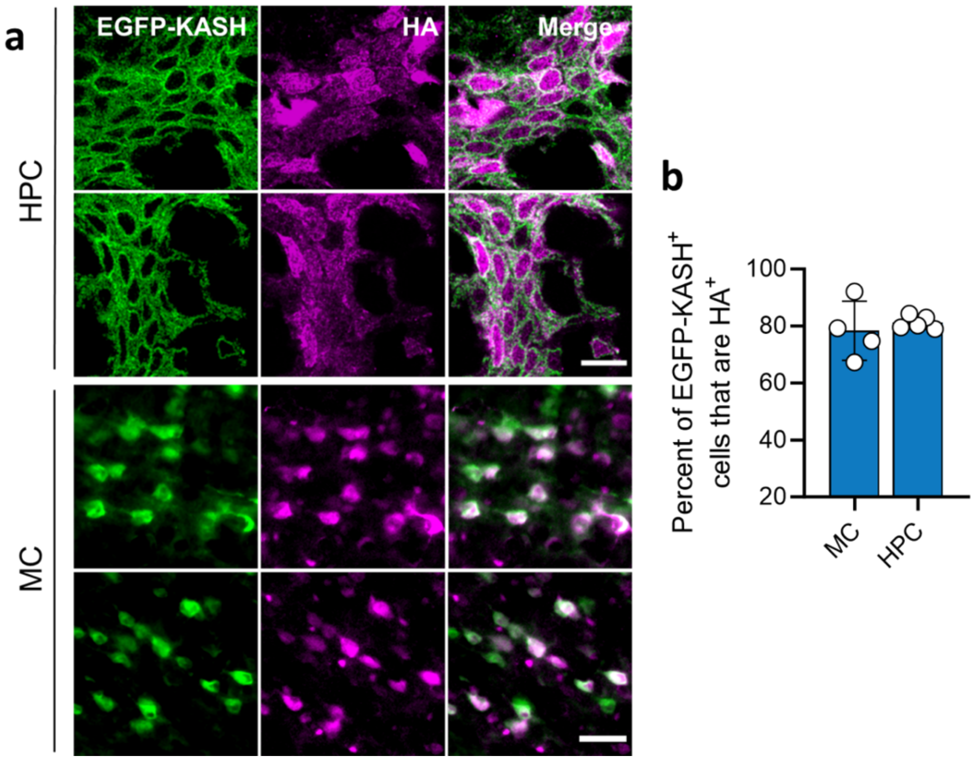

Supplementary Fig. 15. Quantification of the percentage of EGFP-KASH$^+$ cells positive for RfxCas13d-N2V8 in C9-BACexp mice. (a) Representative immunofluorescent staining of (top) the hippocampus (HPC) and (bottom) the motor cortex (MC) of C9-BACexp mice injected with 2 x 10^{10} GCs each of PHP.eB-EGFP-KASH and PHP.eB-RfxCas13d-N2V8-crRNA. Scale bar; 15 µm (HPC); 50 µm (MC). **(b)** Percentage of EGFP-KASH$^+$ cells positive for RfxCas13d-N2V8 via its HA epitope near the injection sites in the MC and HPC of C9-BACexp mice. 95-114 and 81-102 cells were counted per animal in the MC and HPC, for a total of 421 and 454 cells counted from the MC and HPC, respectively ($n \geq 4$). Bars indicate the means and error bars indicate SD. All data points are biologically independent samples. Source data and exact P values are provided in the Source Data file.

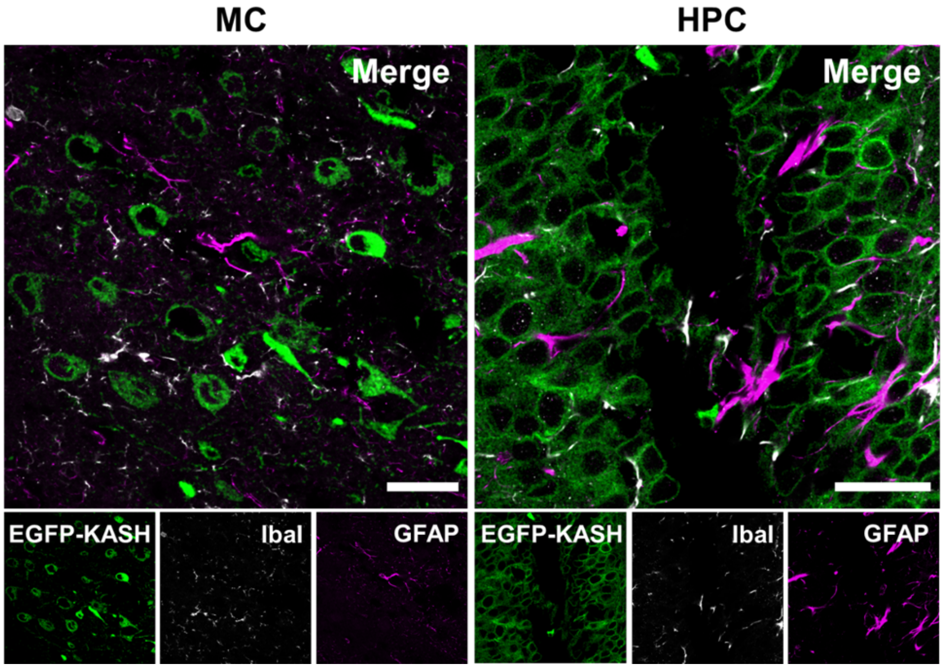

Supplementary Fig. 16. PHP.eB-EGFP-KASH co-injected with AAV-PHP.eB-RfxCas13d-N2V8-crRNA minimally transduced microglia and astrocytes in C9-BACexp mice. Representative immunofluorescent staining of (left) the motor cortex (MC) and (right) the hippocampus (HPC) of C9-BACexp mice injected with 2 x 10^{10} GCs each of PHP.eB-EGFP-KASH and PHP.eB-RfxCas13d-N2V8-crRNA. Scale bar; 30 µm. Microglia marker: Iba1, ionized calcium binding adaptor molecule 1. Astrocyte marker: GFAP, glial fibrillary acidic protein.

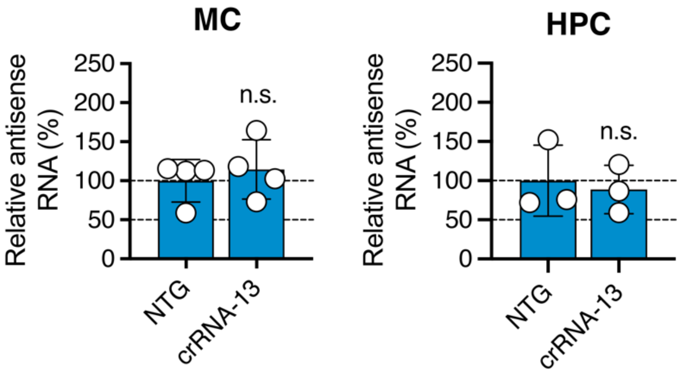

Supplementary Fig. 17. Targeting the G_4C_2 repeat RNA using RfxCas13d-N2V8 did not significantly affect the antisense RNA in C9-BACexp mice. Relative antisense repeat-containing RNA in EGFP-KASH$^+$ nuclei from the motor cortex (MC) and hippocampus (HPC) of C9-BACexp injected with 2 x 10^{10} GCs of PHP.eB-RfxCas13d-N2V8-crRNA-13 or -NTG with PHP.eB-EGFP-KASH. All values normalized to their respective NTG controls ($n \geq 3$). Values indicate means and error bars indicate SD. One-tailed unpaired t-test. All data points are biologically independent samples. Source data and exact P values are provided in the Source Data file.

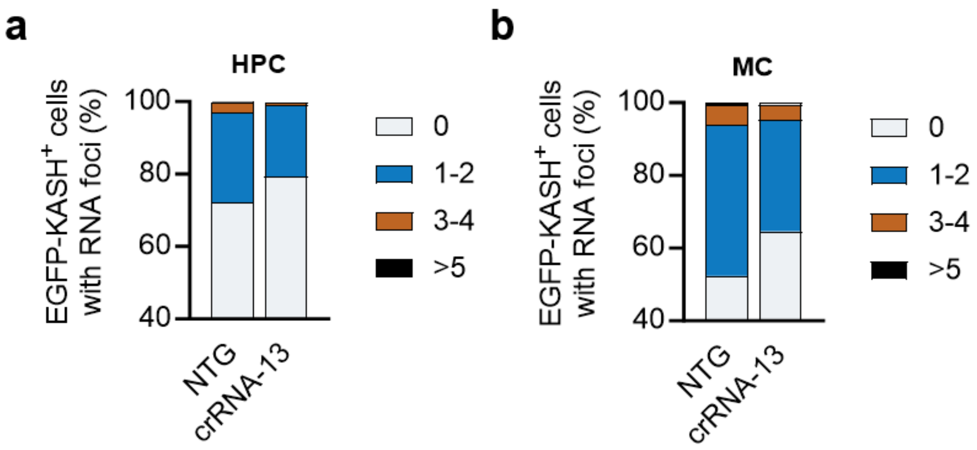

Supplementary Fig. 18. Distribution of RNA foci in EGFP-KASH+ cells from C9-BACexp mice treated with RfxCas13d-N2V8. (a, b) Percentage of EGFP-KASH+ cells with 0, 1-2, 3-4, or >5 foci positive for the G_4C_2 repeat-containing RNA in the (**a**) hippocampus (HPC) and (**b**) the motor cortex (MC) of mice injected with 2 x 10^{10} GCs of AAV-PHP.eB-RfxCas13d-N2V8-crRNA-13 or -NTG and AAV-PHP.eB-EGFP-KASH. RNA foci were determined by fluorescence in situ hybridization (FISH) using a previously described probe for the G_4C_2 repeat RNA[3]. 83-334 and 110-237 cells were counted per animal for the HPC and MC, respectively. 1,016 and 1,432 cells were counted for AAV-PHP.eB-RfxCas13d-N2V8-crRNA-13 and -NTG, respectively, for the HPC, and 946 and 1,139 cells were counted for AAV-PHP.eB-RfxCas13d-N2V8-crRNA-13 and -NTG, respectively, for the MC ($n \geq 6$). All measurements were conducted by a blinded investigator. Values indicate means. Source data and exact P values are provided in the Source Data file.

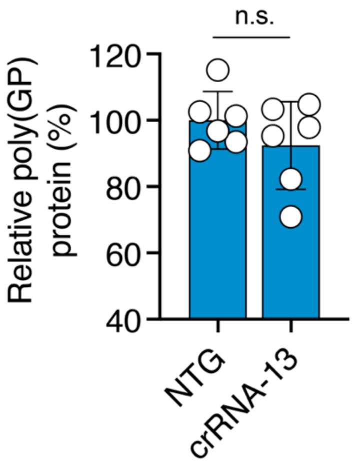

Supplementary Fig. 19. RfxCas13d-N2V8 reduced the poly(GP) protein by ~8% in bulk cortical tissue from C9-BACexp mice. Soluble poly(GP) from the motor cortex (MC) of C9-BACexp mice injected with PHP.eB-RfxCas13d-N2V8-crRNA-13 or -NTG, as measured by a MSD immunoassay platform (n = 6). Values indicate means and error bars indicate SD. n.s., $P > 0.05$; one-tailed unpaired t-test. All data points are biologically independent samples. Source data and exact P values are provided in the Source Data file.

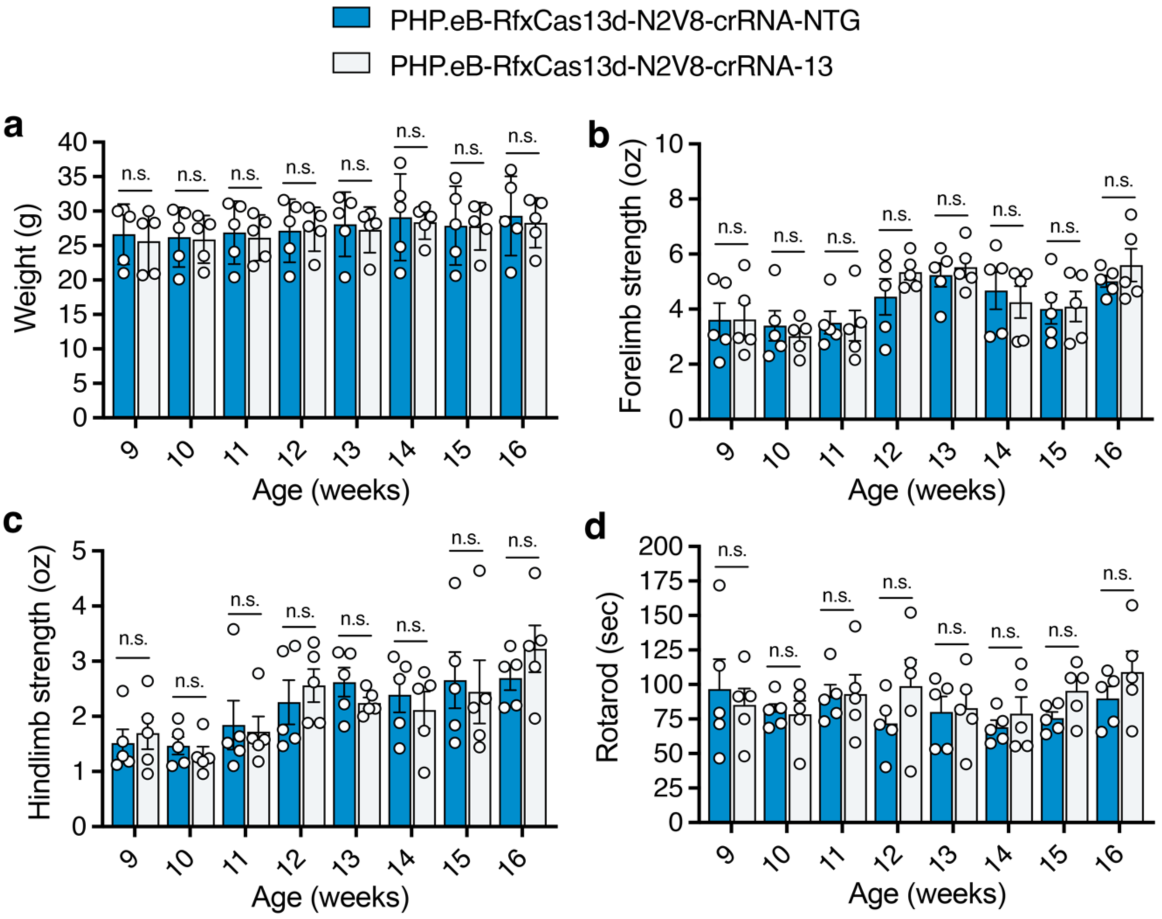

Supplementary Fig. 20. RfxCas13d-N2V8 did not induce measurable deficits in C9-BACexp mice. (a) Weight, (**b**) forelimb grip strength, (**c**) hindlimb grip strength and (**d**) rotarod of C9-BACexp mice whose HPC and MC were injected with 2 x 10^{10} GCs of PHP.eB-RfxCas13d-N2V8-crRNA-13 or -NTG. ($n = 5$). Values represent means and error bars indicate SEM. $^*P < 0.05$; two-tailed unpaired t-test. All data points are biologically independent samples. Source data and exact P values are provided in the Source Data file.

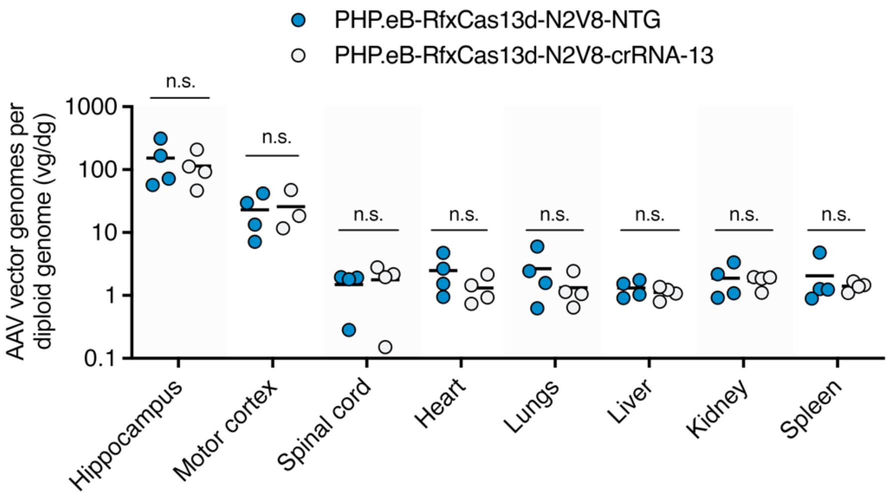

Supplementary Fig. 21. PHP.eB vector distribution in C9-BACexp mice. Vector genomes per diploid genome (vg/dpg) as measured by qPCR in the hippocampus (HPC), motor cortex (MC), spinal cord, heart, lungs, liver, kidney and spleen of C9-BACexp mice whose HPC and MC were injected with 2 x 10^{10} GCs of PHP.eB-RfxCas13d-N2V8-crRNA-13 or -NTG. (n = 4). Values represent means. Data were compared using a two-tailed unpaired t-test. All data points are biologically independent samples. Source data and exact P values are provided in the Source Data file.

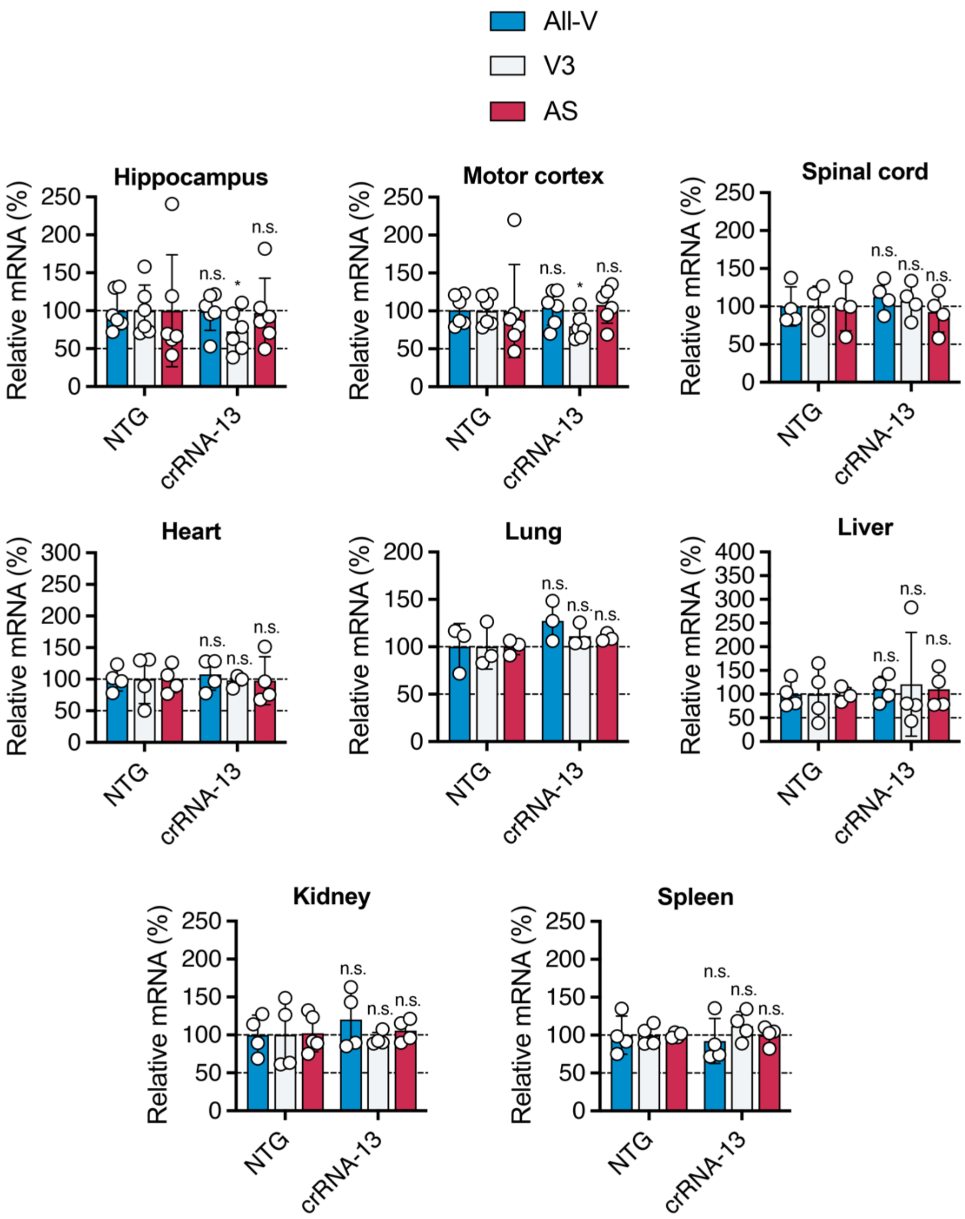

Supplementary Fig. 22. RfxCas13d-N2V8 did not measurably affect C9ORF72 transcripts in peripheral tissues. Relative all-V and V3 mRNA and antisense RNA in the hippocampus (HPC), motor cortex (MC), spinal cord, heart, lung, liver, kidney and spleen from C9-BACexp mice whose HPC and MC were injected with 2 x 10^{10} GCs of PHP.eB-RfxCas13d-N2V8-crRNA-13 or -NTG. All-V, V3, and antisense RNA values were normalized to their respective NTG control for that same tissue ($n \geq 5$). All analyses were conducted with RNA purified from bulk tissue. Values indicate means and error bars indicate SD. *$P < 0.05$; one-tailed unpaired t-test. All data

points are biologically independent samples. Source data and exact P values are provided in the Source Data file.

Supplementary Table 1. Cell counts for the FISH analyses in C9-BACexp mice injected with PHP.eB-RfxCas13d-crRNA-13, -7, or -NTG.

Biological Replicate					
**Group**	**1**	**2**	**3**	**4**	**5**
NTG	111	116	67	61	112
crRNA-7	94	138	93	58	
crRNA-13	189	455	129	72	

Supplementary Table 2. Cell counts for the FISH analysis in C57BL/6J mice injected with PHP.eB-EGFP-KASH.

Biological Replicate					
**Group**	**1**	**2**	**3**	**4**	**5**
C57BL/6J	171	232	236	357	269

Supplementary Table 3. Cell counts for RNA FISH analysis from mice injected with RfxCas13d-N2V8.

Biological Replicate								
**Region**	**Group**	**1**	**2**	**3**	**4**	**5**	**6**	**7**
MC	NTG	212	150	110	169	171	135	192
	crRNA-13	237	184	127	121	124	153	
HPC	NTG	220	117	159	211	224	167	334
	crRNA-13	231	189	193	83	147	173	

Supplementary Table 4. Primer sequences used in this study.

Name	Sequence (5' to 3')
RfxCas13d-C9ORF72-crRNA-1-Fwd	AAACCAAAAGAGAAGCAACCGGGCAGC
RfxCas13d-C9ORF72-crRNA-1-Rev	AAAAGCTGCCCGGTTGCTTCTCTTTTG
RfxCas13d-C9ORF72-crRNA-2-Fwd	AAACAAAAACAAAAACACACACCTCCT
RfxCas13d-C9ORF72-crRNA-2-Rev	AAAAAGGAGGTGTGTGTTTTTGTTTTT
RfxCas13d-C9ORF72-crRNA-3-Fwd	AAACGAAAAACAAAAACACACACCTCC
RfxCas13d-C9ORF72-crRNA-3-Rev	AAAAGGAGGTGTGTGTTTTTGTTTTTC
RfxCas13d-C9ORF72-crRNA-4-Fwd	AAACCCAAAAGAGAAGCAACCGGGCAG
RfxCas13d-C9ORF72-crRNA-4-Rev	AAAACTGCCCGGTTGCTTCTCTTTTGG
RfxCas13d-C9ORF72-crRNA-5-Fwd	AAACAAAAGAGAAGCAACCGGGCAGCA
RfxCas13d-C9ORF72-crRNA-5-Rev	AAAATGCTGCCCGGTTGCTTCTCTTTT
RfxCas13d-C9ORF72-crRNA-6-Fwd	AAACGGAAAAACAAAAACACACACCTC
RfxCas13d-C9ORF72-crRNA-6-Rev	AAAAGAGGTGTGTGTTTTTGTTTTTCC
RfxCas13d-C9ORF72-crRNA-7-Fwd	AAACCACACCTGCTCTTGCTAGACCCC
RfxCas13d-C9ORF72-crRNA-7-Rev	AAAAGGGGTCTAGCAAGAGCAGGTGTG
RfxCas13d-C9ORF72-crRNA-8-Fwd	AAACACACACCTCCTAAACCCACACCT
RfxCas13d-C9ORF72-crRNA-8-Rev	AAAAAGGTGTGGGTTTAGGAGGTGTGT
RfxCas13d-C9ORF72-crRNA-9-Fwd	AAACCCCCAAAAGAGAAGCAACCGGGC
RfxCas13d-C9ORF72-crRNA-9-Rev	AAAAGCCCGGTTGCTTCTCTTTTGGGG
RfxCas13d-C9ORF72-crRNA-10-Fwd	AAACCACACCTCCTAAACCCACACCTG

RfxCas13d-C9ORF72-crRNA-10-Rev	AAAACAGGTGTGGGTTTAGGAGGTGTG
RfxCas13d-C9ORF72-crRNA-11-Fwd	AAACAAAACAAAAACACACACCTCCTA
RfxCas13d-C9ORF72-crRNA-11-Rev	AAAATAGGAGGTGTGTGTTTTTGTTTT
RfxCas13d-C9ORF72-crRNA-12-Fwd	AAACTGGGAAAAACAAAAACACACACC
RfxCas13d-C9ORF72-crRNA-12-Rev	AAAAGGTGTGTGTTTTTGTTTTTCCCA
RfxCas13d-C9ORF72-crRNA-13-Fwd	AAACCCCCCAAAAGAGAAGCAACCGGG
RfxCas13d-C9ORF72-crRNA-13-Rev	AAAACCCGGTTGCTTCTCTTTTGGGGG
RfxCas13d-C9ORF72-crRNA-14-Fwd	AAACACACACACCTCCTAAACCCACAC
RfxCas13d-C9ORF72-crRNA-14-Rev	AAAAGTGTGGGTTTAGGAGGTGTGTGT
RfxCas13d-C9ORF72-crRNA-15-Fwd	AAACGGGAAAAACAAAAACACACACCT
RfxCas13d-C9ORF72-crRNA-15-Rev	AAAAAGGTGTGTGTTTTTGTTTTTCCC
Cas7-11-C9ORF72-crRNA-1-Fwd	GAACCAAAAGAGAAGCAACCGGGCAGCAGGGAC G
Cas7-11-C9ORF72-crRNA-1-Rev	AAAA CGTCCCTGCTGCCCGGTTGCTTCTCTTTTG
Cas7-11-C9ORF72-crRNA-2-Fwd	GAACAAAAACAAAAACACACACCTCCTAAACCCA

Cas7-11-C9ORF72-crRNA-2-Rev	AAAATGGGTTTAGGAGGTGTGTGTTTTTGTTTTT
Cas7-11-C9ORF72-crRNA-3-Fwd	GAACGAAAAACAAAAACACACACCTCCTAAACCC
Cas7-11-C9ORF72-crRNA-3-Rev	AAAAGGGTTTAGGAGGTGTGTGTTTTTGTTTTTC
Cas7-11-C9ORF72-crRNA-4-Fwd	GAACCCAAAAGAGAAGCAACCGGGCAGCAGGGAC
Cas7-11-C9ORF72-crRNA-4-Rev	AAAAGTCCCTGCTGCCCGGTTGCTTCTCTTTTGG
Cas7-11-C9ORF72-crRNA-5-Fwd	GAACAAAAGAGAAGCAACCGGGCAGCAGGGACG G
Cas7-11-C9ORF72-crRNA-5-Rev	AAAACCGTCCCTGCTGCCCGGTTGCTTCTCTTTT
Cas7-11-C9ORF72-crRNA-5-Fwd	GAACGGAAAAACAAAAACACACACCTCCTAAACC
Cas7-11-C9ORF72-crRNA-6-Rev	AAAAGGTTTAGGAGGTGTGTGTTTTTGTTTTTCC
Cas7-11-C9ORF72-crRNA-7-Fwd	GAACCACACCTGCTCTTGCTAGACCCCGCCCCCA
Cas7-11-C9ORF72-crRNA-7-Rev	AAAATGGGGGCGGGGTCTAGCAAGAGCAGGTGTG
Cas7-11-C9ORF72-crRNA-8-Fwd	GAACACACACCTCCTAAACCCACACCTGCTCTTG
Cas7-11-C9ORF72-crRNA-8-Rev	AAAACAAGAGCAGGTGTGGGTTTAGGAGGTGTGT
Cas7-11-C9ORF72-crRNA-9-Fwd	GAACCCCCAAAAGAGAAGCAACCGGGCAGCAGGG

Cas7-11-C9ORF72-crRNA-9-Rev	AAAACCCTGCTGCCCGGTTGCTTCTCTTTTGGGG
Cas7-11-C9ORF72-crRNA-10-Fwd	GAACCACACCTCCTAAACCCACACCTGCTCTTGC
Cas7-11-C9ORF72-crRNA-10-Rev	AAAAGCAAGAGCAGGTGTGGGTTTAGGAGGTGTG
Cas7-11-C9ORF72-crRNA-11-Fwd	GAACAAAACAAAAACACACACCTCCTAAACCCAC
Cas7-11-C9ORF72-crRNA-11-Rev	AAAAGTGGGTTTAGGAGGTGTGTGTTTTTGTTTT
Cas7-11-C9ORF72-crRNA-12-Fwd	GAACTGGGAAAAACAAAAACACACACCTCCTAAA
Cas7-11-C9ORF72-crRNA-12-Rev	AAAATTTAGGAGGTGTGTGTTTTTGTTTTTCCCA
Cas7-11-C9ORF72-crRNA-13-Fwd	GAACCCCCCAAAAGAGAAGCAACCGGGCAGCAGG
Cas7-11-C9ORF72-crRNA-13-Rev	AAAACCTGCTGCCCGGTTGCTTCTCTTTTGGGGG
Cas7-11-C9ORF72-crRNA-14-Fwd	GAACACACACACCTCCTAAACCCACACCTGCTCT
Cas7-11-C9ORF72-crRNA-14-Rev	AAAAAGAGCAGGTGTGGGTTTAGGAGGTGTGTGT
Cas7-11-C9ORF72-crRNA-15-Fwd	GAACGGGAAAAACAAAAACACACACCTCCTAAAC
Cas7-11-C9ORF72-crRNA-15-Rev	AAAAGTTTAGGAGGTGTGTGTTTTTGTTTTTCCC
C9-BACexp-Genotype-Fwd	TGTTTGCCTGCAATAGGC

C9-BACexp-Genotype-Rev	TGATGGGAAAGCTATTATGACC
Rluc-Stuffer-Fwd	AAATGTCGAGGTGAGATAAGCAGAT
Rluc-Stuffer-Rev	CGATCTGCTTATCTCACCTCGAC
Fluc-Stuffer-Fwd	GATCTATAAGCAGGTCGAGGTGAG
Fluc-Stuffer-Rev	ATTTCTCACCTCGACCTGCTTAT
qPCR-hASC9-Fwd	AGTCGCTAGAGGCGAAAGC
qPCR-hASC9-Rev	CGAGTGGGTGAGTGAGGAG
qPCR-hCBLN1-Fwd	TCAGAACGCAGCACTTTCATC
qPCR-hCBLN1-Rev	TTTAGCATGAGGCTCACCTGT
qPCR-hGAPDH-Fwd	CCTGACCTGCCGTCTAGAAAA
qPCR-hGAPDH-Rev	CTCCGACGCCTGCTTCAC
qPCR-bGHpA-Fwd	GCCTTCTAGTTGCCAGCCAT
qPCR-bGHpA-Rev	GGCACCTTCCAGGGTCAAG
qPCR-EGFP-KASH-Fwd	GACGACGGCAACTACAAGACC
qPCR-EGFP-KASH-Rev	CTTCAAGGAGGACGGCAACATC
qPCR-bGH-U6-Fwd	CATTGTCTGAGTAGGTGTCATTC
qPCR-bGH-U6-Rev	TCTCTAACAGCCTTGTATCGT
Fusion-NcoI-Fwd-v2	CTATTACCATGGTCGAGGTGAGCCCCACGTTCTGC TTCACTC
Fusion-SacI-Reverse	AGGGCCTTGAGCTCATCATAGGACAGGTTGGTTCC TAAAATACGG ATG

Fusion-N2V7-Fwd	GCCGCCTACGCCACCAACGCCGCCTACGCCGTCAA CAATGCCTCCGGCCTGGATAAGGACATTATTGGAT TCGGCAAGTTCTCCACAGTG
Fusion-N2V7-Rev	TGACGGCGTAGGCGGCGTTGGTGGCGTAGGCGGC GAGGGCTTTTTCAATGTCCAGGATGTTATGGATCA CCTGGATACAAATAT TGTCAT
Fusion-N2V8-Fwd	TCGTGGAATACATTACCAACGTGGTGTACGTGGTC AACAATATCTCCGGCCTGGATAAGGACATTATTGG ATTCGGCAAGTTCTCCACAG
Fusion-N2V8-Rev	ACCACGTACACCACGTTGGTAATGTATTCCACGAG GATTTTTTCAATGTCCAGGATGTTATGGATCACCT GGATACAAATATTGTCATTG

REFERENCES

1. Wessels, H.H. et al. Massively parallel Cas13 screens reveal principles for guide RNA design. *Nat. Biotechnol.* **38**, 722-727 (2020).
2. Guo, X. et al. Transcriptome-wide Cas13 guide RNA design for model organisms and viral RNA pathogens. *Cell Genom.* **1** (2021).
3. Lagier-Tourenne, C. et al. Targeted degradation of sense and antisense C9orf72 RNA foci as therapy for ALS and frontotemporal degeneration. *Proc. Natl. Acad. Sci. U. S. A.* **110**, E4530-4539 (2013).
