# Supplementary material for: A high-fidelity CRISPR-Cas13 system improves abnormalities associated with C9ORF72-linked ALS/FTD
Source: Nat Commun. 2025 Jan 8;16:460. doi: 10.1038/s41467-024-55548-5 (PMC11711314; doi:10.1038/s41467-024-55548-5)
Supplement: Supplementary file 2 — Description of Additional Supplementary Files [file 41467_2024_55548_MOESM2_ESM.pdf]

Description of Additional Supplementary Files

Supplementary Data 1. Processed RNA-seq data from HEK293T cells.

Supplementary Data 2. List of DEGs and shared DEGs from HEK293T cells between RfxCas13d, RfxCas13d-N2V7 and RfxCas13d-N2V8, with crRNA-13.

Supplementary Data 3. Gene ontology (GO) and biological process (BP) term analysis of DEGs from HEK293T cells for RfxCas13d with crRNA-13 using NetworkAnalyst 3.0.

Supplementary Data 4. Gene ontology (GO) and biological process (BP) term analysis of DEGs from HEK293T cells for RfxCas13d-N2V8 with crRNA-13 using NetworkAnalyst 3.0.

Supplementary Data 5. Gene ontology (GO) and biological process (BP) term analysis of shared DEGs from HEK293T cells for RfxCas13d and RfxCas13d-N2V8 with crRNA-13 using NetworkAnalyst 3.0.

Supplementary Data 6. Processed RNA-seq data from iPSC-derived neurospheres cells.

Supplementary Data 7. List of DEGs in C9-ALS neurospheres

Supplementary Data 8. Gene ontology (GO) and biological process (BP) term analysis of DEGs from C9-ALS neurospheres

Supplementary Data 9. Processed RNA-seq data from wild-type iPSC-derived neurospheres cells treated with RfxCas13d-N2V8-crRNA-13

Supplementary Data 10. List of DEGs in WT neurospheres treated with RfxCas13d-N2V8-crRNA-13 compared to RfxCas13d-N2V8-NTG

Supplementary Data 11. Processed RNA-seq data from C9-ALS neurospheres cells treated with RfxCas13d-N2V8-crRNA-13

Supplementary Data 12. List of DEGs and shared DEGs from C9-ALS neurospheres treated with RfxCas13d-N2V8-crRNA-13.

Supplementary Data 13. Gene ontology (GO) and biological process (BP) term analysis of normalized DEGs in C9-ALS neurospheres treated with RfxCas13d-N2V8-crRNA-13

Supplementary Data 14. Processed RNA-seq data from C9-BACexp or C57BL/6J mice injected with RfxCas13d-N2V8-crRNA-13 (S13) or RfxCas13d-N2V8-crRNA-13 (NTG; Tg: C9-BACexp mice; nTg: C57BL/6J mice)

Supplementary Data 15. Fold-change and FDR comparisons between DEGs from C9-BACexp or C57BL/6J mice injected with RfxCas13d-N2V8-crRNA-13 (S13) or RfxCas13d-N2V8-crRNA-13 (NTG; Tg: C9-BACexp mice; nTg: C57BL/6J mice)

Supplementary Data 16. Gene ontology (GO) and biological process (BP) term analysis of DEGs from from C9-BACexp or C57BL/6J mice injected with RfxCas13d-N2V8-crRNA-13 (S13) or RfxCas13d-N2V8-crRNA-13 (NTG; Tg: C9-BACexp mice; nTg: C57BL/6J mice). Yellow indicates FDR Adjusted P < 0.05

Supplemetary Data 17. Processed RNA-seq for RfxCas13d-N2V8-crRNA-13 vs.RfxCas13d-N2V8-NTG comparison in EGFP-KASH+ nuclei from C57BL/6J mice.

Supplementary Data 18. DEGs for the RfxCas13d-N2V8-crRNA-13 vs.RfxCas13d-N2V8-NTG comparison in EGFP-KASH+ nuclei from C57BL/6J mice.

Supplementary Data 19. Gene ontology (GO) and biological process (BP) term analysis for the DEGs from the RfxCas13d-N2V8-crRNA-13 vs.RfxCas13d-N2V8-NTG comparison in EGFP-KASH+ nuclei from C57BL/6J mice.
