# Supplementary material for: A high-fidelity CRISPR-Cas13 system improves abnormalities associated with C9ORF72-linked ALS/FTD
Source: Nat Commun. 2025 Jan 8;16:460. doi: 10.1038/s41467-024-55548-5 (PMC11711314; doi:10.1038/s41467-024-55548-5)
Supplement: Supplementary file 23 — Transparent Peer Review file [file 41467_2024_55548_MOESM23_ESM.pdf]

nature portfolio

Peer Review File

A high-fidelity CRISPR-Cas13 system improves abnormalities associated with C9ORF72-linked ALS/FTD

Corresponding Author: Professor Thomas Gaj

This file contains all reviewer reports in order by version, followed by all author rebuttals in order by version.

Attachments originally included by the reviewers as part of their assessment can be found at the end of this file.

Version 0:

Reviewer comments:

Reviewer #1

(Remarks to the Author)
The hexanucleotide GGGGCC repeat expansion in the C9ORF72 gene is the most common genetic cause of ALS and FTD. One disease mechanism is the toxicity caused by the repeat RNA. Reduction of the repeat containing transcript can potentially reduce the defects. In this manuscript, the authors developed an RNA-targeting CRISPR based platform to reduce the C9ORF72 repeat RNA without affecting the normal C9ORF72 levels, using a Cas13 variant with reduced collateral effects. The method was tested in both cell lines, and patient derived iPS-neurons and mouse models. Overall, the study suggested a new method to decrease the sense repeat RNA as a novel therapeutic strategy. More evidence is required to support the specificity and efficacy of the method.
1. The RNA FISH data are not convincing. In both Fig 2f and 4e, the RNA foci were not visible. It is not clear whether there were real RNA foci compared to negative control (wild type mice). It is also not clear how the foci were quantified. How many cells were counted per biological replicate? In Fig 2g, there were huge variation between replicates. The animal number need to be increased to draw conclusions.
2. At least three independent iPSC lines (from different individuals) are needed to confirm the effect of the Cas13 platform.
3. Control iPSC lines should also be included, especially for the gene expression rescue experiments (Fig 3k). RNA-seq should be performed on both control and patient neurons to define the disease-linked profile, and then determine whether this profile can be rescued by Cas13. Also whether the Cas13 system also affects the CBLN1 and CBLN2, as well as transcriptome-wide gene expression levels in control cells, need to be tested to confirm the effect is due to the C9 repeat reduction.
4. RNA foci and poly-GP levels should be measured in iPS-neurons. Whether the Cas13 platform can rescue the neuron toxicity phenotypes (such as glutamate induced toxicity) should also be measured.
5. The antisense RNA levels should be measured by RT-qPCR and RNA FISH to test whether this method influences the antisense transcripts.
6. For mouse studies, any disease or pathological phenotypes can be rescued by the CRISPR platform?
7. Fig 4H,i: it is important to compare NTG and crRNA-13 in wild type animals to confirm the crRNA did not trigger global gene changes.
8. Line 276, the authors should double check the MOI number. MOI represents the ratio of the virus particle number to the cell number.
9. The antisense oligonucleotide targeting the sense repeat-containing transcript failed to bring beneficial effects to patients in the clinical trial. As the Cas13 method still only targets sense repeat, how this could have improved efficacy? This point should be discussed.

Reviewer #2

(Remarks to the Author)
This manuscript shows that RfxCas13 targeting G4C2 repeat-containing RNA for C9-ALS/FTD, which causes amyotrophic lateral sclerosis (ALS) and frontotemporal dementia (FTD), suppresses disease-causing RNAs. Off-target effects of Rfx Cas13 were well shown and high-fidelity variants were tested and provided better targeting with less desirable unwanted effects.

It is the correct methodology to select the crRNA with the highest score with the crRNA screening approach and to choose the one with the lowest undesirable collateral effects. At the same time, testing not only the wild type Rfx Cas13 but also the high-fidelity versions of RfxCas13 in the study also shows a significant effect.
As a noteworthy point in this manuscript, RfxCas13 and its variants have been examined in an extremely comprehensive manner, rather than only their effects on the targeted gene and transcript, given article provide genome-wide effects on the transcriptomes of other genes. In this respect, this study confirms the data of other researchers who will use these enzymes and is a noteworthy point. The data and the shown results are adequate and the interpretations obtained are reasonable. I do not recognize the methodological error (especially related to CRISPR-Cas methodology) and it meets the journal standards and the expected data and figures in the field. In addition, the fact that the publication confirms the Cas13 RNA silencing effects in different cell culture cells as well as in vivo mouse experiments increases the depth, diversity, and persuasive capacity of the publication. There is also enough detail provided for the replication of this study(eg: designed and used crRNA sequences were provided and adequate). This publication provides a perspective and solid basis for the future of gene/transcriptome therapies in human diseases.
However, I do not think that the publication is groundbreaking or very original. Again, the same group published similar publications using similar methodologies, but different diseases were targeted. (eg: Huntington disease (DOI: 10.1126/sciadv.abk2485)). Whereas more studies with different human diseases are necessary for the field and the future of gene/transcriptome therapy approaches for human health.

I will only make some minor suggestions to ensure that the publication is of better quality and also reaches readers outside the field more easily.

1.Major: It should be examined whether Cas13 and its derivatives cause toxicity in the cell types you try. Especially the activity of apoptotic and autophagy genes seen in RNA-Seq is related to the cellular toxicity of those enzymes. For this reason, if the toxicity analyses related to Rfx13 and the variants used-crRNA 13 could be added to the data, the study would be more impressive and the off-target RNA-Seq results would become more meaningful. At the same time, the Cas13 family has a high collateral effect and related toxicity. More gentle enzymes such as Cas7-11, which also targets RNA but does not have a collateral effect, and its smaller variants suitable for AAV packaging, can provide the desired effect with lower damage in the mentioned and similar genetic diseases.

2. Minor: Providing genes and variants in the first main figure is reasonable however location of the designed crRNAs could be provided in the main figure. Relocating the supplementary figure 1a (where crRNA targets) to the main Figure 1 could provide more clarity in terms of both readability and detailing.

3. Minor: Line 296: double "that"

To sum up, I believe the above critics are relatively easy and quick to address and aim to improve the given manuscript.

Reviewer #3

(Remarks to the Author)
In this article, authors McCallister et al. developed a high-fidelity CRISPR-based system using RNA-targeting Cas13 to target G4C2 C9orf72 transcripts. They found that in a mouse model, their targeting system reduced expression of repeat-containing C9 transcripts without affecting overall C9 levels, which in turn led to improvements including reduced RNA foci and reversed transcriptional deficits. In motor neuron-like cells derived from a patient with C9-ALS, their construct showed high specificity and efficient targeting.

Comments:
Overall, this manuscript is outstanding and an important addition to the field. The writing is clear and engaging, the story is easy to follow and logical in its progression, the experiments are strong and technically sound and they illustrate the points made effectively, the technology is really cool and increasingly refined for specificity as the paper progresses, and the authors have made tangible progress towards treating a form of ALS that is proving more challenging than anyone in the field initially expected.

Specific comments:
- Figure 1 is logically described and well diagrammed. It is not clear though what firefly-mediated detection of “collateral effects” truly means – doesn’t it just mean that that particular sequence contained in the Firefly reporter is not targeted by the Cas13? How does that inform regarding the rest of the transcriptome, can the authors elaborate?
- Figure 2: Wondering about the authors’ decision to mix in the KASH vector with their study vector, since being transduced by the one vector would not imply a given cell’s likelihood of having been co-transfected with the vector of interest. Co-labeling with a KASH reporter within the same vector (if it fit) would make sense; but in different vectors, why would these be assumed to be the same cell populations? Unless GFP expression depended on co-transduction, these seem independent events, making this seem like a random sorting, rather than a necessary step. Can the authors provide more rationale?
- Minor: The concluding sentence in the Results section corresponding to Figure 2 goes through 3 different grammatical tenses
- Page 11, a typo: The authors say that “C9-S, the short C9 protein isoform encoded by V3, could not be detected” but C9-S is encoded by V1. Also – throughout the manuscript to this point, the reader wonders what ever happened to V1 – this explanation could occur early in the manuscript, as this is not the first time individual isoforms are measured.
- The RNAseq data is very illustrative and effectively shows the advantages of the high-fidelity effector (N2V8). I do wonder

why the bulk-seq analysis was done in the less-neurologically-relevant HEK cell line rather than SH cells or even the iPSCs; given the neurospecificity of C9 mutations, transcriptomic perturbations in a neuronal line would perhaps make more sense than in HEK.
- Tremendous results in Figure 4 with the correction of DEGs dysregulated in the model. Really striking data that is beautifully analyzed and presented.
- As an aside, the authors' notation in the Discussion that C9 mutations are the most common genetic form of ALS IN SPECIFIC POPULATIONS (and not necessarily universally – because they are under-studied in many other ethnic and racial groups) is noticed and appreciated by this reviewer.
- In Discussion, can the authors comment on the likelihood of immunogenicity with constitutive CRISPR/CAS system expression in humans if this therapy is one day translated?
- Additionally, in Discussion, it is worth mentioning the recent ASO failures in the C9 field (Biogen, WAVE) and why this approach would differ from those, since the target is the same. If this manuscript is more of a proof of principle than an intent to translate, the authors should discuss what the gaps are that need to be filled to translate this to the clinic (e.g., do they feel targeting the antisense strand, or both strands, is the key? Is it something else?) – because at this point, as they themselves point out, several strategies have been tried to knock down C9 repeat-containing transcripts without success when brought to the clinic.
- Somewhere (Materials/Methods or other), authors should describe how mice were chosen for the RNAseq experiment – were these within-litter-balanced to control for between-litter variability?

Best of luck to the authors with this strong manuscript and I look forward to seeing it in print.
Defne Amado

Reviewer #4

(Remarks to the Author)
The objective of this research title "A high-fidelity CRISPR-Cas13 system improves abnormalities associated with C9ORF72-linked ALS/FTD" is to utilize RfxCas13d to specifically target the C9ORF72 gene, carrying hexanucleotide repeat expansion linked to neurodegenerative disorders. This gene has three transcript variants (V1, V2, and V3) generating two protein isoforms (C9-S and C9-L). To address potential pathogenicity, the researchers aimed to selectively silence V1 and V3 while preserving V2. By employing the Cas13 Design Resource, crRNAs were designed to target sequences in exon 1a or intron 1.
A dual-luciferase reporter screen assessed the crRNAs' impact on Renilla luciferase expression (targeting C9ORF72) and collateral effects on firefly luciferase. Among 15 tested crRNAs in HEK 293T cells, crRNAs 13 and 1 effectively reduced Renilla luciferase without notable collateral effects on firefly luciferase. Subsequent tests in HEK293T and SH-SY5Y neuroblastoma cells confirmed that RfxCas13d, in conjunction with crRNAs 13, 7, and 1, selectively decreased V3 mRNA (carrying the hexanucleotide repeat) by 80-95%, minimally impacting the overall transcript pool (all-V). This study underscores the utility of the dual-luciferase reporter screen in identifying effective crRNAs, highlighting RfxCas13d's ability to preferentially target specific transcript variants in human cells.
Additionally, the study explored RfxCas13d's efficacy in targeting the hexanucleotide repeat-containing transcript associated with C9-ALS/FTD in a mouse model (C9-BACexp mice). The researchers employed an AAV vector variant (PHP.eB) for RfxCas13d delivery into the hippocampus and motor cortex, utilizing a dual AAV system with EGFP-KASH to isolate transduced nuclei. Results showed successful transduction, with a high percentage of NeuN-positive cells expressing EGFP-KASH and RfxCas13d. Repeat-targeting crRNAs effectively reduced V3 mRNA without affecting all-V, indicating RfxCas13d's preference for targeting V3 in vivo. FISH analysis demonstrated a significant decrease in RNA foci in treated mice, particularly with crRNAs 13 and 7, indicating the potential of RfxCas13d to impact RNA foci formation in neurons of C9-BACexp mice.
The study further investigated high-fidelity variants of Cas13, specifically RfxCas13d-N2V7 and RfxCas13d-N2V8, for targeting the C9ORF72 gene in HEK293T and SH-SY5Y cells. Both high-fidelity variants effectively decreased V3 mRNA without impacting all-V. RfxCas13d-N2V8 exhibited over 90% reduction in V3 expression with minimal impact on all-V, comparable to native RfxCas13d. Western blot analysis confirmed the approach's selectivity in targeting V3, and RNA-seq analysis demonstrated improved transcriptome-wide specificity for RfxCas13d-N2V8 compared to native RfxCas13d and RfxCas13d-N2V7 when programmed with crRNA-13. The study also highlighted the efficacy of RfxCas13d-N2V8 in reducing V3 mRNA in iPSC-derived motor neuron-like cells from a C9-ALS patient, partially reversing disease-associated transcriptional alterations.
In vivo experiments in a mouse model (C9-BACexp mice) using an AAV-PHP.eB vector for RfxCas13d-N2V8 delivery showcased successful transduction in the hippocampus and motor cortex. FACS-isolated nuclei analysis revealed a significant two-fold decrease in V3 mRNA, confirming preferential targeting of the hexanucleotide repeat-containing RNA by RfxCas13d-N2V8. Importantly, all-V levels remained unaffected, emphasizing the specificity of the targeting. FISH analysis demonstrated a substantial decrease in RNA foci positive for the G4C2 repeat RNA in EGFP-KASH+ cells within the hippocampus and motor cortex, indicating an impact on RNA foci formation. Additionally, RNA-seq analysis revealed that RfxCas13d-N2V8 could partially reverse transcriptional abnormalities associated with the C9-BACexp mice, with a significant reduction in the expression of genes linked to ALS/FTD.
The following are key considerations that should be addressed to enhance the effectiveness of this approach.
Major Concerns:
1. Diverse cell types exhibit distinct expression levels and patterns of C9orf72 isoforms, implying that selective targeting may lead to varied impacts across different cells. The absence of a comprehensive approach encompassing both the central nervous system (CNS) and non-CNS tissues in this manuscript raises the potential for limited applicability. To address this, post-treatment assessments of vital organ tissues, such as the liver and lungs, are recommended.

2. The housing conditions, feeding regimen, and overall environment significantly influence the penetrance of gene expression and, consequently, the disease phenotype in mouse models. The incorporation of behavioral studies would provide valuable insights into potential post-treatment adverse effects, thereby enhancing the overall understanding of the approach's implications.
Minor concerns:
1. The term 'gene silence' is used in several instances. Consider using 'down-regulation' for clarity.

2. Further clarification is needed regarding the firefly luciferase, specifically whether it includes 5' and 3' G4C2 flanking regions. Explain the rationale behind fusing RLuc or FLuc at the 5' end rather than the 3' end, and discuss if the proximity of the crRNA target site to polyA might impact RNA stability.

3. Clarify the choice of 20 repeats of G4C2 which are not pathogenic and explain how the dual reporter system represents heterozygosity of the pathogenic allele. Specify if both reporter plasmids are co-transfected with each crRNA.

4. Offer a rationale for choosing localized administration over intravenous injection of AAV-PHP.eB, despite its proven efficacy when delivered intravenously (PMID: 28671695).

5. In Figure 2d and e, the 'NTG'-treated sample indicates nearly all-V equal to V3, suggesting that V3 contributes almost entirely to all-V. Explain this observation, especially in comparison to crRNA-treated samples where V3 is reduced, implying upregulation of V1 and/or V2.

6. In Figure 2f, clarify whether differences in magnification or cell size contribute to the observation that most RNA foci are cytoplasmic. Address the surprising cytoplasmic localization, which contrasts with expectations (PMID: 26637796). Consider adding an image of RNA foci with nuclear staining for better visualization.

7. Improve clarity and labeling in Figure 3b and c.

8. Figure 3 lacks a control using cells derived from a non-ALS individual.

9. Similar to Figure 2, in Figure 4, most RNA foci are cytoplasmic, contrary to expectations. Consider adding an image of RNA foci with nuclear staining and a negative control for better understanding.

10. Consider conducting an immunoassay of Poly (GP) levels in different brain regions to further support the therapeutic potential of this approach.

Version 1:

Reviewer comments:

Reviewer #1

(Remarks to the Author)
The manuscript has been improved with more data and discussion. One major concern is still the repeat RNA and DPR quantification, which is the foundation of the whole work. Take RNA foci quantification as an example. There is huge background in the cytoplasm according to several FISH images. Some images are better, but there are actually puncta in the cytoplasm when looking carefully, such as Fig. 4e. How to quantify if it is not clear what is the real signal? The concern increases when the GP data does not match the RNA data in mice (Fig. 4c-g). The RNA and RNA foci both reduced significantly in both HPC and MC areas. But it is mentioned in the rebuttal that GP has no change in MC, “which we attribute to the more limited transduction observed in the MC compared to the HPC”. If that’s the case, the RNA level should show the same.
The authors also did not increase the number of iPSC lines. The resources of C9-ALS iPSC lines have increased a lot in recent years and become more accessible to the community (such as Answer ALS). Including a few different lines is common nowadays. And they cannot measure RNA FISH, GP or glutamate-induced toxicity, which are all well established and used in many publications.

Reviewer #2

(Remarks to the Author)
In a revised manuscript number NCOMMS-23-59540. Authors have responded to given comments in an adequate way.
All the given points explained well and necessary experiments at major critics addressed in a sufficient way.
Especially given the comment on Cas7-11 and cellular toxicity not only described in a well established experiment but also provide interesting data to the field.

I don't have additional comments and critiques related to given manuscript.It is sufficient for the publication.

Reviewer #3

(Remarks to the Author)
The authors have adequately addressed all of the concerns that I raised about this manuscript. I particularly note the strengthening of the paper in their inclusion of RNAseq data from more disease-relevant cell lines.

Reviewer #4

(Remarks to the Author)
In the manuscript titled 'A high-fidelity CRISPR-Cas13 system improves abnormalities associated with C9ORF72-linked ALS/FTD', Tristan el al. has proposed a Cas13d-based therapeutic approach. They focused on suppressing C9orf72 transcripts. Using a dual-luciferase reporter screen, effective crRNAs (13, 7, and 1) were identified, reducing V3 mRNA levels by 80-95% with minimal effects on overall transcripts. In vivo experiments in the C9-BACexp mouse model demonstrated successful delivery of RfxCas13d via AAV vectors, leading to significant reduction of V3 mRNA and RNA foci in the hippocampus and motor cortex. The study also investigated high-fidelity variants, specifically RfxCas13d-N2V7 and RfxCas13d-N2V8. RfxCas13d-N2V8 achieved over 90% reduction in V3 expression, showcasing high specificity and minimal off-target effects. This variant also partially reversed transcriptional abnormalities in iPSC-derived motor neuron-like cells and the C9-BACexp mouse model.

In the revised version of manuscript, Tristan el al. has also evaluated potential crRNA with Cas7-11 and addressed the potential concerns indicated in previous version of manuscript. Below are the comments and suggestion on revised manuscripts:

1. The localized administration of AAV appears to limit the broader applicability and efficacy of this therapeutic approach in non-CNS tissues. Could you please clarify how localized injections in the motor cortex and hippocampus offer advantages over systemic delivery methods (such as tail vein or retro-orbital) or intracerebral ventricular (ICV) administration? Any additional data on this would be greatly appreciated.

2. All the crRNAs have been designed against sequences in exon 1a and intron 1, which are present in both the healthy and pathogenic alleles. None of the crRNAs target the G4C2 repeat sequences. As I understand it, the firefly luciferase reporter does not contain the C9orf72 sequence, which is why it is not targeted by any crRNA. The use of this reporter seems less relevant if it does not adequately mimic conditions similar to the healthy allele.

Furthermore, the fusion of RLuc at the 5' UTR of the target sequence (C9orf72) raises concerns about the screening system. Since the target sequences are downstream of RLuc, there may be instances where the expression of RLuc remains unaffected. Additionally, as the authors acknowledged, the proximity of the target sequences to the poly(A) tail could influence the stability and/or availability of crRNA binding sites. This is concerning and could potentially impact the screening outcomes of the crRNAs.

3. In Figure 2f, the G4C2 RNA FISH signal appears diffused and overlapping with EGFP-KASH, making it difficult to clearly identify the RNA foci. Would it be possible to provide higher-quality images that depict more distinguishable differences between the two signals?

4. In Figure 3j, the independent graphs for C9-ALS and wild-type do not allow for a direct comparison between the two groups. Could you please provide a more comparative representation? Additionally, would it be possible to include BMP3 and COL6A5 expression data to better assess any potential off-target effects?

In summary, several of these concerns must be clarified so that the manuscript can convincingly demonstrate the efficacy of the proposed approach as a viable treatment for C9ORF72 neuropathies.

Version 2:

Reviewer comments:

Reviewer #1

(Remarks to the Author)
The concerns have been properly addressed.

Reviewer #4

(Remarks to the Author)
In the revised version of the manuscript, the authors effectively address the comments. They have provided clear explanations and conducted experiments to resolve key concerns. I find no further issues or suggestions for improvement; the manuscript seems appropriate for publication.

Open Access This Peer Review File is licensed under a Creative Commons Attribution 4.0 International License, which permits use, sharing, adaptation, distribution and reproduction in any medium or format, as long as you give appropriate credit to the original author(s) and the source, provide a link to the Creative Commons license, and indicate if changes were made.
In cases where reviewers are anonymous, credit should be given to 'Anonymous Referee' and the source.
The images or other third party material in this Peer Review File are included in the article's Creative Commons license, unless indicated otherwise in a credit line to the material. If material is not included in the article's Creative Commons license and your intended use is not permitted by statutory regulation or exceeds the permitted use, you will need to obtain permission directly from the copyright holder.
To view a copy of this license, visit https://creativecommons.org/licenses/by/4.0/

POINT-BY-POINT RESPONSE
NCOMMS-23-59540

Reviewer #1 (Remarks to the Author):
The hexanucleotide GGGGCC repeat expansion in the C9ORF72 gene is the most common genetic cause of ALS and FTD. One disease mechanism is the toxicity caused by the repeat RNA. Reduction of the repeat containing transcript can potentially reduce the defects. In this manuscript, the authors developed an RNA-targeting CRISPR based platform to reduce the C9ORF72 repeat RNA without affecting the normal C9ORF72 levels, using a Cas13 variant with reduced collateral effects. The method was tested in both cell lines, and patient derived iPS-neurons and mouse models. Overall, the study suggested a new method to decrease the sense repeat RNA as a novel therapeutic strategy. More evidence is required to support the specificity and efficacy of the method.

1. The RNA FISH data are not convincing. In both Fig 2f and 4e, the RNA foci were not visible. It is not clear whether there were real RNA foci compared to negative control (wild type mice). It is also not clear how the foci were quantified. How many cells were counted per biological replicate? In Fig 2g, there were huge variation between replicates. The animal number need to be increased to draw conclusions.

We thank Reviewer 1 for highlighting these critical points. We have updated our manuscript to address these concerns in several ways.

First, we have updated the manuscript to include new panels with an increased number of higher-objective immunofluorescent images that better show the nuclear localization of the RNA foci in C9-BACexp mice. Specifically, in addition to providing higher resolution merge images, we show the individual channels for the G_4C_2 RNA, EGFP-KASH, and DAPI, which altogether better demonstrate the nuclear localization of the RNA foci. These panels are present in **Fig. 2f** and **Fig. 4e**.

Second, to demonstrate that our FISH conditions were selective for the G_4C_2 RNA, we quantified RNA foci in wild-type C57BL/6J mice, measuring only ~0.08 foci per EGFP-KASH$^+$ cell, a ~10-fold difference compared to age-matched C9-BACexp mice (**Supplementary Fig. 7** and **Supplementary Table 2**). This difference is consistent with observations from O'Rourke et al.[1], which reported a ~5- to 8-fold decrease in the percentage of cells positive for the sense foci in C57BL/6J versus C9-BACexp mice.

Third, we have updated the Methods to provide detail on how the RNA foci were quantified. The text on **Pg. 33** now states, "**To quantify foci, Cy3 RNA foci and EGFP-KASH images were merged with DAPI. The number of foci in all EGFP-KASH$^+$ cells were then counted by a blinded investigator, with only nuclear RNA foci counted for this analysis. For each animal, 4-10 random images were analyzed in the HPC and at least 10 random images were analyzed in the MC. The total number of cells analyzed per biological replicate is described in Supplementary Tables 1, 2 and 3. Images were analyzed using ImageJ**." Our methods are consistent with prior studies that analyzed RNA foci in C9-BACexp mice[1,2].

Finally, we have updated the **Supplementary Information** to include three new tables (**Supplementary Tables 1, 2 and 3**) that report the number of cells counted per biological replicate (i.e., per mouse) for each FISH analysis. We note that the number of animals used for this study (n = 4 to 5 for the wild-type RfxCas13d-based study in **Fig. 2** and n = 6 to 7 for the RfxCas13d-N2V8-based study in **Fig. 4**) <u>matches and/or exceeds the number of animals used in prior studies that evaluated various C9ORF72-targeting modalities in the same model</u>. Liu et al.[2], for example, which evaluated an ASO for C9ORF72, used n = 4, Martier et al.[3], which developed a miRNA for C9ORF72, used n = 3, and Bush et al.[4], which tested RNA-targeting small molecules for C9ORF72[4], used n = 4. Further, O'Rourke et al.[1], which established the C9-BACexp mouse model and characterized its RNA foci and their distribution, used n = 3-4.

In total, we counted 467 cells (n = 5 mice), 383 cells (n = 5 mice) and 845 cells (n = 4 mice) for the NTG, crRNA-7 and crRNA-13 groups, respectively, for the measurements in **Fig. 2,** 1,432 (n = 7 mice) and 1,016 (n = 6 mice) for the NTG and crRNA-13 groups, respectively, in the hippocampus for the measurements in **Fig. 4**, and 1,139 (n = 7 mice) and 946 (n = 6 mice) for the NTG and crRNA-13 groups, respectively, in the motor cortex for the measurements in **Fig. 4.** Though past similar studies do not always disclose the total number of cells counted for

their FISH analyses[2,3,5], our counts are similar to those that have reported their methods[6]. Further, our measured values demonstrate similar variability and distribution[6]. Our methods are thus consistent with the field and our study is appropriately designed to support its conclusion.

2. At least three independent iPSC lines (from different individuals) are needed to confirm the effect of the Cas13 platform.

We appreciate the recommendation by Reviewer 1. Our experimental design, however, is consistent with similar studies from the field. Martier et. al.[3], Liu et al. 2021[2], Liu et al. 2022[7], Kandasamy et al.[8], Meijboom et al.[9], and Sachdev et al.[10] all used one iPSC line to evaluate their respective C9ORF72-targeting modalities. Nonetheless, we do appreciate the point raised by Reviewer 1. To avoid overgeneralizing our findings, we have updated the **Discussion** to highlight the limitations of iPSC-based study. We now state on **Pg. 24**, **"…it's important to note the limitations in our iPSC-based study. First, only a single iPSC line from a C9-ALS patient was used. Second, we did not determine if RfxCas13d affected the formation of RNA foci, a DPR protein or any toxic phenotype in the iMNs. Thus, a more detailed study involving: (i) multiple iPSC lines from independent C9-ALS carriers and (ii) an expanded suite of measurements is needed to more generally determine the ability of RfxCas13d to improve functional deficits in iMNs."**

3. Control iPSC lines should also be included, especially for the gene expression rescue experiments (Fig 3k). RNA-seq should be performed on both control and patient neurons to define the disease-linked profile, and then determine whether this profile can be rescued by Cas13. Also whether the Cas13 system also affects the CBLN1 and CBLN2, as well as transcriptome-wide gene expression levels in control cells, need to be tested to confirm the effect is due to the C9 repeat reduction.

We thank Reviewer 1 for raising these important points. First, we have expanded the iPSC-based studies in the manuscript to include measurements in neurospheres derived from a non-ALS individual. As shown in **Fig. 3j**, we measured in these cells a decrease ($P < 0.05$) in the relative V3-to-all-V ratio following their treatment with the PHP.eB-RfxCas13d-N2V8-crRNA-13 vector, which is consistent with our findings in the C9-ALS neurospheres.

Second, we have conducted additional RNA-seq studies to demonstrate a disease-linked profile in the C9-ALS neurospheres. By RNA-seq, we identified 2,470 genes that were dysregulated in the C9-ALS cells (>1.25 FC relative to the non-ALS cells at 14 days *in vitro*; FDR P-value < 0.01; **Supplementary Fig. 10a and Supplementary Data 6 and 7**), with a GO and BP term analysis found to reveal enrichment for numerous functions previously found to be affected in C9-ALS iMNs, including for example synapse organization and neurotransmitter transport[11] (**Supplementary Fig. 10b** and **Supplementary Data 8**).

Third, we have used RNA-seq to measure RfxCas13d targeting specificity in the non-ALS iPS neurons. When paired with crRNA-13, RfxCas13d-N2V8 was found to perturb the expression of only two genes (BMP3 and COL6A5) compared to cells treated with the non-targeted crRNA (>1.25-FC; FDR-adjusted $P < 0.01$; **Supplementary Data 9** and **10)**. Both genes were down-regulated (**Supplementary Data 10**) and are involved in cartilage signaling.

And last, we have conducted additional measurements to bolster the reversion analysis in the iPS neurons. First, CBLN1 mRNA was measured in both non-ALS and C9-ALS neurospheres, which revealed that its expression was increased ~14-fold in the C9-ALS neurospheres, consistent with prior studies[12]. No change in its expression was observed in non-ALS cells following their treatment with the PHP.eB-RfxCas13d-N2V8-crRNA-13 vector ($P > 0.05$; **Fig. 3k**). Further, we have conducted RNA-seq to determine the transcriptome-wide effects of RfxCas13d-N2V8 in the C9-ALS neurospheres. Based on a pairwise analysis with the non-ALS cells, we measured that C9-ALS neurospheres treated with PHP.eB-RfxCas13d-N2V8-crRNA-13 had ~18% fewer DEGs than the C9-ALS cells treated with the non-targeted crRNA (>1.25-FC; FDR-adjusted $P < 0.01$; **Fig. 3l** and **Supplementary Data 11)**. For the DEGs whose expression was found to be normalized following treatment with RfxCas13d-N2V8, a GO term analysis revealed enrichment for functions related to RNA metabolism, including RNA splicing and mRNA splicing, the regulation of RNA and mRNA splicing, and the regulation of mRNA processing (**Supplementary Fig. 12** and **Supplementary Data 12** and **13**). Notably, this effect is smaller than the one

observed in the cortical neurons isolated from C9-BACexp mice treated by RfxCas13d. We elaborate on this in the **Discussion** on Pg. 24, writing, **"…iMNs derived from a C9-ALS patient treated with RfxCas13d-N2V8 and the repeat-targeting crRNA were found to have ~18% fewer DEGs than those treated with the non-targeted crRNA, with a term analysis shown to reveal that the normalized DEGs were enriched for functions associated with RNA metabolism. Notably, however, this normalization was lower than the one observed in C9-BACexp mice, which we attribute in part to the experimental design for each study. For example, FACS was used to isolate transduced nuclei from C9-BACexp mice, whereas the C9-ALS iMNs were analyzed in the bulk and likely contained a greater proportion of non-transduced cells, whose RNA contents contributed to the analysis."**

Interestingly, while performing the experiments for this revision, we reproducibly measured that CBLN2 mRNA was *decreased* in the C9-ALS iPSC neurons when compared to wild-type cells, rather than increased, as expected. This finding is contrary to the expected result[12]. Given this conflicting observation, which could be due to differences in the differentiation protocol and/or the maturity of the cells, CBLN2 was removed from the targeted qPCR analysis in **Fig. 3k**.

4. RNA foci and poly-GP levels should be measured in iPS-neurons. Whether the Cas13 platform can rescue the neuron toxicity phenotypes (such as glutamate induced toxicity) should also be measured.

As recommended by Reviewer 1, we attempted to measure poly(GP) in the iPSC neurons. However, we found that the poly(GP) protein could not be detected at levels above background using a Meso Scale Discovery (MSD) immunoassay platform[2,13-15], potentially due to the limited number of cells per well for this study. However, we have updated the manuscript with new data that demonstrates the ability of RfxCas13d-N2V8 to decrease poly(GP) protein in C9-BACexp mice. Using the same MSD platform, we measured a ~18% decrease in soluble poly(GP) in bulk hippocampal tissue from animals injected with PHP.eB-RfxCas13d-N2V8-crRNA-13 ($P < 0.05$; **Fig. 4g**). No difference in soluble poly(GP) was measured in bulk cortical tissue, however, which we attribute to the more limited transduction observed in the MC compared to the HPC.

RNA FISH and glutamate-induced toxicity were also measured in the iPSC neurons. However, similar to the poly(GP) protein, we were unable to measure signal above background for either analysis. In the case of the RNA FISH, in particular, the clustering of the cells within the neurospheres prevented our imaging of individuals cells for counting. To avoid overgeneralizing our conclusions, we have updated the **Discussion** to highlight the limitations of this study. We state on **Pg. 24**, "**it's important to note the limitations in our iPSC-based study. First, only a single iPSC line from a C9-ALS patient was used. Second, we did not determine if RfxCas13d affected the formation of RNA foci, a DPR protein or any toxic phenotype in the iMNs. Thus, a more detailed study involving: (i) multiple iPSC lines from independent C9-ALS carriers and (ii) an expanded suite of measurements is needed to more generally determine the ability of RfxCas13d to improve functional deficits in iMNs."** Nevertheless, our study provides compelling evidence to support the effect of RfxCas13d. We find that it not only reduced RNA foci and a DPR protein in C9-BACexp mice, but that it reversed a percentage of the transcriptional deficits that we found manifest in these mice.

5. The antisense RNA levels should be measured by RT-qPCR and RNA FISH to test whether this method influences the antisense transcripts.

The manuscript has been updated to include measurements of the antisense RNA, both in C9-ALS and wild-type iPS neurons and C9-BACexp mice. In all cell types, no change in its expression was observed following treatment with the PHP.eB-RfxCas13d-N2V8-crRNA-13 vector compared to the controls ($P > 0.05$ for C9-ALS and wild-type neurospheres; **Supplementary Fig. 11**; $P > 0.05$ for HPC and MC in C9-BACexp mice; **Supplementary Fig. 16**). Given these data, which demonstrate that suppressing the G_4C_2 repeat RNA with RfxCas13d did not influence the antisense RNA, FISH was not conducted.

6. For mouse studies, any disease or pathological phenotypes can be rescued by the CRISPR platform?

Though C9-BACexp mice do not manifest overt motor deficits, we found that RfxCas13d improved multiple phenotypes in these animals. As summarized in the **Discussion** on **Pg. 24**, both RfxCas13d and RfxCas13d-N2V8

reduced the number of RNA foci, while RfxCas13d-N2V8 decreased the DPR protein poly(GP). Further, we found by RNA-seq that RfxCas13d-N2V8 reversed a percentage of the transcriptional abnormalities that developed in C9-BACexp mice. Thus, RfxCas13d improved several of the pathological phenotypes that manifest in C9-BACexp mice.

7. Fig 4H,i: it is important to compare NTG and crRNA-13 in wild type animals to confirm the crRNA did not trigger global gene changes.

We thank Reviewer 1 for the recommendation. We have updated the manuscript to determine if RfxCas13d-N2V8 with crRNA-13 triggered global genes changes in wild-type mice. By a pairwise comparison between C57BL/6J mice injected with PHP.eB-RfxCas13d-N2V8-crRNA-13 and PHP.eB-RfxCas13d-N2V8-NTG, we identified 84 genes that were differentially expressed in mice injected with PHP.eB-RfxCas13d-N2V8-crRNA-13 (>1.25-FC; FDR-adjusted $P < 0.01$; **Supplementary Data 17**), 64 of which were up-regulated and 20 of which were down-regulated (**Supplementary Data 18**). Interestingly, the DEGs were primarily associated with immune response-related terms, including cellular responses to interferons and host defense responses ($P < 0.01$; **Supplementary Data 19**), which we now elaborate on in the **Discussion**.

8. Line 276, the authors should double check the MOI number. MOI represents the ratio of the virus particle number to the cell number.

An MOI of $\sim 10^6$ was in fact used for the iPSC-based studies. Multiple different AAV serotypes transduce cells in culture most effectively[16] with MOIs of $\sim 10^5$-10^6, with neurons among the cell types often found to require high MOIs for delivery[17]. Our methods are consistent with the literature.

9. The antisense oligonucleotide targeting the sense repeat-containing transcript failed to bring beneficial effects to patients in the clinical trial. As the Cas13 method still only targets sense repeat, how this could have improved efficacy? This point should be discussed.

We thank Reviewer 1 for this recommendation. Based on their comments and the recommendation by Reviewer 3, we have updated the text to discuss these trials and to speculate on how RfxCas13d can be used to improve and/or expand on past approaches. We state on **Pgs. 24-25**, "**ASOs for the G_4C_2 repeat RNA developed by Wave Life Sciences (ClinicalTrials.gov NCT04931862) and Ionis Pharmaceuticals and Biogen (ClinicalTrials.gov: NCT04288856) failed to show a benefit in human trials. Though the exact reason for this remains unknown, these ASOs targeted only the G_4C_2 (sense) repeat RNA and were thus presumed to have no effect on the G_2C_4 (antisense) transcript, which is hypothesized to contribute to disease pathogenesis[18-21]. Similar to these ASOs, the approach developed here only targeted the G_4C_2 repeat RNA. However, RfxCas13d possesses certain advantages to ASOs that could expand opportunities for C9-ALS/FTD. For instance, RfxCas13d is genetically encodable. It thus has the theoretical ability to be continuously expressed in non-dividing cells to sustain an effect. In the case of C9-ALS/FTD, this could provide a means to provide durable neuroprotection from the presumed toxicity of the G_4C_2 repeat RNA in pre-symptomatic carriers or individuals at the earliest stage(s) of disease. To this point, an ASO targeting the G_4C_2 repeat RNA was found to prevent the emergence of certain toxic signatures in iPSC-derived neurons from C9-ALS patients[69]. Additionally, RfxCas13d is a multiplexable enzyme[22]. Thus, it has the capacity to simultaneously target both the G_4C_2 (sense) and G_2C_4 (antisense) repeat RNAs from a single vector formulation. Though not explored here, our proof-of-concept nonetheless sets the stage for the development such dual-targeting systems, which could offer benefit to post-symptomatic individuals**."

Reviewer #2 (Remarks to the Author):
This manuscript shows that RfxCas13 targeting G_4C_2 repeat-containing RNA for C9-ALS/FTD, which causes amyotrophic lateral sclerosis (ALS) and frontotemporal dementia (FTD), suppresses disease-causing RNAs. Off-target effects of Rfx Cas13 were well shown and high-fidelity variants were tested and provided better targeting with less desirable unwanted effects.

It is the correct methodology to select the crRNA with the highest score with the crRNA screening approach and to choose the one with the lowest undesirable collateral effects. At the same time, testing not only the wild type

RfxCas13 but also the high-fidelity versions of RfxCas13 in the study also shows a significant effect. As a noteworthy point in this manuscript, RfxCas13 and its variants have been examined in an extremely comprehensive manner, rather than only their effects on the targeted gene and transcript, given article provide genome-wide effects on the transcriptomes of other genes. In this respect, this study confirms the data of other researchers who will use these enzymes and is a noteworthy point. The data and the shown results are adequate and the interpretations obtained are reasonable. I do not recognize the methodological error (especially related to CRISPR-Cas methodology) and it meets the journal standards and the expected data and figures in the field. In addition, the fact that the publication confirms the Cas13 RNA silencing effects in different cell culture cells as well as in vivo mouse experiments increases the depth, diversity, and persuasive capacity of the publication. There is also enough detail provided for the replication of this study (eg: designed and used crRNA sequences were provided and adequate). This publication provides a perspective and solid basis for the future of gene/transcriptome therapies in human diseases.

However, I do not think that the publication is groundbreaking or very original. Again, the same group published similar publications using similar methodologies, but different diseases were targeted. (eg: Huntington disease (DOI: 10.1126/sciadv.abk2485)). Whereas more studies with different human diseases are necessary for the field and the future of gene/transcriptome therapy approaches for human health.

I will only make some minor suggestions to ensure that the publication is of better quality and also reaches readers outside the field more easily.

We thank Reviewer 2 for their assessment. Though we previously demonstrated that Cas13 could be delivered to the nervous system, this technology has not been used to target C9ORF72 in any published study. Our manuscript is therefore novel and has potentially high significance, as C9-ALS/FTD is a debilitating disorder with limited treatment options.

1.Major: It should be examined whether Cas13 and its derivatives cause toxicity in the cell types you try. Especially the activity of apoptotic and autophagy genes seen in RNA-Seq is related to the cellular toxicity of those enzymes. For this reason, if the toxicity analyses related to Rfx13 and the variants used-crRNA 13 could be added to the data, the study would be more impressive and the off-target RNA-Seq results would become more meaningful. At the same time, the Cas13 family has a high collateral effect and related toxicity. More gentle enzymes such as Cas7-11, which also targets RNA but does not have a collateral effect, and its smaller variants suitable for AAV packaging, can provide the desired effect with lower damage in the mentioned and similar genetic diseases.

We thank Reviewer 2 for these recommendations. To determine the toxicity of RfxCas13d, propidium iodide was used to measure the viability of HEK293T cells and SH-SY5Y cells following their transfection with RfxCas13d, RfxCas13d-N2V7 or RfxCas13d-N2V8 with crRNA-13. As described on **Pg. 12**, we observed a modest but measurable decrease in viability in cells transfected with RfxCas13d and crRNA-13 ($P < 0.05$ for both; **Supplementary Fig. 8**), however, no decrease in viability was observed in either cell type following their transfection with RfxCas13d-N2V8 or RfxCas13d-N2V7 ($P > 0.05$ for all; **Supplementary Fig. 8**). These data complement our RNA-seq studies and provide a more meaningful examination of the *in vitro* tolerability of RfxCas13d.

Second, we have updated our manuscript to determine the applicability of DiCas7-11[23], a subtype III-E effector from *Desulfonema ishimotonii* that can mediate RNA knockdown in mammalian cells but with reportedly limited collateral effects, for C9-ALS/FTD. HEK293T cells were co-transfected with our dual-luciferase reporter system and an expression vector encoding DiCas7-11 alongside one of 15 crRNAs designed to target the equivalent positions in C9ORF72 as the crRNAs for RfxCas13d (**Supplementary Fig. 1** and **Supplementary Fig. 2a**). In total, only eight crRNAs for DiCas7-11 were found to significantly decrease Renilla expression ($P < 0.05$; **Supplementary Fig. 2b**), with the most efficient of these, crRNA-2, found to decrease it by only ~45% ($P < 0.001$; **Supplementary Fig. 2b)**. Surprisingly, eleven of the fifteen crRNAs decreased firefly luciferase by at least 20% relative to the non-targeted control ($P < 0.05$ for all eleven; **Supplementary Fig. 2b**), implying non-specific

activity. We note on **Pg. 7** that, due to its improved ability to target C9ORF72 based on our dual-reporter screen, RfxCas13d was utilized for our studies.

Given the surprising nature of these results, we have updated the Discussion to elaborate on them. We write on **Pg. 23**, "**Because of its ability to mediate RNA knockdown with limited collateral effects, we also evaluated whether DiCas7-11[23] could target C9ORF72. To our surprise, DiCas7-11 was not only less efficient than RfxCas13d at decreasing Renilla luciferase expression but the majority of its crRNAs also reduced the expression of firefly luciferase – our reporter for collateral effects – implying non-specific activity. Given the lack of computational tools for predicting functional crRNAs for DiCas7-11, we designed its crRNAs to target the equivalent sites in C9ORF72 as the crRNAs for RfxCas13d. However, as our findings indicate that these crRNAs are likely sub-optimal, a more comprehensive screen involving the tiling of C9ORF72 with crRNAs for DiCas7-11 would be more instructive in determining its applicability for C9-ALS/FTD.**"

2. Minor: Providing genes and variants in the first main figure is reasonable however location of the designed crRNAs could be provided in the main figure. Relocating the supplementary figure 1a (where crRNA targets) to the main Figure 1 could provide more clarity in terms of both readability and detailing.

Fig. 1a has been updated to include a new cartoon that includes the targeted sequence from exon 1a and intron 1 of C9ORF72 and the binding sites for the crRNAs for RfxCas13d. Additionally, **Supplementary Fig. 1** has been updated to indicate the sequences targeted by RfxCas13d and DiCas7-11.

3. Minor: Line 296: double "that"

We thank Reviewer 2 for catching this typo.

To sum up, I believe the above critics are relatively easy and quick to address and aim to improve the given manuscript.

We thank Reviewer 2 for their recommendations, which have increased the depth and significance of our study.

Reviewer #3 (Remarks to the Author):
In this article, authors McCallister et al. developed a high-fidelity CRISPR-based system using RNA-targeting Cas13 to target G_4C_2 C9orf72 transcripts. They found that in a mouse model, their targeting system reduced expression of repeat-containing C9 transcripts without affecting overall C9 levels, which in turn led to improvements including reduced RNA foci and reversed transcriptional deficits. In motor neuron-like cells derived from a patient with C9-ALS, their construct showed high specificity and efficient targeting.

Comments:
Overall, this manuscript is outstanding and an important addition to the field. The writing is clear and engaging, the story is easy to follow and logical in its progression, the experiments are strong and technically sound and they illustrate the points made effectively, the technology is really cool and increasingly refined for specificity as the paper progresses, and the authors have made tangible progress towards treating a form of ALS that is proving more challenging than anyone in the field initially expected.

Specific comments:
- Figure 1 is logically described and well diagrammed. It is not clear though what firefly-mediated detection of "collateral effects" truly means – doesn't it just mean that that particular sequence contained in the Firefly reporter is not targeted by the Cas13? How does that inform regarding the rest of the transcriptome, can the authors elaborate?

We have updated the manuscript on **Pg. 6** to better describe the meaning of collateral effects and the mechanism underlying this assay. The text now states, "**Recognition of a target RNA by RfxCas13d unlocks its intrinsic ability to indiscriminately *trans*-cleave non-specific bystander RNAs[24-30], which in turn can lead to off-target effects. Given this, we incorporated into our screen a second reporter to monitor collateral effects (i.e., the degradation of non-target RNAs). As the extent of *trans*-cleavage can depend on the crRNA[31,32], we reasoned that utilizing a dedicated reporter for *trans*-cleavage could aid in the identification of crRNAs that minimize this effect and thus potentially induce fewer off-target effects across the transcriptome[29]. For this,**

we used firefly luciferase (Fig. 1c), which was not targeted by any crRNA but whose expression was nonetheless expected to decrease if collaterally *trans*-cleaved by RfxCas13d."

Increased collateral cleavage of a surrogate reporter has been found to correlate with off-target effects on a transcriptome-wide scale[29]. We indicate this now on **Pg. 6** as a potential benefit for considering collateral effects in a screen.

- Figure 2: Wondering about the authors' decision to mix in the KASH vector with their study vector, since being transduced by the one vector would not imply a given cell's likelihood of having been co-transfected with the vector of interest. Co-labeling with a KASH reporter within the same vector (if it fit) would make sense; but in different vectors, why would these be assumed to be the same cell populations? Unless GFP expression depended on co-transduction, these seem independent events, making this seem like a random sorting, rather than a necessary step. Can the authors provide more rationale?

We thank Reviewer 3 for raising this point and for the opportunity to clarify. We have updated the text on **Pgs. 9-10** to better explain the rationale behind this strategy. We now write, "**Notably, as its presence in the RfxCas13d-encoding AAV vector would cause it to exceed its carrying capacity, EGFP-KASH was delivered by a second AAV. However, because a proportion of cells can be transduced by multiple AAV vector genomes[33,34], dual-vector-based approaches such as this one can nonetheless enrich for nuclei transduced by the first vector[35-37].**" Our immunofluorescence-based analysis reinforces this point, as ~72% and ~75% of the EGFP-KASH+ cells detected in the hippocampus and motor cortex, respectively, were found to be positive for RfxCas13d by its hemagglutinin (HA) epitope (data shown in **Supplementary Fig. 5**).

- Minor: The concluding sentence in the Results section corresponding to Figure 2 goes through 3 different grammatical tenses

We thank Reviewer 3 for bringing this to our attention. The sentence has been revised for a single tense.

- Page 11, a typo: The authors say that "C9-S, the short C9 protein isoform encoded by V3, could not be detected" but C9-S is encoded by V1.

The text on Pg. 13 has been updated to state, "**C9-S, the short C9ORF72 protein isoform encoded by V1, could not be detected, and thus was not analyzed**." We apologize for the error.

Also – throughout the manuscript to this point, the reader wonders what ever happened to V1 – this explanation could occur early in the manuscript, as this is not the first time individual isoforms are measured.

Reviewer 3 is correct. Rather than measuring both V3 and V1, we use V3 a proxy for the G_4C_2 repeat RNA, which we initially indicate on **Pg. 7**. To remind the reader of this point, we now re-emphasize it on **Pgs. 10** and **17.**

- The RNAseq data is very illustrative and effectively shows the advantages of the high-fidelity effector (N2V8). I do wonder why the bulk-seq analysis was done in the less-neurologically-relevant HEK cell line rather than SH cells or even the iPSCs; given the neurospecificity of C9 mutations, transcriptomic perturbations in a neuronal line would perhaps make more sense than in HEK.

This RNA-seq analysis was originally done in HEK293T cells given their transfectability and workhouse nature in our laboratory. Reviewer 3, however, raises an excellent point. As such, we have expanded the scope of our RNA-seq studies and now use it to interrogate the specificity of RfxCas13d-N2V8 in two additional cell types: (i) induced motor neuron-like cells from a C9-ALS patient and (ii) enriched cortical neurons from C57BL/6J mice. In both cases, a relatively limited number of off-target effects were measured, though we did observe an enrichment for several immune-related functions in cells from C9-BACexp mice, which we elaborate on in the **Discussion**.

For the RNA-seq analysis in the motor neuron-like cells, we write on **Pg. 15**, "**RNA-seq was next used to determine the transcriptome-wide specificity of RfxCas13d-N2V8 in the wild-type neurospheres at 14 days post-transduction. When paired with crRNA-13, RfxCas13d-N2V8 was found to perturb the expression of**

only two genes (BMP3 and COL6A5; >1.25-FC to non-targeted crRNA; FDR-adjusted $P < 0.01$; Supplementary Data 9 and 10), both of which were down-regulated. BMP3 and COL6A5 are both involved in cartilage signaling."

For the RNA-seq analysis in C57BL/6J mice, we write on **Pg. 20**, "**Last for this analysis, we determined the transcriptome-wide specificity of RfxCas13d-N2V8 *in vivo*. Through a comparison between C57BL/6J mice injected between PHP.eB-RfxCas13d-N2V8-crRNA-13 and PHP.eB-RfxCas13d-N2V8-NTG, we identified 84 genes that were differentially expressed by crRNA-13 (>1.25-FC; FDR-adjusted $P < 0.01$; Supplementary Data 17), 64 of which were up-regulated and 20 of which were down-regulated (Supplementary Data 18). Interestingly, the DEGs were found to associate with immune response-related terms, including cellular responses to interferons and host defense responses ($P < 0.01$; Supplementary Data 19).**"

- Tremendous results in Figure 4 with the correction of DEGs dysregulated in the model. Really striking data that is beautifully analyzed and presented.

We thank the Reviewer for the kind words.

- As an aside, the authors' notation in the Discussion that C9 mutations are the most common genetic form of ALS IN SPECIFIC POPULATIONS (and not necessarily universally – because they are under-studied in many other ethnic and racial groups) is noticed and appreciated by this reviewer.

- In Discussion, can the authors comment on the likelihood of immunogenicity with constitutive CRISPR/CAS system expression in humans if this therapy is one day translated?

We thank Reviewer 3 for raising this very important point. We have revised the Discussion to better elaborate on this concern and highlight the apparent enrichment in immune-related functions observed among the DEGs induced by RfxCas13d-N2V8 and its crRNA in C57BL/6J mice. We now write on **Pgs. 25-26**, "**…Additionally, RfxCas13d, like other CRISPR effector proteins, can be recognized as foreign by the immune system and elicit an immunological response, which could pose a challenge for its clinical implementation, particularly given its need to be continuously expressed. Notably, among the DEGs induced by RfxCas13d-N2V8 and the repeat-targeting crRNA in C57BL/6J mice, we observed enrichment for terms related to immune function and/or cellular defense, underscoring its potential immunogenicity and the importance for implementing strategies for controlling and/or preventing these effects.**"

- Additionally, in Discussion, it is worth mentioning the recent ASO failures in the C9 field (Biogen, WAVE) and why this approach would differ from those, since the target is the same. If this manuscript is more of a proof of principle than an intent to translate, the authors should discuss what the gaps are that need to be filled to translate this to the clinic (e.g., do they feel targeting the antisense strand, or both strands, is the key? Is it something else?) – because at this point, as they themselves point out, several strategies have been tried to knock down C9 repeat-containing transcripts without success when brought to the clinic.

We appreciate the recommendation by Reviewer 3. Given that Reviewer 1 raised a similar point, we have updated the Discussion on **Pgs. 24-25** to not only mention the results of the clinical trials for the ASOs for the G_4C_2 repeat RNA, but to elaborate on the potential opportunities afforded by an RfxCas13d-based approach for C9-ALS/FTD. Additionally, as our study is a proof-of-concept, we offer a forward-looking perspective on its application for C9-ALS/FTD, including its potential for dual-targeting the sense and antisense RNA.

We state on **Pgs. 24-25**, "**ASOs for the G_4C_2 repeat RNA developed by Wave Life Sciences (ClinicalTrials.gov NCT04931862) and Ionis Pharmaceuticals and Biogen (ClinicalTrials.gov: NCT04288856) failed to show a benefit in human trials. Though the exact reason for this remains unknown, these ASOs targeted only the G_4C_2 (sense) repeat RNA and were thus presumed to have no effect on the G_2C_4 (antisense) transcript, which is hypothesized to contribute to disease pathogenesis[18-21]. Similar to these ASOs, the approach developed in this study only targeted the G_4C_2 repeat RNA. However, RfxCas13d possesses certain advantages to ASOs that could expand opportunities for C9-ALS/FTD. For instance, RfxCas13d is**

genetically encodable. It thus has the theoretical ability to be continuously expressed in non-dividing cells to sustain an effect. In the case of C9-ALS/FTD, this could provide a means to provide durable neuroprotection from the presumed toxicity of the G_4C_2 repeat RNA in pre-symptomatic carriers or individuals at the earliest stage(s) of disease. To this point, an ASO targeting the G_4C_2 repeat RNA was found to prevent the emergence of certain toxic signatures in iPSC-derived neurons from C9-ALS patients[69]. Additionally, RfxCas13d is a multiplexable enzyme[22]. Thus, it has the capacity to simultaneously target both the G_4C_2 (sense) and G_2C_4 (antisense) repeat RNAs from a single vector formulation. Though not explored here, our proof-of-concept nonetheless sets the stage for the development such dual-targeting systems, which could offer benefit to post-symptomatic individuals."

- Somewhere (Materials/Methods or other), authors should describe how mice were chosen for the RNAseq experiment – were these within-litter-balanced to control for between-litter variability?

The C57BL/6J mice used in the RNA-seq study were within-litter-balanced with the C9-BACexp mice. This is indicated in the **Results** and also the legend for **Fig. 4**.

Best of luck to the authors with this strong manuscript and I look forward to seeing it in print.
Defne Amado

We thank the Reviewer for their recommendations, which have improved our manuscript.

Reviewer #4 (Remarks to the Author):
The objective of this research title "A high-fidelity CRISPR-Cas13 system improves abnormalities associated with C9ORF72-linked ALS/FTD" is to utilize RfxCas13d to specifically target the C9ORF72 gene, carrying hexanucleotide repeat expansion linked to neurodegenerative disorders. This gene has three transcript variants (V1, V2, and V3) generating two protein isoforms (C9-S and C9-L). To address potential pathogenicity, the researchers aimed to selectively silence V1 and V3 while preserving V2. By employing the Cas13 Design Resource, crRNAs were designed to target sequences in exon 1a or intron 1.

A dual-luciferase reporter screen assessed the crRNAs' impact on Renilla luciferase expression (targeting C9ORF72) and collateral effects on firefly luciferase. Among 15 tested crRNAs in HEK 293T cells, crRNAs 13 and 1 effectively reduced Renilla luciferase without notable collateral effects on firefly luciferase. Subsequent tests in HEK293T and SH-SY5Y neuroblastoma cells confirmed that RfxCas13d, in conjunction with crRNAs 13, 7, and 1, selectively decreased V3 mRNA (carrying the hexanucleotide repeat) by 80-95%, minimally impacting the overall transcript pool (all-V). This study underscores the utility of the dual-luciferase reporter screen in identifying effective crRNAs, highlighting RfxCas13d's ability to preferentially target specific transcript variants in human cells.

Additionally, the study explored RfxCas13d's efficacy in targeting the hexanucleotide repeat-containing transcript associated with C9-ALS/FTD in a mouse model (C9-BACexp mice). The researchers employed an AAV vector variant (PHP.eB) for RfxCas13d delivery into the hippocampus and motor cortex, utilizing a dual AAV system with EGFP-KASH to isolate transduced nuclei. Results showed successful transduction, with a high percentage of NeuN-positive cells expressing EGFP-KASH and RfxCas13d. Repeat-targeting crRNAs effectively reduced V3 mRNA without affecting all-V, indicating RfxCas13d's preference for targeting V3 in vivo. FISH analysis demonstrated a significant decrease in RNA foci in treated mice, particularly with crRNAs 13 and 7, indicating the potential of RfxCas13d to impact RNA foci formation in neurons of C9-BACexp mice.

The study further investigated high-fidelity variants of Cas13, specifically RfxCas13d-N2V7 and RfxCas13d-N2V8, for targeting the C9ORF72 gene in HEK293T and SH-SY5Y cells. Both high-fidelity variants effectively decreased V3 mRNA without impacting all-V. RfxCas13d-N2V8 exhibited over 90% reduction in V3 expression with minimal impact on all-V, comparable to native RfxCas13d. Western blot analysis confirmed the approach's selectivity in targeting V3, and RNA-seq analysis demonstrated improved transcriptome-wide specificity for RfxCas13d-N2V8 compared to native RfxCas13d and RfxCas13d-N2V7 when programmed with crRNA-13. The

study also highlighted the efficacy of RfxCas13d-N2V8 in reducing V3 mRNA in iPSC-derived motor neuron-like cells from a C9-ALS patient, partially reversing disease-associated transcriptional alterations.

In vivo experiments in a mouse model (C9-BACexp mice) using an AAV-PHP.eB vector for RfxCas13d-N2V8 delivery showcased successful transduction in the hippocampus and motor cortex. FACS-isolated nuclei analysis revealed a significant two-fold decrease in V3 mRNA, confirming preferential targeting of the hexanucleotide repeat-containing RNA by RfxCas13d-N2V8. Importantly, all-V levels remained unaffected, emphasizing the specificity of the targeting. FISH analysis demonstrated a substantial decrease in RNA foci positive for the G4C2 repeat RNA in EGFP-KASH+ cells within the hippocampus and motor cortex, indicating an impact on RNA foci formation. Additionally, RNA-seq analysis revealed that RfxCas13d-N2V8 could partially reverse transcriptional abnormalities associated with the C9-BACexp mice, with a significant reduction in the expression of genes linked to ALS/FTD.

The following are key considerations that should be addressed to enhance the effectiveness of this approach.

Major Concerns:

1. Diverse cell types exhibit distinct expression levels and patterns of C9orf72 isoforms, implying that selective targeting may lead to varied impacts across different cells. The absence of a comprehensive approach encompassing both the central nervous system (CNS) and non-CNS tissues in this manuscript raises the potential for limited applicability. To address this, post-treatment assessments of vital organ tissues, such as the liver and lungs, are recommended.

Per the recommendation of Reviewer 4, we have updated our manuscript to include measurements of the distribution of the PHP.eB vector and the expression of V3, all-V and the antisense RNA in non-CNS tissues, which includes the heart, lungs, liver, kidney, and spleen. Based on qPCR, between ~1.1 to ~2.6 vector genomes per diploid genome (vg/dg) were measured in the spinal cord, heart, lungs, liver, kidney, and spleen of C9-BACexp mice injected with PHP.eB-RfxCas13d-N2V8-crRNA-13 and PHP.eB-RfxCas13d-N2V8-NTG (**Supplementary Fig. 19**), while ~114 and ~152 vg/dg were measured in the HPC and ~25 and ~22 vg/dg were measured in the MC of the same mice (**Supplementary Fig. 19**). No difference in V3, all-V or the antisense RNA was measured in the spinal cord, heart, lung, liver, kidney and spleen of these animals ($P > 0.05$ for all; **Supplementary Fig. 20**).

2. The housing conditions, feeding regimen, and overall environment significantly influence the penetrance of gene expression and, consequently, the disease phenotype in mouse models. The incorporation of behavioral studies would provide valuable insights into potential post-treatment adverse effects, thereby enhancing the overall understanding of the approach's implications.

To address this important point, body weight, motor coordination, and hindlimb and forelimb grip strength were measured for two months in C9-BACexp mice injected with PHP.eB-RfxCas13d-N2V8-crRNA-13 or PHP.eB-RfxCas13d-N2V8-NTG. No deficits were observed for any assessment in the animals treated with RfxCas13d-N2V8-crRNA-13 versus the non-targeted crRNA (**Supplementary Fig. 18**).

Though these findings indicate no discernable adverse effects, we discuss the need for additional studies to more compressively determine the tolerability of this approach. We write on **Pg. 26**, "…**no discernable functional deficits were observed in C9-BACexp mice injected with RfxCas13d-N2V8 and the repeat-targeting crRNA. However, a more comprehensive and longer-term study in mice carrying the normal C9ORF72 allele will be needed to more definitively determine the tolerability of this approach**."

Other concerns:

1. The term 'gene silence' is used in several instances. Consider using 'down-regulation' for clarity.

We thank Reviewer 4 for the recommendation. "Gene silencing" has been replaced with "down-regulation," "lowering," and "suppression" or other similar terms.

2. Further clarification is needed regarding the firefly luciferase, specifically whether it includes 5' and 3' G4C2 flanking regions. Explain the rationale behind fusing RLuc or FLuc at the 5' end rather than the 3' end, and discuss if the proximity of the crRNA target site to polyA might impact RNA stability.

The manuscript has been updated to better explain the structure of the firefly luciferase reporter. We now state on **Pg. 6**, "**…we used firefly luciferase (Fig. 1c), which was not targeted by any crRNA but whose expression was nonetheless expected to decrease if collaterally *trans*-cleaved by RfxCas13d.**" As indicated in the main-text and in **Fig. 1**, only Renilla luciferase, whose 3' UTR was modified to contain a region of C9ORF72, was targeted by the crRNAs.

We also elaborate on the rationale for fusing C9ORF72 to the 3' UTR of Renilla luciferase, noting that our approach was inspired by a prior study. We write on Pg. 5, "**This design was inspired by a study that fused a region of C9ORF72 to the 3' UTR of Renilla to evaluate C9ORF72-targeting microRNAs[38]**."

Finally, we updated the **Discussion** to speculate on the effects of fusing C9ORF72 to the 3' UTR of Renilla, noting, **"…as C9ORF72 was fused to the 3' UTR of Renilla luciferase, we note that its proximity to its poly(A) signal sequence could influence its stability and/or the availability of certain crRNA binding sites. Thus, in the future, it may be more practical to screen crRNAs against the endogenous C9ORF72 transcript variants in order to avoid potential confounding factor(s) associated with the utilization of a reporter."**

3. Clarify that choice of 20 repeats of G4C2 which are not pathogenic and explain how the dual reporter system represents heterozygosity of the pathogenic allele. Specify if both reporter plasmids are co-transfected with each crRNA.

The text on **Pg. 5** has been updated to indicate that 20 copies of the G_4C_2 repeat were used as it was the longest length that could be commercially synthesized.

In addition, heterozygosity of the pathogenic allele was not considered by our reporter, which is a limitation of the approach. To indicate this, we updated the **Discussion** on **Pg. 23** to state, **"…targeting the endogenous C9ORF72 transcript variants in the context of the repeat expansion would enable the heterozygosity of the disorder to be considered within the screen."**

4. Offer rationale for choosing localized administration over intravenous injection of AAV-PHP.eB, despite its proven efficacy when delivered intravenously (PMID: 28671695).

The text on **Pg. 9** has been updated to better explain our rationale for using local delivery rather than systemic for PHP.eB. We write, **"To maximize delivery, we chose to inject RfxCas13d directly to the motor cortex (MC) and hippocampus (HPC), two regions of the brain in C9-BACexp mice that form RNA foci comprised of the G_4C_2 repeat RNA and produce measurable quantities of the DPR protein poly(GP)[1]. Though most commonly administered intravenously, we used the AAV vector variant PHP.eB[33] to deliver RfxCas13d, as it can transduce cells to a similar extent as AAV9 when injected locally to the brain[39] but, in the experience of our laboratory, packages at a higher titer."**

5. In Figure 2f, clarify whether differences in magnification or cell size contribute to the observation that most RNA foci are cytoplasmic. Address the surprising cytoplasmic localization, which contrasts with expectations (PMID: 26637796). Consider adding an image of RNA foci with nuclear staining for better visualization.

As discussed for Reviewer 1, we have updated the manuscript to include new panels with an increased number of higher-objective immunofluorescent images that better show the nuclear localization of the RNA foci in C9-BACexp mice. In particular, in addition to providing higher resolution merge images, these panels show the individual channels for the G_4C_2 RNA, EGFP-KASH, and DAPI, which altogether better demonstrate the nuclear localization of the RNA foci. These panels are present in **Fig. 2f** and **Fig. 4e**.

6. Improve clarity and labeling in Figure 3b and c. The blue and white colors are reversed for All-V and V3.

We thank Reviewer 4 for identifying this error. We have corrected the blue and white colors for legends in **Fig. 3b** and **3c**.

7. Figure 3 lacks a control using cells derived from a non-ALS individual.

As discussed for Reviewer 1, we have expanded the iPSC-based studies in the manuscript to include measurements for neurospheres derived from a non-ALS individual. As shown in **Fig. 3j**, we measured in these cells a decrease ($P < 0.05$) in the relative V3-to-all-V ratio following their treatment with RfxCas13d-N2V8, which is consistent with our findings in the C9-ALS neurospheres.

We have also used these cells to conduct additional RNA-seq studies to demonstrate a disease-linked profile in the C9-ALS neurospheres, identifying 2,470 genes that were dysregulated in the C9-ALS cells (>1.25 FC relative to the non-ALS cells at 14 days *in vitro*; FDR P-value < 0.01; **Supplementary Fig. 10a and Supplementary Data 6 and 7**). Further, we used these cells to determine RfxCas13d targeting specificity. We find that, when paired with crRNA-13, RfxCas13d-N2V8 perturbed the expression of only two genes (BMP3 and COL6A5) compared to cells treated with the non-targeted crRNA (>1.25-FC; FDR-adjusted $P < 0.01$; **Supplementary Data 9** and **10)**. Both genes were down-regulated (**Supplementary Data 10**) and are involved in cartilage signaling.

Last, we used cells from the non-ALS individual to bolster the reversion analysis in the iPS neurons (**Fig. 3l** and **Supplementary Data 11**), including RNA-seq, which was conducted to determine the transcriptome-wide effects of RfxCas13d-N2V8 in C9-ALS cells (**Supplementary Fig. 12** and **Supplementary Data 12** and **13**).

8. Similar to Figure 2, in Figure 4, most RNA foci are cytoplasmic, contrary to expectations. Consider adding an image of RNA foci with nuclear staining and a negative control for better understanding.

As described for Point 6, we have updated **Fig. 2** and **Fig. 4** to include new, expanded panels with an increased number of higher-objective immunofluorescent images that better show the nuclear localization of the RNA foci. The panels include a DAPI stain, which helps to further show the nuclear localization of the RNA foci and also individuals channels for the G_4C_2 RNA and EGFP-KASH.

Further, we have updated the manuscript to quantify RNA foci in wild-type C57BL/6J mice, measuring only ~0.08 foci per EGFP-KASH$^+$ cell, a ~10-fold decrease compared to age-matched C9-BACexp mice (**Supplementary Fig. 7** and **Supplementary Table 2**). This difference is consistent with the observations from O'Rourke et al.[1], which reported a ~5- to 8-fold decrease in the percentage of cells positive for the sense foci in C57BL/6J versus C9-BACexp mice. These findings demonstrate that our FISH conditions were selective for the G_4C_2 RNA.

9. Consider conducting an immunoassay of Poly (GP) levels in different brain regions to further support the therapeutic potential of this approach.

We have conducted new studies demonstrating that RfxCas13d-N2V8 decreased poly(GP) protein in C9-BACexp mice. The text on **Pg. 18** now states, "**We next evaluated if RfxCas13d-N2V8 decreased poly(GP), a DPR protein translated from the G_4C_2 repeat RNA and which is produced in C9-BACexp mice[1]. Using a Meso Scale Discovery immunoassay platform[2,13-15], we measured a ~18% decrease in soluble poly(GP) in bulk hippocampal tissue from mice injected with PHP.eB-RfxCas13d-N2V8-crRNA-13 ($P < 0.05$; Fig. 4g). No difference in soluble poly(GP) was measured in bulk cortical tissue, which we attribute to the more limited transduction observed in the MC compared to the HPC**."

10. Are the transcriptomic disease signatures of C9ORF72 ALS in mice or postmortem samples published by previous reports (PMID: 26637796, PMID: 26192745) reversed in crRNA-13 conditions? Also, it is not clear what is the length of time after HEK transduction or mouse injections has passed before RNA-seq is performed. Are these acute effects or longer time scales?

We thank Reviewer 4 for raising this excellent point. We did in fact observe that several of the transcriptomic disease signatures previously identified in C9-BACexp mice were reversed by RfxCas13d in our study. We now elaborate on this in the **Discussion** on **Pg. 24**, where we state, **"…we found by RNA-seq that RfxCas13d-N2V8 reversed a percentage of the transcriptional abnormalities that manifest in C9-BACexp mice, many of**

which were related to pathways previously determined to be dysregulated in C9-BACexp mice[1], including those related to the extracellular matrix, cell junctions, and the immune system."

We have also updated the manuscript to indicate the length of time that elapsed before each RNA-seq measurement. For studies involving HEK293T cells, RNA-seq was conducted at 72 hr post-transfection while, for C9-BACexp mice, RNA-seq was conducted at two-months post-injection. Similarly, we note the length of time elapsed for all RNA-seq studies conducted in iPS neurons (14 days post-transduction).

In summary, several of these concerns should be addressed before this manuscript convincingly demonstrates the impact of the CRISPR-Cas13 platform as a viable treatment for C9ORF72 neuropathies.

We thank Reviewer 4 for their recommendations whose incorporation has strengthened our study.

REFERENCES

1. O'Rourke, J.G. et al. C9ORF72 BAC transgenic mice display typical pathologic features of ALS/FTD. *Neuron* **88**, 892-901 (2015).
2. Liu, Y. et al. Variant-selective stereopure oligonucleotides protect against pathologies associated with C9ORF72-repeat expansion in preclinical models. *Nat. Commun.* **12**, 847 (2021).
3. Martier, R. et al. Targeting RNA-mediated toxicity in C9ORF72 ALS and/or FTD by RNAi-based gene therapy. *Mol. Ther. Nucleic Acids* **16**, 26-37 (2019).
4. Bush, J.A. et al. A blood-brain penetrant RNA-targeted small molecule triggers elimination of r(G(4)C(2))(exp) in c9ALS/FTD via the nuclear RNA exosome. *Proc. Natl. Acad. Sci. U.S.A.* **119**, e2210532119 (2022).
5. Piao, X. et al. Dual-gRNA approach with limited off-target effect corrects C9ORF72 repeat expansion in vivo. *Sci. Rep.* **12**, 5672 (2022).
6. Gilbert, J.W. et al. Identification of selective and non-selective *C9ORF72* targeting *in-vivo* active siRNAs. *Mol. Ther. Nucleic Acids* **35** (2024).
7. Liu, Y. et al. Preclinical evaluation of WVE-004, an investigational stereopure oligonucleotide for the treatment of C9ORF72-associated ALS or FTD. *Mol. Ther. Nucleic Acids* **28**, 558-570 (2022).
8. Kandasamy, P. et al. Impact of guanidine-containing backbone linkages on stereopure antisense oligonucleotides in the CNS. *Nucleic Acids Res.* **50**, 5401-5423 (2022).
9. Meijboom, K.E. et al. CRISPR/Cas9-mediated excision of ALS/FTD-causing hexanucleotide repeat expansion in C9ORF72 rescues major disease mechanisms in vivo and in vitro. *Nat. Commun.* **13**, 6286 (2022).
10. Sachdev, A. et al. Reversal of C9ORF72 mutation-induced transcriptional dysregulation and pathology in cultured human neurons by allele-specific excision. *Proc. Natl. Acad. Sci. U.S.A.* **121**, e2307814121 (2024).
11. Ababneh, N.A. et al. Correction of amyotrophic lateral sclerosis related phenotypes in induced pluripotent stem cell-derived motor neurons carrying a hexanucleotide expansion mutation in C9ORF72 by CRISPR/Cas9 genome editing using homology-directed repair. *Hum. Mol. Genet.* **29**, 2200-2217 (2020).
12. Sareen, D. et al. Targeting RNA foci in iPSC-derived motor neurons from ALS patients with a C9ORF72 repeat expansion. *Sci. Transl. Med.* **5**, 208ra149 (2013).
13. Gendron, T.F. et al. Antisense transcripts of the expanded C9ORF72 hexanucleotide repeat form nuclear RNA foci and undergo repeat-associated non-ATG translation in C9FTD/ALS. *Acta Neuropathol.* **126**, 829-844 (2013).
14. Krishnan, G. et al. Poly(GR) and poly(GA) in cerebrospinal fluid as potential biomarkers for C9ORF72-ALS/FTD. *Nat. Commun.* **13**, 2799 (2022).
15. Simone, R. et al. G-quadruplex-binding small molecules ameliorate C9ORF72 FTD/ALS pathology in vitro and in vivo. *EMBO Mol. Med.* **10**, 22-31 (2018).

16. Ellis, B.L. et al. A survey of ex vivo/in vitro transduction efficiency of mammalian primary cells and cell lines with Nine natural adeno-associated virus (AAV1-9) and one engineered adeno-associated virus serotype. *Virol. J.* **10**, 74 (2013).
17. Duong, T.T. et al. Comparative AAV-eGFP Transgene Expression Using Vector Serotypes 1-9, 7m8, and 8b in Human Pluripotent Stem Cells, RPEs, and Human and Rat Cortical Neurons. *Stem Cells Int.* **2019**, 7281912 (2019).
18. Zu, T. et al. RAN proteins and RNA foci from antisense transcripts in *C9ORF72* ALS and frontotemporal dementia. *Proc. Natl. Acad. Sci. U.S.A.* **110**, E4968-E4977 (2013).
19. Zhang, K. et al. The C9ORF72 repeat expansion disrupts nucleocytoplasmic transport. *Nature* **525**, 56-61 (2015).
20. Jovičić, A. et al. Modifiers of C9ORF72 dipeptide repeat toxicity connect nucleocytoplasmic transport defects to FTD/ALS. *Nat. Neurosci.* **18**, 1226-1229 (2015).
21. Freibaum, B.D. et al. GGGGCC repeat expansion in C9ORF72 compromises nucleocytoplasmic transport. *Nature* **525**, 129-133 (2015).
22. Konermann, S. et al. Transcriptome engineering with RNA-targeting type VI-D CRISPR effectors. *Cell* **173**, 665-676.e614 (2018).
23. Özcan, A. et al. Programmable RNA targeting with the single-protein CRISPR effector Cas7-11. *Nature* **597**, 720-725 (2021).
24. Ai, Y., Liang, D. & Wilusz, J.E. CRISPR/Cas13 effectors have differing extents of off-target effects that limit their utility in eukaryotic cells. *Nucleic Acids Res.* **50**, e65 (2022).
25. Kelley, C.P., Haerle, M.C. & Wang, E.T. Negative autoregulation mitigates collateral RNase activity of repeat-targeting CRISPR-Cas13d in mammalian cells. *Cell Rep.* **40**, 111226 (2022).
26. Li, Y. et al. The collateral activity of RfxCas13d can induce lethality in a RfxCas13d knock-in mouse model. *Genome Biol.* **24**, 20 (2023).
27. Shi, P. et al. Collateral activity of the CRISPR/RfxCas13d system in human cells. *Commun. Biol.* **6**, 334 (2023).
28. Wang, Q. et al. The CRISPR-Cas13a gene-editing system induces collateral cleavage of RNA in glioma cells. *Adv. Sci.* **6**, 1901299 (2019).
29. Shi, P. et al. Collateral activity of the CRISPR/RfxCas13d system in human cells. *Commun. Biol.* **6**, 334 (2023).
30. Li, Y. et al. The collateral activity of RfxCas13d can induce lethality in a RfxCas13d knock-in mouse model. *Genome Biol.* **24**, 20 (2023).
31. Xu, C. et al. Programmable RNA editing with compact CRISPR–Cas13 systems from uncultivated microbes. *Nat. Methods* **18**, 499-506 (2021).
32. Tong, H. et al. High-fidelity Cas13 variants for targeted RNA degradation with minimal collateral effects. *Nat. Biotechnol.* **41**, 108-119 (2023).
33. Chan, K.Y. et al. Engineered AAVs for efficient noninvasive gene delivery to the central and peripheral nervous systems. *Nat. Neurosci.* **20**, 1172-1179 (2017).
34. Maturana, C.J., Verpeut, J.L., Kooshkbaghi, M. & Engel, E.A. Novel tool to quantify with single-cell resolution the number of incoming AAV genomes co-expressed in the mouse nervous system. *Gene Ther.* **30**, 463-468 (2023).
35. Levy, J.M. et al. Cytosine and adenine base editing of the brain, liver, retina, heart and skeletal muscle of mice via adeno-associated viruses. *Nat. Biomed. Eng.* **4**, 97-110 (2020).
36. Lim, C.K.W. et al. CRISPR base editing of cis-regulatory elements enables the perturbation of neurodegeneration-linked genes. *Mol. Ther.* **30**, 3619-3631 (2022).
37. Davis, J.R. et al. Efficient prime editing in mouse brain, liver and heart with dual AAVs. *Nat. Biotechnol.* **42**, 253-264 (2024).
38. Martier, R. et al. Artificial microRNAs targeting C9ORF72 can reduce accumulation of intra-nuclear transcripts in ALS and FTD patients. *Mol. Ther. Nucleic Acids* **14**, 593-608 (2019).

39. Mathiesen, S.N., Lock, J.L., Schoderboeck, L., Abraham, W.C. & Hughes, S.M. CNS transduction benefits of AAV-PHP.eB over AAV9 are dependent on administration route and mouse strain. *Mol. Ther. Methods Clin. Dev.* **19**, 447-458 (2020).

Reviewer #1 (Remarks to the Author):
The manuscript has been improved with more data and discussion. One major concern is still the repeat RNA and DPR quantification, which is the foundation of the whole work. Take RNA foci quantification as an example. There is huge background in the cytoplasm according to several FISH images. Some images are better, but there are actually puncta in the cytoplasm when looking carefully, such as Fig. 4e. How to quantify if it is not clear what is the real signal?

We thank the Reviewer for raising this concern and for the opportunity to improve our images and the description of our methods.

Both **Fig. 2f** and **Fig. 4e** have been updated to include new, higher quality representative images that more clearly indicate the nuclear localization of the RNA foci **in the absence of background**. As part of the reorganization of these panels, the G_4C_2 RNA FISH channel is now merged only with the DAPI channel, **which clearly and unambiguously shows that the foci are nuclear localized and countable** (of note, we observed virtually no foci in the cytoplasm of cells). Previously, we prioritized EGFP-KASH fluorescence and overlap with EGFP-KASH as a focal point for these panels, which contributed background, particularly around the nuclear membrane. This updated panel, which only show a merge between DAPI and the G_4C_2 RNA, with EGFP-KASH fluorescence shown as its own channel, addresses this issue. These images are representative of those used in our analysis. To remove noise from our quantification, all images were despeckled for our analyses, which helped to ensure the most accurate quantification. This is indicated in the Methods. We apologize for not clearly indicated this previously.

To demonstrate the validity of our methods, as mentioned before, we quantified RNA foci in wild-type C57BL/6J mice, measuring only ~0.08 foci per EGFP-KASH$^+$ cell with our conditions, a ~10-fold difference compared to age-matched C9-BACexp mice (**Supplementary Fig. 8** and **Supplementary Table 2**), which was noted before. <u>This difference is consistent with observations from O'Rourke et al.</u>[1], which reported a ~5- to 8-fold decrease in the percentage of cells positive for the sense foci in C57BL/6J versus C9-BACexp mice. Further, we note that our RNA foci quantification methods are similar to numerous other studies that reported their counting procedures[2-4].

Thus, our RNA foci analysis was: (1) well controlled and conducted by a blinded observer, (2) reproducible, as it was conducted with the wild-type RfxCas13d variant and RfxCas13d-N2V8 in multiple brain regions, (3) sufficiently powered to support its conclusions, (4) consistent with the degree of targeting observed by qPCR in the FACS-enriched EGFP-KASH$^+$ cells, (5) conducted on images with limited background, as now clearly indicated in **Fig. 2f** and **Fig. 4e**, and (6) performed in a manner consistent with a number of studies that have described their methods for quantifying RNA foci[2-4].

The concern increases when the GP data does not match the RNA data in mice (Fig. 4c-g). The RNA and RNA foci both reduced significantly in both HPC and MC areas. But it is mentioned in the rebuttal that GP has no change in MC, "which we attribute to the more limited transduction observed in the MC compared to the HPC". If that's the case, the RNA level should show the same.

We appreciate the opportunity to clarify this, as our MSD measurements for the poly(GP) protein in both the HPC and MC are in **<u>full alignment</u>** with the RNA data in our manuscript.

First, it's important to note **that both the V3 and all-V mRNA analyses and the RNA foci analysis were conducted <u>within enriched EGFP-KASH$^+$ cell populations</u>**. In particular, <u>FACS was used to isolate EGFP-KASH$^+$ nuclei for the qPCR analyses</u>, while RNA foci were assessed *only* within the nucleus of EGFP-KASH$^+$ cells. **<u>The poly(GP) protein measurements, by contrast, were conducted in bulk tissue which, as noted in the manuscript and the previous prior Point-by-Point Response, contained a mixture of transduced and non-transduced cells</u>**. In the case of the motor cortex (MC), our immunofluorescence-based measurements indicated that only ~49% of NeuN$^+$ cells in this region were positive for EGFP-KASH$^+$, which is in contrast to the hippocampus (HPC), where we observed that upwards of 90% of the NeuN$^+$ cells were visibly transduced, a nearly two-fold difference that significantly influences the expected possible decrease of the poly(GP) protein between these tissues (it's important to note, also, that the MC and HPC tissue dissections used for the bulk analyses likely contained an increased proportion of non-transduced cells at their periphery, moving away from the injection site).

Second, though our manuscript previously indicated that no difference in poly(GP) was observed in the MC, this conclusion was drawn from a lack of the statistical significance from that measurement. In particular, though the assessment did not meet the threshold for statistical significance, **we in fact observed a ~8% decrease in poly(GP) protein in bulk cortical tissue**, a value consistent with the degree of transduction observed in the MC (~49%) and our qPCR measurements from this area, which showed ~45-50% decrease in V3 in the FACS-enriched EGFP-KASH$^+$ cells. These findings are also in line with our MSD measurements from the HPC, which showed an ~18% decrease in poly(GP) in an area that saw ~87% of the NeuN$^+$ cells transduced with a similar ~50% decrease in V3 in its FACS-enriched EGFP-KASH$^+$ cells.

Rather than just state that no difference in poly(GP) protein was observed in the bulk cortical tissue owing to a lack of statistical significance, we have updated our manuscript to: (i) include these data, which are now shown in **Supplementary Fig. 19**, and (ii) elaborate on an explanation for this dampened effect, while also contextualizing these findings with our results from the enriched cell populations.

To this end, the text on **Pgs. 18-19** now states, "We next evaluated if RfxCas13d-N2V8 decreased poly(GP), a DPR protein translated from the G_4C_2 repeat RNA and which is produced in C9-BACexp mice[1]. Using a Meso Scale Discovery immunoassay platform[5-8], we measured an ~18% decrease in soluble poly(GP) in bulk hippocampal tissue from mice injected with PHP.eB-RfxCas13d-N2V8-crRNA-13 ($P < 0.05$; **Fig. 4g**). **We also measured an ~8% decrease in soluble poly(GP) within bulk cortical tissue (Supplementary Fig. 19), though this result did not meet the threshold for statistical significance for our study ($P > 0.05$). We attribute this weaker effect to the more limited transduction observed in the MC, where only ~49% of the NeuN$^+$ cells in the injected area were found to be positive for EGFP-KASH versus the HPC, where ~87% of the NeuN$^+$ cells in the injected area were EGFP-KASH positive (Supplementary Fig. 14), a nearly two-fold difference. This is particularly meaningful given that the bulk tissue used for these analyses consists of a mixture of transduced and non-transduced cells, with the presence of the latter expected to dampen the measured effect, as observed in the case of the MC. This is in contrast to: (i) our analysis of the V3 mRNA, which was conducted on RNA isolated from EGFP-KASH$^+$ cell populations enriched by FACS, and (ii) our quantification of the RNA foci, which was limited to EGFP-KASH$^+$ cells."**

Our MSD measurements for the poly(GP) for both the HPC and MC are thus in **full alignment** with the RNA data in our manuscript, as explained above. We thank Reviewer 1 for raising this issue, which we clarify with additional data and more explanation.

The authors also did not increase the number of iPSC lines. The resources of C9-ALS iPSC lines have increased a lot in recent years and become more accessible to the community (such as Answer ALS). Including a few different lines is common nowadays. And they cannot measure RNA FISH, GP or glutamate-induced toxicity, which are all well established and used in many publications.

We appreciate the feedback by Reviewer 1. However, we strongly believe that additional studies utilizing multiple different iPSC cell lines are not necessary to support the conclusions of our study. Further, as mentioned before, many prior studies that developed approaches for targeting C9ORF72 and validated targeting in iPSC-derived cells **did so using only a single cell line**. Examples include: Martier et. al.[9], Liu et al. 2021[5], Liu et al. 2022[10], Kandasamy et al.[11], Meijboom et al.[12], and Sachdev et al.[13] Similar to our manuscript, these studies took a holistic approach and evaluated targeting across multiple models, including rodent models and several human cell lines[5,8,9,11,12,14-20]. Nonetheless, this potential limitation was acknowledged in our manuscript, as we modified our **Discussion** on **Pg. 24** to state, **"…we wish to note several important limitations in our iPSC-based study. First, only a single C9-ALS iPSC line was used to evaluate RfxCas13d. Second, we did not determine if RfxCas13d affected the formation of RNA foci, a DPR protein or a toxic phenotype in the C9-ALS neurospheres. Thus, a more detailed study involving multiple iPSC lines from different individuals alongside an expanded suite of measurements will be needed to more generally determine the ability of RfxCas13d to improve deficits in iMNs**."

That said, we do note that RfxCas13d-mediated targeting was measured in a second iPSC-derived cell line, as our manuscript was previously to include measurements from neurospheres derived from wild-type iPSCs, whose V3 transcript is also a target for our platform. This data thus helps to support the reproducibility of our approach. The manuscript on **Pg. 15** now states, "…we determined the relative ratio of V3 to all-V for each sample, finding that C9-ALS neurospheres treated with the PHP.eB-RfxCas13d-N2V8-crRNA-13 vector had an ~40% decrease in this

ratio compared to cells treated with the non-targeting crRNA ($P < 0.05$; **Fig. 3j**). This ratio was also decreased in wild-type neurospheres treated with PHP.eB-RfxCas13d-N2V8-crRNA-13… **As crRNA-13 is not selective for the hexanucleotide repeat expansion and can target the wild-type V3 transcript, these data demonstrate targeting in second iPSC-based cell line, which supports the reproducibility of this approach within this cell culture model**."

As noted in our prior response, we measured poly(GP) in the iPSC neurons. However, we found that the poly(GP) protein in the C9-ALS neurosphere *could not be detected at levels above background* using the MSD immunoassay platform. Further, we were unable to measure signal above for either the RNA FISH or glutamate-induced toxicity. We appreciate that these assessments are well-established in laboratories with substantial expertise in the *in vitro* modeling of C9-ALS, however, we were able to acquire signal for these measurements with the commercially obtained materials used for this study. Further, the derivation of entirely new iPSC-based cell culture models that more effectively recreate phenotypes beyond the transcriptional perturbations observed in the neurospheres used in our study are outside the scope of the current work and not necessary to supports its conclusion. Nonetheless, the data throughout our study are robust, well powered, reproducible in multiple models, consistent with the experimental design in the therapeutics space for C9-ALS, and fully support our conclusions, **while the experimental design for our iPSC-based studies are consistent with similar work in the literature.**

We thank Reviewer 1 for their feedback, which has altogether greatly improved our manuscript.

Reviewer #2 (Remarks to the Author):
In a revised manuscript number NCOMMS-23-59540. Authors have responded to given comments in an adequate way. All the given points explained well and necessary experiments at major critics addressed in a sufficient way. Especially given the comment on Cas7-11 and cellular toxicity not only described in a well established experiment but also provide interesting data to the field. I don't have additional comments and critiques related to given manuscript. It is sufficient for the publication.

We thank Reviewer 2 for their time and valuable feedback.

Reviewer #3 (Remarks to the Author):
The authors have adequately addressed all of the concerns that I raised about this manuscript. I particularly note the strengthening of the paper in their inclusion of RNAseq data from more disease-relevant cell lines.

We thank Reviewer 2 for their valued feedback, which has strengthened our manuscript.

Reviewer #4 (Remarks to the Author):
In the manuscript titled 'A high-fidelity CRISPR-Cas13 system improves abnormalities associated with C9ORF72-linked ALS/FTD', Tristan el al. has proposed a Cas13d-based therapeutic approach. They focused on suppressing C9orf72 transcripts. Using a dual-luciferase reporter screen, effective crRNAs (13, 7, and 1) were identified, reducing V3 mRNA levels by 80-95% with minimal effects on overall transcripts. In vivo experiments in the C9-BACexp mouse model demonstrated successful delivery of RfxCas13d via AAV vectors, leading to significant reduction of V3 mRNA and RNA foci in the hippocampus and motor cortex. The study also investigated high-fidelity variants, specifically RfxCas13d-N2V7 and RfxCas13d-N2V8. RfxCas13d-N2V8 achieved over 90% reduction in V3 expression, showcasing high specificity and minimal off-target effects. This variant also partially reversed transcriptional abnormalities in iPSC-derived motor neuron-like cells and the C9-BACexp mouse model.

In the revised version of manuscript, Tristan el al. has also evaluated potential crRNA with Cas7-11 and addressed the potential concerns indicated in previous version of manuscript. Below are the comments and suggestion on revised manuscripts:

1. The localized administration of AAV appears to limit the broader applicability and efficacy of this therapeutic approach in non-CNS tissues. Could you please clarify how localized injections in the motor cortex and hippocampus offer advantages over systemic delivery methods (such as tail vein or retro-orbital) or intracerebral ventricular (ICV) administration? Any additional data on this would be greatly appreciated.

We thank Reviewer 4 for highlighting the importance of delivery. As our manuscript is a **proof-of-concept** focused on determining the potential of RfxCas13d for targeting the G_4C_2 repeat RNA, we employed a delivery

strategy that, based on the experience of our laboratory, would enable a high-resolution analysis of RfxCas13d-mediated outcomes in the targeted areas. However, our manuscript previously acknowledged the translational hurdles associated with such a strategy and the need for future optimization-based studies to identify an ideal AAV capsid and administration route for accessing (the most) relevant cell populations involved in C9-ALS/FTD.

Nonetheless, we have updated our manuscript to more explicitly elaborate on the motivating factor behind our choice of our delivery strategy and to better indicate how delivery to non-CNS tissues could impact such an optimization. The manuscript on **Pg. 27** now states, **"To ensure its expression in the MC and HPC, two structures in C9-BACexp mice that accumulate RNA foci and produce the DPR protein poly(GP), we chose to locally deliver RfxCas13d, which we reasoned would enable a more straightforward analysis of its outcome(s) for our proof-of-concept, particularly given the degree of expression that can achieved locally in mice by an intracranial injection with only a relatively minimal dose of vector[21]. This delivery strategy, however, is likely not clinically translatable. Thus, as a next step, it will be critical to identify the most effective AAV capsid and administration route for delivering RfxCas13d to most affected cell populations in C9-ALS/FTD in a non-invasive manner. Two such potential administration routes are an intracerebroventricular injection and an intravenous injection, with the latter potentially offering a means to target C9ORF72 in non-nervous system tissue should that prove necessary, though a comparison of these administration routes is outside the scope of this study**. Further, while neurons are principally affected in C9-ALS/FTD, other cell types, including astrocytes[22] and microglia[23], have also been implicated in the pathogenesis of the disorder and thus may also need to be considered in a future optimization."

To avoid incorrectly implying that local delivery provides a translational advantage to any other methods, we have restructured the text on **Pg. 9** to state, "The motor cortex (MC) and hippocampus (HPC) are two structures in C9-BACexp mice that: (i) form RNA foci comprised of the G_4C_2 repeat RNA and (ii) produce measurable quantities of the DPR protein poly(GP)[1]. **To ensure the expression of RfxCas13d in these areas, we chose to locally deliver it using the engineered AAV vector variant PHP.eB[24], reasoning that its local delivery via an intracranial injection would enable a more straightforward analysis of its outcomes in the MC and HPC.**"

As this manuscript was a proof-of-concept centered on determining the applicability of RfxCas13d for C9-ALS/FTD, a goal which is supported by our results, **additional studies to compare local delivery to systemic or ICV injection are outside the scope of the current study**, which we note in our manuscript.

We thank Reviewer 1 for raising this point, which offered us the opportunity to further clarify the text to even better consider delivery.

2. All the crRNAs have been designed against sequences in exon 1a and intron 1, which are present in both the healthy and pathogenic alleles. None of the crRNAs target the G4C2 repeat sequences.

We appreciate the opportunity to further clarify our targeting strategy and the rationale behind the dual-reporter system. Reviewer 4 is correct. Our strategy is not allele-specific and our crRNAs do not target the G_4C_2 motif. Given that: (i) the G_4C_2 motif is also present on the healthy, wild-type allele and thus would likely still be targeted by a G_4C_2-targeting crRNA, (ii) that this motif can exist on other transcripts, and (iii) a G_4C_2-targeting crRNA carries risk for forming secondary structure(s) that could limit its ability to target the G_4C_2 repeat RNA, we chose to target the sequencies flanking the repeat expansion, an approach many other studies[5,9,11,12,14,16-20] have undertaken based on similar reasoning.

Importantly, V1 and V3, which are targeted by our strategy, account for only ~5-15% of the total C9ORF72 transcripts in the brain; thus, though not allele-specific, our approach spares V2, the major source of the C9-L protein and thus is not expected to exacerbate any potential haploinsufficiency.

To better clarify these points beyond our description on **Pg. 5**, the **Discussion** on **Pg. 23** had been updated to state, **"Similar to other approaches for the G_4C_2 repeat RNA[5,9,11,12,14,16-20], we chose to target the sequence(s) flanking the repeat expansion rather than the G_4C_2 motif itself, reasoning that: (i) the G_4C_2 sequence can exist within other transcripts which, if recognized by RfxCas13d, could perturb their expression and potentially lead to off-target effects and (ii) a crRNA targeting the G_4C_2 motif carries risk for forming secondary structure(s) that could impede its ability to target the repeat RNA. While not allele-specific, this approach nonetheless spares V2, the main C9ORF72 transcript, which is currently thought to account for ~85-95% of the C9ORF72 transcripts in the brain[19,25] and whose transcription initiates downstream of the**

repeat. Thus, because V2 lacks the repeat expansion, it's not expected to be targeted by this approach, a hypothesis we tested in HEK293T cells using western blot, which indicated that RfxCas13d did not affect the overall abundance of C9-L, the full-length C9ORF72 protein isoform encoded by V2. Thus, our data indicates this approach is not expected to exacerbate any potential haploinsufficiency."

Our approach is thus: (i) methodologically sound, (ii) consistent with prior targeting strategies that have advanced to clinical testing and (iii) had no effect on what is considered the most abundant C9ORF7 protein product.

As I understand it, the firefly luciferase reporter does not contain the C9orf72 sequence, which is why it is not targeted by any crRNA. The use of this reporter seems less relevant if it does not adequately mimic conditions similar to the healthy allele.

As discussed in the manuscript, **the firefly luciferase reporter is used as an indicator for collateral effects induced by the RfxCas13d protein and thus helped guide the identification of more 'specific' crRNAs**. As indicated on **Pg. 6**, the recognition of a target RNA by RfxCas13d unlocks its ability to indiscriminately *trans*-cleave non-specific bystander RNAs[26-32], which can lead to off-target effects that fortunately can be monitored through a secondary reporter[26,27,31,33-35]. Firefly luciferase was thus not used to model C9-ALS but instead implemented to **help guide the discovery of the crRNAs that appeared to induce the fewest bystander effects**. This reporter worked as designed: even though all of the crRNAs designed to target exon 1a or intron 1 decreased Renilla luciferase expression, we found that all but two of these variants (crRNAs 13 and 1) induced a significant degree ($P < 0.01$) of collateral effects, **as indicated by the decrease in firefly luciferase expression**.

Thus, the firefly luciferase reporter served a critical function: it enabled us to identify crRNAs with minimally detectable collateral effects, a finding supported by our many subsequent measurements. We reiterate that our dual-reporter system does not aim to model the pathogenesis of C9-ALS. This is made clear at multiple points in our manuscript.

Furthermore, the fusion of RLuc at the 5' UTR of the target sequence (C9orf72) raises concerns about the screening system. Since the target sequences are downstream of RLuc, there may be instances where the expression of RLuc remains unaffected. Additionally, as the authors acknowledged, the proximity of the target sequences to the poly(A) tail could influence the stability and/or availability of crRNA binding sites. This is concerning and could potentially impact the screening outcomes of the crRNAs.

We appreciate the concern raised by Reviewer 4. They are correct, we previously updated our manuscript to discuss these and other theoretical concerns as potential confounding factors for our dual-reporter system. That said, our reporter platform functioned as designed and as expected based on the literature[5,14,19]: **all 15 of the crRNAs that were evaluated using the Renilla luciferase reporter were found to decrease its expression by >70%, with 14 of the 15 crRNAs found to reduce it by >95%,** which demonstrates its targetability and functionality.

Our results thus show that there was no instance where Renilla luciferase expression was unaffected. We note that the most selective and potent of these crRNAs were subsequently evaluated in both HEK293T cells and SH-SY5Y cells, where they were found to reduce V3 mRNA by 60-95%, with further additional evaluation in C9-BACexp mice. Our reporter platform thus functioned as designed based on the literature and **enabled us to identify crRNAs that targeted the G_4C_2 repeat RNA both *in vitro* and *in vivo***.

Nonetheless, we have added additional data that shows that modifying the 3' UTR of Renilla luciferase **did not significantly impact** its activity in cells which, if advertently affected, could have limited its sensitivity. The manuscript on **Pg. 6** has been updated to now state, "**Importantly, we observed no significant difference in the activity of the 3' UTR-modified Renilla luciferase variant compared to its unmodified form ($P > 0.05$; Supplementary Fig. 2), indicating that: (i) modifying its 3' UTR did not markedly affect the strength of its signal for this assay and (ii) that this platform possessed sufficient activity to enable our screen."**

Additionally, we reiterate that our dual-reporter system was designed to evaluate: (i) the targeting capabilities and (ii) the collateral effects of the designed crRNAs. This reporter was not designed to model C9-ALS or its genetics, as this approach is not allele-specific. As indicated in the manuscript, this reporter was adapted from a study that fused a similar portion of the C9ORF72 gene to the 3' UTR of Renilla luciferase to identify functional microRNAs, enabling the identification of lead candidates. This UTR-based fusion strategy has since been

utilized by multiple studies to identify ASOs for C9ORF72[5,14]. This reporter was thus previously validated and very well established within the field. To better indicate this, we updated the text on **Pg. 6** to state, "**Notably, this strategy was adapted from a previous study, which similarly fused a region of C9ORF72 to the 3' UTR of Renilla to facilitate the identification of microRNAs for C9ORF72[19], thus providing a proof-of-concept validation for this approach. This reporter has since been used by other studies to identify ASOs for C9ORF72, which further validates the strategy**."

Last, to make the success of the reporter for its ability to validate the functionality of the crRNAs clearer, we have the manuscript at three points:

- On **Pg. 6**, the text now states, "From this screen, we found that all 15 crRNAs decreased Renilla luciferase by >75% at 72 hr post-transfection (P < 0.0001) and that 14 of the 15 crRNAs decreased it by at least 95% (P < 0.0001; **Fig. 1d**), **indicating that each crRNA efficiently targeted Renilla luciferase."**
- **On Pg. 7, the text states, "…**we identified two crRNAs, crRNAs 13 and 1, that decreased Renilla luciferase by ~95% (P < 0.0001) and had no significant effect on firefly luciferase compared to the control (P > 0.05; **Fig. 1d**), **thus indicating they efficiently engaged with their target and exerted no detectable bystander effects**."
- On **Pg. 24**, the text now states, "…as C9ORF72 was fused to the 3' UTR of Renilla luciferase, its theoretically possible that the proximity to its poly(A) signal sequence could influence its stability and/or the availability of certain crRNA binding sites, **though we note that: (i) all 15 crRNAs decreased Renilla luciferase expression by >70% and (ii) 14 of the 15 crRNAs decreased it by >95%, which indicates that our screen was not confounded by such factors and that each crRNA effectively decreased the Renilla luciferase reporter**."

In sum, we utilized a previously validated and now well-established reporter system to screen for crRNA activity, which our controls show is just as active as the native Renilla luciferase transcript. Given this and our data from this assay, **which demonstrates the robust targeting of all 15 crRNAs**, there are no concerns about the ability of the Renilla luciferase reporter to determine crRNA activity.

We thank Reviewer 4 for raising this point, which afforded us an opportunity to further clarify our strategy and the results.

3. In Figure 2f, the G4C2 RNA FISH signal appears diffused and overlapping with EGFP-KASH, making it difficult to clearly identify the RNA foci. Would it be possible to provide higher-quality images that depict more distinguishable differences between the two signals?

Both **Fig. 2f** and **Fig. 4e** have been updated to include new, higher-quality representative images that more clearly indicate the nuclear localization of the RNA foci in the **absence of background signal**, particularly from the nuclear membrane. As part of the reorganization of these panels, the G_4C_2 RNA FISH channel is now merged only with the DAPI channel. **All signals are now clearly distinguishable and show unambiguously the nuclear localization of the foci.** We thank Reviewer 4 for this comment.

4. In Figure 3j, the independent graphs for C9-ALS and wild-type do not allow for a direct comparison between the two groups. Could you please provide a more comparative representation? Additionally, would it be possible to include BMP3 and COL6A5 expression data to better assess any potential off-target effects?

We thank for Reviewer 4 for this feedback. From a data visualization and interpretation perspective, we find that normalizing to the NTG condition for each cell line in **Fig. 3j** is the most intuitive and accessible approach for showing the effect of crRNA-13. To enable the easiest comparison between the two cell lines, the two panels were deliberately sized to have the same y-axis range and the same numbering. **These panels thus allow for an easy direct comparison of the effect of the crRNA in the two cell lines**, which would not be possible if the data were compiled into a one panel given the differences in expression between the two cell lines. However, to address the point raised by Reviewer 3 and enable a more readily apparent comparison for the reader, we have updated the manuscript to directly state the result. The text on **Pg. 15** now states, "**though the effect was not as robust as in the C9-ALS cells, as we observed a ~22% decrease in the V3 to all-V ratio within the wild-type neurospheres (P < 0.05; Fig. 3j)"**

We appreciate the recommendation to further probe the expression of BMP3 and COL6A5 by targeted qPCR but this question is outside the scope of the study and not necessary to support any conclusion in our manuscript. In our manuscript, we thoroughly probe the specificity and tolerability of RfxCas13d using a variety of method, including: (i) the dual-luciferase assay, which measured the collateral cleavage activity of the candidate crRNAs (**Fig. 1d**), (ii) cell viability-based assays to measure toxicity (**Supplementary Fig. 9**) and (iii) RNA-seq, which used to measure transcriptome-wide off-target effects in HEK293T cells (**Fig. 3d-g**), wild-type neurospheres (**Supplementary Data 9 and 10**) and EGFP-KASH$^+$ nuclei from C9-BACexp mice (**Supplementary Data 17, 18 and 19)**, which altogether revealed a favorable specificity profile for the platforms developed in our manuscript.

However, to acknowledge the potential of this point for further study, we have updated **Pg. 16** to state, "When paired with crRNA-13, RfxCas13d-N2V8 was found to perturb the expression of only two genes (BMP3 and COL6A5; >1.25-FC to non-targeted crRNA; FDR-adjusted $P < 0.01$; **Supplementary Data 9** and **10)**, both of which were down-regulated. Interestingly, BMP3 and COL6A5 are both involved in cartilage signaling, **a finding that could benefit from further study, though we note that such an analysis is outside the scope of the current work."**

In summary, several of these concerns must be clarified so that the manuscript can convincingly demonstrate the efficacy of the proposed approach as a viable treatment for C9ORF72 neuropathies.

We thank Reviewer 4 for their feedback, whose comments have been addressed and which in total has strengthened our manuscript. Our study is robust, well powered, reproducible in multiple models, consistent with the experimental design in the therapeutics space for C9-ALS, and fully support the conclusion that RfxCas13d can target the G_4C_2 repeat RNA.

REFERENCES

1. O'Rourke, J.G. et al. C9ORF72 BAC transgenic mice display typical pathologic features of ALS/FTD. *Neuron* **88**, 892-901 (2015).
2. Glineburg, M.R. et al. Enhanced detection of expanded repeat mRNA foci with hybridization chain reaction. *Acta Neuropathol. Commun.* **9**, 73 (2021).
3. Jiang, J. et al. Gain of toxicity from ALS/FTD-linked repeat expansions in C9ORF72 Is alleviated by antisense oligonucleotides targeting GGGGCC-containing RNAs. *Neuron* **90**, 535-550 (2016).
4. Bush, J.A. et al. A blood-brain penetrant RNA-targeted small molecule triggers elimination of r(G(4)C(2))(exp) in c9ALS/FTD via the nuclear RNA exosome. *Proc. Natl. Acad. Sci. U.S.A.* **119**, e2210532119 (2022).
5. Liu, Y. et al. Variant-selective stereopure oligonucleotides protect against pathologies associated with C9ORF72-repeat expansion in preclinical models. *Nat Commun* **12**, 847 (2021).
6. Gendron, T.F. et al. Antisense transcripts of the expanded C9ORF72 hexanucleotide repeat form nuclear RNA foci and undergo repeat-associated non-ATG translation in C9FTD/ALS. *Acta Neuropathol.* **126**, 829-844 (2013).
7. Krishnan, G. et al. Poly(GR) and poly(GA) in cerebrospinal fluid as potential biomarkers for C9ORF72-ALS/FTD. *Nat. Commun.* **13**, 2799 (2022).
8. Simone, R. et al. G-quadruplex-binding small molecules ameliorate C9ORF72 FTD/ALS pathology in vitro and in vivo. *EMBO Mol. Med.* **10**, 22-31 (2018).
9. Martier, R. et al. Targeting RNA-mediated toxicity in C9ORF72 ALS and/or FTD by RNAi-based gene therapy. *Mol. Ther. Nucleic Acids* **16**, 26-37 (2019).
10. Liu, Y. et al. Preclinical evaluation of WVE-004, an investigational stereopure oligonucleotide for the treatment of C9ORF72-associated ALS or FTD. *Mol. Ther. Nucleic Acids* **28**, 558-570 (2022).
11. Kandasamy, P. et al. Impact of guanidine-containing backbone linkages on stereopure antisense oligonucleotides in the CNS. *Nucleic Acids Res.* **50**, 5401-5423 (2022).

12. Meijboom, K.E. et al. CRISPR/Cas9-mediated excision of ALS/FTD-causing hexanucleotide repeat expansion in C9ORF72 rescues major disease mechanisms in vivo and in vitro. *Nat. Commun.* **13**, 6286 (2022).
13. Sachdev, A. et al. Reversal of C9ORF72 mutation-induced transcriptional dysregulation and pathology in cultured human neurons by allele-specific excision. *Proc. Natl. Acad. Sci. U.S.A.* **121**, e2307814121 (2024).
14. Tran, H. et al. Suppression of mutant C9ORF72 expression by a potent mixed backbone antisense oligonucleotide. *Nat Med* **28**, 117-124 (2022).
15. Su, Z. et al. Discovery of a biomarker and lead small molecules to target r(GGGGCC)-associated defects in c9FTD/ALS. *Neuron* **83**, 1043-1050 (2014).
16. Cabrera, G.T. et al. Artificial microRNA suppresses C9ORF72 variants and decreases toxic dipeptide repeat proteins in vivo. *Gene Ther.* (2023).
17. Gilbert, J.W. et al. Identification of selective and non-selective *C9ORF72* targeting *in-vivo* active siRNAs. *Mol. Ther. Nucleic Acids* **35** (2024).
18. Lagier-Tourenne, C. et al. Targeted degradation of sense and antisense C9ORF72 RNA foci as therapy for ALS and frontotemporal degeneration. *Proc. Natl. Acad. Sci. U. S. A.* **110**, E4530-4539 (2013).
19. Martier, R. et al. Artificial microRNAs targeting C9ORF72 can reduce accumulation of intra-nuclear transcripts in ALS and FTD patients. *Mol Ther Nucleic Acids* **14**, 593-608 (2019).
20. Piao, X. et al. Dual-gRNA approach with limited off-target effect corrects C9ORF72 repeat expansion in vivo. *Sci. Rep.* **12**, 5672 (2022).
21. Hudry, E. et al. Efficient Gene Transfer to the Central Nervous System by Single-Stranded Anc80L65. *Molecular Therapy - Methods & Clinical Development* **10**, 197-209 (2018).
22. Fomin, V. et al. The C9ORF72 gene, implicated in amyotrophic lateral sclerosis and frontotemporal dementia, encodes a protein that functions in control of endothelin and glutamate signaling. *Mol. Cell. Biol.* **38** (2018).
23. Lall, D. et al. C9ORF72 deficiency promotes microglial-mediated synaptic loss in aging and amyloid accumulation. *Neuron* **109**, 2275-2291.e2278 (2021).
24. Chan, K.Y. et al. Engineered AAVs for efficient noninvasive gene delivery to the central and peripheral nervous systems. *Nat. Neurosci.* **20**, 1172-1179 (2017).
25. Tran, H. et al. Differential toxicity of nuclear RNA foci versus dipeptide repeat proteins in a drosophila model of C9ORF72 FTD/ALS. *Neuron* **87**, 1207-1214 (2015).
26. Ai, Y., Liang, D. & Wilusz, J.E. CRISPR/Cas13 effectors have differing extents of off-target effects that limit their utility in eukaryotic cells. *Nucleic Acids Res.* **50**, e65 (2022).
27. Kelley, C.P., Haerle, M.C. & Wang, E.T. Negative autoregulation mitigates collateral RNase activity of repeat-targeting CRISPR-Cas13d in mammalian cells. *Cell Rep.* **40**, 111226 (2022).
28. Li, Y. et al. The collateral activity of RfxCas13d can induce lethality in a RfxCas13d knock-in mouse model. *Genome Biol.* **24**, 20 (2023).
29. Shi, P. et al. Collateral activity of the CRISPR/RfxCas13d system in human cells. *Commun. Biol.* **6**, 334 (2023).
30. Wang, Q. et al. The CRISPR-Cas13a gene-editing system induces collateral cleavage of RNA in glioma cells. *Adv. Sci.* **6**, 1901299 (2019).
31. Shi, P. et al. Collateral activity of the CRISPR/RfxCas13d system in human cells. *Commun. Biol.* **6**, 334 (2023).
32. Li, Y. et al. The collateral activity of RfxCas13d can induce lethality in a RfxCas13d knock-in mouse model. *Genome Biol.* **24**, 20 (2023).
33. Kushawah, G. et al. CRISPR-Cas13d induces efficient mRNA knockdown in animal embryos. *Dev. Cell* **54**, 805-817.e807 (2020).

34. Tong, H. et al. High-fidelity Cas13 variants for targeted RNA degradation with minimal collateral effects. *Nat. Biotechnol.* **41**, 108-119 (2023).
35. Özcan, A. et al. Programmable RNA targeting with the single-protein CRISPR effector Cas7-11. *Nature* **597**, 720-725 (2021).

Point-by-Point Response
NCOMMS-23-59540B

Reviewer #1:
The concerns have been properly addressed.

Reviewer #4:
In the revised version of the manuscript, the authors effectively address the comments. They have provided clear explanations and conducted experiments to resolve key concerns. I find no further issues or suggestions for improvement; the manuscript seems appropriate for publication.

We thank the Reviewers for the time and effort that they invested into the review of our manuscript, and for their constructive critiques and suggestions.

Review report on revised manuscript.

In the revised manuscript entitled ‘A high-fidelity CRISPR-Cas13 system improves abnormalities associated with C9ORF72-linked ALS/FTD’, Tristan el al. has proposed a Cas13d-based therapeutic approached and addressed the potential concerns indicated. Below are the responses added to respective comments-

1. Diverse cell types exhibit distinct expression levels and patterns of C9orf72 isoforms, implying that selective targeting may lead to varied impacts across different cells. The absence of a comprehensive approach encompassing both the central nervous system (CNS) and non-CNS tissues in this manuscript raises the potential for limited applicability. To address this, post-treatment assessments of vital organ tissues, such as the liver and lungs, are recommended.

   Per the recommendation of Reviewer 4, we have updated our manuscript to include measurements of the distribution of the PHP.eB vector and the expression of V3, all-V and the antisense RNA in non-CNS tissues which includes the heart, lungs, liver, kidney, and spleen. Based on qPCR, between ~1.1 to ~2.6 vector genomes per diploid genome (vg/dg) were measured in the spinal cord, heart, lungs, liver, kidney, and spleen of C9- BACexp mice injected with PHP.eB-RfxCas13d-N2V8-crRNA-13 and PHP.eB-RfxCas13d-N2V8-NTG (Supplementary Fig. 19), while ~114 and ~152 vg/dg were measured in the HPC and ~25 and ~22 vg/dg were measured in the MC of the same mice (Supplementary Fig. 19). No difference in V3, all-V or the antisense RNA was measured in the spinal cord, heart, lung, liver, kidney and spleen of these animals ($P > 0.05$ for all; Supplementary Fig. 20).

   Comment: The localized administration of AAV appears to limit the broader applicability and efficacy of this therapeutic approach in non-CNS tissues. Could you please clarify how localized injections in the motor cortex and hippocampus offer advantages over systemic delivery methods (such as tail vein or retro-orbital) or intracerebral ventricular (ICV) administration? Any additional data on this would be greatly appreciated.

2. The housing conditions, feeding regimen, and overall environment significantly influence the penetrance of gene expression and, consequently, the disease phenotype in mouse models. The incorporation of behavioral studies would provide valuable insights into potential post-treatment adverse effects, thereby enhancing the overall understanding of the approach's implications.

   To address this important point, body weight, motor coordination, and hindlimb and forelimb grip strength were measured for two months in C9-BACexp mice injected with PHP.eB-RfxCas13d-N2V8-crRNA-13 or PHP.eB- RfxCas13d-N2V8-NTG. No deficits were observed for any assessment in the animals treated with RfxCas13d- N2V8-crRNA-13 versus the non-targeted crRNA (Supplementary Fig. 18).

   Though these findings indicate no discernable adverse effects, we discuss the need for additional studies to more compressively determine the tolerability of this approach. We write on Pg. 26, “...no discernable functional deficits were observed in C9-BACexp mice injected with RfxCas13d-N2V8 and the repeat-targeting crRNA. However, a more comprehensive and longer-term study in mice carrying the normal C9ORF72 allele will be needed to more definitively determine the tolerability of this approach.”

   Comment: I appreciate the authors for providing this valuable information on the C9-BACexp mice..

Other concerns:

1. The term 'gene silence' is used in several instances. Consider using 'down-regulation' for clarity.

   Comment: I thank authors for thoughtful corrections.

2. Further clarification is needed regarding the firefly luciferase, specifically whether it includes 5' and 3' G4C2 flanking regions. Explain the rationale behind fusing RLuc or FLuc at the 5' end rather than the 3' end, and discuss if the proximity of the crRNA target site to polyA might impact RNA stability.

   The manuscript has been updated to better explain the structure of the firefly luciferase reporter. We now state on Pg. 6, "...we used firefly luciferase (Fig. 1c), which was not targeted by any crRNA but whose expression was nonetheless expected to decrease if collaterally *trans*-cleaved by RfxCas13d." As indicated in the main- text and in Fig. 1, only Renilla luciferase, whose 3' UTR was modified to contain a region of C9ORF72, was targeted by the crRNAs.

   We also elaborate on the rationale for fusing C9ORF72 to the 3' UTR of Renilla luciferase, noting that our approach was inspired by a prior study. We write on Pg. 5, "This design was inspired by a study that fused a region of C9ORF72 to the 3' UTR of Renilla to evaluate C9ORF72-targeting microRNAs[38]."

   Finally, we updated the Discussion to speculate on the effects of fusing C9ORF72 to the 3' UTR of Renilla, noting, "...as C9ORF72 was fused to the 3' UTR of Renilla luciferase, we note that its proximity to its poly(A) signal sequence could influence its stability and/or the availability of certain crRNA binding sites. Thus, in the future, it may be more practical to screen crRNAs against the endogenous C9ORF72 transcript variants in order to avoid potential confounding factor(s) associated with the utilization of a reporter."

   Comment: All the crRNAs have been designed against sequences in exon 1a and intron 1, which are present in both the healthy and pathogenic alleles. None of the crRNAs target the G4C2 repeat sequences. As I understand it, the firefly luciferase reporter does not contain the C9orf72 sequence, which is why it is not targeted by any crRNA. The use of this reporter seems less relevant if it does not adequately mimic conditions similar to the healthy allele.

   Furthermore, the fusion of RLuc at the 5' UTR of the target sequence (C9orf72) raises concerns about the screening system. Since the target sequences are downstream of RLuc, there may be instances where the expression of RLuc remains unaffected. Additionally, as the authors acknowledged, the proximity of the target sequences to the poly(A) tail could influence the stability and/or availability of crRNA binding sites. This is concerning and could potentially impact the screening outcomes of the crRNAs

3. Clarify that choice of 20 repeats of G4C2 which are not pathogenic and explain how the dual reporter system represents heterozygosity of the pathogenic allele. Specify if both reporter plasmids are co-transfected with each crRNA.

   The text on Pg. 5 has been updated to indicate that 20 copies of the G_4C_2 repeat were used as it was the longest length that could be commercially synthesized. In addition, heterozygosity if the pathogenic allele was not considered by our reporter which is a limitation of the approach. To indicate this, we updates the Discussion on Pg. 23 to stat, "..targeting the endogenous C9ORF72 transcript variants in the context of the repeat expansion would enable the heterozygosity of the disorder to be considered within the screen."

   Comment: I thank authors for clarification on limitation of approach.

4. Offer rationale for choosing localized administration over intravenous injection of AAV-PHP.eB, despite its proven efficacy when delivered intravenously (PMID: 28671695).

   The text on Pg. 9 has been updated to better explain our rationale for using local delivery rather than systemic for PHP.eB. We write, "To maximize delivery, we chose to inject RfxCas13d directly to the motor cortex (MC) and hippocampus (HPC), two regions of the brain in C9-BACexp mice that form RNA foci comprised of the G_4C_2 repeat RNA and produce measurable quantities of the DPR protein poly(GP)[1]. Though most commonly administered intravenously, we used the AAV vector variant PHP.eB[33] to deliver

RfxCas13d, as it can transduce cells to a similar extent as AAV9 when injected locally to the brain[39] but, in the experience of our laboratory, packages at a higher titer."

Comment: As indicated in point 1, the preference for localized administration over intravenous or intracerebral ventricular (ICV) delivery introduces several limitations

5. In Figure 2f, clarify whether differences in magnification or cell size contribute to the observation that most RNA foci are cytoplasmic. Address the surprising cytoplasmic localization, which contrasts with expectations (PMID: 26637796). Consider adding an image of RNA foci with nuclear staining for better visualization.

As discussed for Reviewer 1, we have updated the manuscript to include new panels with an increased number of higher-objective immunofluorescent images that better show the nuclear localization of the RNA foci in C9- BACexp mice. In particular, in addition to providing higher resolution merge images, these panels show the individual channels for the G_4C_2 RNA, EGFP-KASH, and DAPI, which altogether better demonstrate the nuclear localization of the RNA foci. These panels are present in Fig. 2f and Fig. 4e.

Comment: In Figure 2f, the G4C2 RNA FISH signal appears diffused and overlapping with EGFP-KASH, making it difficult to clearly identify the RNA foci. Would it be possible to provide higher-quality images that depict more distinguishable differences between the two signals?

Improve clarity and labeling in Figure 3b and c. The blue and white colors are reversed for All-V and V3.

We thank Reviewer 4 for identifying this error. We have corrected the blue and white colors for legends in Fig. 3b and 3c.

Comment: I appreciate authors for correction they made.

6. Figure 3 lacks a control using cells derived from a non-ALS individual.

As discussed for Reviewer 1, we have expanded the iPSC-based studies in the manuscript to include measurements for neurospheres derived from a non-ALS individual. As shown in Fig. 3j, we measured in these cells a decrease ($P < 0.05$) in the relative V3-to-all-V ratio following their treatment with RfxCas13d-N2V8, which is consistent with our findings in the C9-ALS neurospheres.

We have also used these cells to conduct additional RNA-seq studies to demonstrate a disease-linked profile in the C9-ALS neurospheres, identifying 2,470 genes that were dysregulated in the C9-ALS cells (>1.25 FC relative to the non-ALS cells at 14 days *in vitro*; FDR P-value < 0.01; Supplementary Fig. 10a and Supplementary Data 6 and 7). Further, we used these cells to determine RfxCas13d targeting specificity. We find that, when paired with crRNA-13, RfxCas13d-N2V8 perturbed the expression of only two genes (BMP3 and COL6A5) compared to cells treated with the non-targeted crRNA (>1.25-FC; FDR-adjusted P < 0.01; Supplementary Data 9 and 10). Both genes were down-regulated (Supplementary Data 10) and are involved in cartilage signaling.

Last, we used cells from the non-ALS individual to bolster the reversion analysis in the iPS neurons (Fig. 3l and Supplementary Data 11), including RNA-seq, which was conducted to determine the transcriptome-wide effects of RfxCas13d-N2V8 in C9-ALS cells (Supplementary Fig. 12 and Supplementary Data 12 and 13).

Comment: In Figure 3j, the independent graphs for C9-ALS and wild-type do not allow for a direct comparison between the two groups. Could you please provide a more comparative representation? Additionally, would it be possible to include BMP3 and COL6A5 expression data to better assess any potential off-target effects?

7. Similar to Figure 2, in Figure 4, most RNA foci are cytoplasmic, contrary to expectations. Consider adding an image of RNA foci with nuclear staining and a negative control for better understanding.

As described for Point 6, we have updated Fig. 2 and Fig. 4 to include new, expanded panels with an increased number of higher-objective immunofluorescent images that better show the nuclear localization of the RNA foci. The panels include a DAPI stain, which helps to further show the nuclear localization of the RNA foci and also individual channels for the G_4C_2 RNA and EGFP-KASH.

Further, we have updated the manuscript to quantify RNA foci in wild-type C57BL/6J mice, measuring only ~0.08 foci per EGFP-KASH$^+$ cell, a ~10-fold decrease compared to age-matched C9-BACexp mice (Supplementary Fig. 7 and Supplementary Table 2). This difference is consistent with the observations from O'Rourke et al.[1], which reported a ~5- to 8-fold decrease in the percentage of cells positive for the sense foci in C57BL/6J versus C9-BACexp mice. These findings demonstrate that our FISH conditions were selective for the G_4C_2 RNA.

Comment: I appreciate authors for updates and additional information.

8. Consider conducting an immunoassay of Poly (GP) levels in different brain regions to further support the therapeutic potential of this approach.

We have conducted new studies demonstrating that RfxCas13d-N2V8 decreased poly(GP) protein in C9-BACexp mice. The text on Pg 18 now states, "We next evaluated if RfxCas13d-N2V8 decreased poly(GP), a DPR protein translated from the G_4C_2 repeat RNA and which is produced in C9-BACexp mice[1]. Using a Meso Scale Discovery immunoassay platform[2,13-15], we measured a ~18% decrease in soluble poly(GP) in bulk hippocampal tissue from mice injected with PHP.eB-RfxCas13d-N2V8-crRNA-13 ($P < 0.05$; Fig. 4g). No difference in soluble poly(GP) was measured in bulk cortical tissue, which we attribute to the more limited transduction observed in the MC compared to the HPC."

Comment: I appreciate authors for the addition of data on immunoassay of Poly(GP).

9. Are the transcriptomic disease signatures of C9ORF72 ALS in mice or postmortem samples published by previous reports (PMID: 26637796, PMID: 26192745) reversed in crRNA-13 conditions? Also, it is not clear what is the length of time after HEK transduction or mouse injections has passed before RNA-seq is performed. Are these acute effects or longer time scales?

We thank Reviewer 4 for raising this excellent point. We did in fact observe that several of the transcriptomic disease signatures previously identified in C9-BACexp mice were reversed by RfxCas13d in our study. We now elaborate on this in the Discussion on Pg. 24, where we state, "...we found by RNA-seq that RfxCas13d-N2V8 reversed a percentage of the transcriptional abnormalities that manifest in C9-BACexp mice, many of which were related to pathways previously determined to be dysregulated in C9-BACexp mice1, including those related to the extracellular matrix, cell junctions, and the immune system."

We have also updated the manuscript to indicate the length of time that elapsed before each RNA-seq measurement. For studies involving HEK293T cells, RNA-seq was conducted at 72 hr post-transfection while, for C9-BACexp mice, RNA-seq was conducted at two-months post-injection. Similarly, we note the length of time elapsed for all RNA-seq studies conducted in iPS neurons (14 days post-transduction).

Comment: I appreciate authors for additional information on global gene expression rescue studies.
